# The global burden of childhood and adolescent cancer in 2017: an analysis of the Global Burden of Disease Study 2017

**DOI:** 10.1016/S1470-2045(19)30339-0

**Published:** 2019-09

**Authors:** GBD 2017 Childhood Cancer Collaborators, Lisa M. Force, Ibrahim Abdollahpour, Shailesh M Advani, Dominic Agius, Elham Ahmadian, Fares Alahdab, Tahiya Alam, Animut Alebel, Vahid Alipour, Christine A. Allen, Amir Almasi-Hashiani, Elysia M Alvarez, Saeed Amini, Yaw Ampem Amoako, Nahla Hamed Anber, Jalal Arabloo, Al Artaman, Suleman Atique, Ashish Awasthi, Mojtaba Bagherzadeh, Huda Basaleem, Eyasu Tamru Bekru, Ali Bijani, Kassawmar Angaw Bogale, Mate Car, Félix Carvalho, Clara Castro, Ferrán Catalá-López, Dinh-Toi Chu, Vera M Costa, Amira Hamed Darwish, Feleke Mekonnen Demeke, Asmamaw Bizuneh Demis, Gebre Teklemariam Demoz, Samath Dhamminda Dharmaratne, Huyen Phuc Do, Linh Phuong Doan, Manisha Dubey, Aziz Eftekhari, Ziad El-Khatib, Mohammad Hassan Emamian, Mahdieh Abbasalizad Farhangi, Eduarda Fernandes, Florian Fischer, Reza Fouladi Fard, Paola M Friedrich, Takeshi Fukumoto, Getnet Azeze Gedefaw, Ahmad Ghashghaee, Asadollah Gholamian, Arvin Haj-Mirzaian, Arya Haj-Mirzaian, Samer Hamidi, James D. Harvey, Hamid Yimam Hassen, Simon I. Hay, Chi Linh Hoang, Michael K. Hole, Nobuyuki Horita, Seyyed Nasrollah Hosseini, Mehdi Hosseinzadeh, Mohamed Hsairi, Melissa Maria Hudson, Kaire Innos, Farzad Jalilian, Spencer L. James, Amir Kasaeian, Tesfaye Dessale Kassa, Nicholas J Kassebaum, Peter Njenga Keiyoro, Yousef Saleh Khader, Jagdish Khubchandani, Neda Kianipour, Jeannette Kirby, Adnan Kisa, Sezer Kisa, Jonathan M Kocarnik, Paolo Lauriola, Alan D Lopez, Margit Mägi, Manzoor Ahmad Malik, Ali Manafi, Navid Manafi, Mohammad Ali Mansournia, Benjamin Ballard Massenburg, Varshil Mehta, Hagazi Gebre Meles, Tuomo J Meretoja, Tomislav Mestrovic, Seyed Mostafa Mir, Mehdi Mirzaei-Alavijeh, Dara K. Mohammad, Aso Mohammad Darwesh, Naser Mohammad Gholi Mezerji, Roghayeh Mohammadibakhsh, Milad Mohammadoo-Khorasani, Ali H Mokdad, Yoshan Moodley, Mahmood Moosazadeh, Maryam Moossavi, Farhad Moradpour, Shane Douglas Morrison, Kindie Fentahun Muchie, Mohsen Naghavi, Javad Nazari, Josephine W. Ngunjiri, Cuong Tat Nguyen, Long Hoang Nguyen, Son Hoang Nguyen, Trang Huyen Nguyen, Molly R Nixon, Andrew T Olagunju, Tinuke O Olagunju, Sok King Ong, Keiu Paapsi, Alyssa Pennini, David M. Pereira, Julian David Pillay, Mostafa Qorbani, Mohammad Rabiee, Navid Rabiee, Samira Raoofi, David Laith Rawaf, Salman Rawaf, Robert C Reiner, Nima Rezaei, Aziz Rezapour, Kedir Teji Roba, Les L. Robison, Carlos Rodriguez-Galindo, Gholamreza Roshandel, Saeid Safiri, Mohamadreza Salahshoor, Saleh Salehi Zahabi, Abdallah M. Samy, Milena M Santric Milicevic, Maheswar Satpathy, Susan M Sawyer, Seyedmojtaba Seyedmousavi, Hosein Shabaninejad, Masood Ali Shaikh, Amir Shamshirian, Morteza Shamsizadeh, Reza Shirkoohi, Soraya Siabani, Jasvinder A. Singh, Khairil SiRamlee, Rafael Tabarés-Seisdedos, Mohamad-Hani Temsah, Bach Xuan Tran, Irfan Ullah, Amir Vahedian-Azimi, Stein Emil Vollset, Theo Vos, Yasir Waheed, Girmay Teklay Weldesamuel, Hailemariam Mekonnen Workie, Rixing Xu, Mehdi Yaseri, Naohiro Yonemoto, Chuanhua Yu, Vesna Zadnik, Telma Zahirian Moghadam, Zoubida Zaidi, Alireza Zangeneh, Taye Abuhay Zewale, Arash Ziapour, Sanjay Zodpey, Christopher J L Murray, Nickhill Bhakta, Christina Fitzmaurice

## Abstract

**Background:**

Accurate childhood cancer burden data are crucial for resource planning and health policy prioritisation. Model-based estimates are necessary because cancer surveillance data are scarce or non-existent in many countries. Although global incidence and mortality estimates are available, there are no previous analyses of the global burden of childhood cancer represented in disability-adjusted life-years (DALYs).

**Methods:**

Using the Global Burden of Diseases, Injuries, and Risk Factors Study (GBD) 2017 methodology, childhood (ages 0–19 years) cancer mortality was estimated by use of vital registration system data, verbal autopsy data, and population-based cancer registry incidence data, which were transformed to mortality estimates through modelled mortality-to-incidence ratios (MIRs). Childhood cancer incidence was estimated using the mortality estimates and corresponding MIRs. Prevalence estimates were calculated by using MIR to model survival and multiplied by disability weights to obtain years lived with disability (YLDs). Years of life lost (YLLs) were calculated by multiplying age-specific cancer deaths by the difference between the age of death and a reference life expectancy. DALYs were calculated as the sum of YLLs and YLDs. Final point estimates are reported with 95% uncertainty intervals.

**Findings:**

Globally, in 2017, there were 11·5 million (95% uncertainty interval 10·6–12·3) DALYs due to childhood cancer, 97·3% (97·3–97·3) of which were attributable to YLLs and 2·7% (2·7–2·7) of which were attributable to YLDs. Childhood cancer was the sixth leading cause of total cancer burden globally and the ninth leading cause of childhood disease burden globally. 82·2% (82·1–82·2) of global childhood cancer DALYs occurred in low, low-middle, or middle Socio-demographic Index locations, whereas 50·3% (50·3–50·3) of adult cancer DALYs occurred in these same locations. Cancers that are uncategorised in the current GBD framework comprised 26·5% (26·5–26·5) of global childhood cancer DALYs.

**Interpretation:**

The GBD 2017 results call attention to the substantial burden of childhood cancer globally, which disproportionately affects populations in resource-limited settings. The use of DALY-based estimates is crucial in demonstrating that childhood cancer burden represents an important global cancer and child health concern.

**Funding:**

Bill & Melinda Gates Foundation, American Lebanese Syrian Associated Charities (ALSAC), and St. Baldrick's Foundation.

## Introduction

Children with cancer who live in high-income countries (HICs) have good outcomes, with approximately 80% surviving 5 years after their diagnosis.^1^, ^2^ However, more than 90% of children at risk of developing childhood cancer each year live in low-income and middle-income countries (LMICs).^3^, ^4^, ^5^ Considered by many as one of the major advances of modern science, the improvement in outcomes in children with cancer seen in HICs over the past several decades has not translated to most LMICs, where existing data suggest that far fewer children survive.^6^ An accurate appraisal of childhood cancer incidence and outcomes is non-existent in many LMICs, due in part to a lack of the cancer registry and vital registration systems necessary to record and report these data.^5^, ^7^ Childhood cancers are often fatal without appropriate and timely diagnosis and treatment and, by contrast with adult cancers, there are no evidence-based population screening programmes or lifestyle risk-reduction strategies that are effective in improving outcomes.^8^, ^9^ As a result, increasing survival will require considerable planning by policy makers to ensure adequate resource allocation and health system function. Information on the burden of childhood cancer is crucial to informing these efforts and thus, model-based estimates are necessary to determine cancer burden in settings without data until cancer data coverage improves.

The Global Burden of Diseases, Injuries, and Risk Factors Study (GBD) 2017 provides estimates for 359 diseases and injuries, including cancers, and is therefore uniquely positioned to fill the gap in health planning data as countries work to expand their cancer surveillance systems.^10^ Additionally, standard GBD outcomes include estimates of disability-adjusted life-years (DALYs), a useful composite metric that accounts for both the mortality and morbidity of a disease.^11^ DALYs allow for cross-disease and cross-geography comparisons that contextualise disease burden. So far, however, no dedicated GBD analysis of childhood cancer burden has been done. Previous research describing childhood cancer burden internationally has focused on traditional metrics of cancer burden, including incidence, mortality, and survival.^6^, ^7^, ^12^ We aimed to report the global burden of childhood cancer in 2017 using DALY estimates from GBD 2017, an approach that adds a new perspective to the assessment of childhood cancer burden than has been presented in previous analyses.

**Research in context**

**Evidence before this study**

Previous work to describe childhood cancer burden globally has focused on conventional metrics of cancer burden, such as incidence, mortality, and survival, either in a subset of countries (eg, the third volume of the International Incidence of Childhood Cancer and CONCORD-3) or globally (eg, GLOBOCAN 2018). We searched PubMed for English-language research articles describing the global burden of childhood cancer published between Jan 1, 2010, and Sept 30, 2018, using the terms “pediatric or childhood or child” and “cancer or neoplasm or tumor or malignancy or oncology” and “global or international or worldwide or world” and “burden or metrics or incidence or mortality or prevalence or survival” but did not find additional applicable work. While providing valuable information, no previous publications incorporated morbidity or provided disability-adjusted life-years (DALYs), a metric that allows policy makers to directly compare the lifelong implications of childhood cancer burden against other diseases for priority setting.

**Added value of this study**

To our knowledge, we report for the first time the global and regional estimates of childhood cancer burden using Global Burden of Diseases, Injuries, and Risk Factors Study (GBD) 2017 results, with DALYs as the outcome measure, providing a new perspective on the global burden childhood cancer to that previously available in published literature. The global DALY burden due to childhood cancers in 2017 is substantial, primarily because of fatal burden. This burden is disproportionately high in low, low-middle, and middle Socio-demographic Index (SDI) settings, which together contribute 82·2% of global childhood cancer DALYs. Childhood cancers are a major cause of global disease burden, even when compared with other diseases of childhood or with adult cancers.

**Implications of all the available evidence**

By presenting the global burden of childhood cancer in DALYs, we identified that childhood cancer results in a substantial disease burden despite a relatively low absolute number of incident cases and deaths. This burden is particularly notable in resource-limited settings, where the ability to directly compare the burden of various diseases through DALYs is particularly relevant for policy makers, who must consider a myriad of health priorities in addition to childhood cancers and can use these data to make evidence-based resource allocation and cancer-control planning decisions. As countries implement, monitor, and evaluate capacity-building programmes as part of the WHO Global Initiative for Childhood Cancer, refining the methodology of childhood cancer burden estimation in future GBD iterations will be crucial to identify high-impact interventions and provide the most useful information for cancer control efforts by governments, stakeholders, and the global health community.

## Methods

### Overview

The GBD study was created to establish comprehensive and comparable global health metrics. Estimates of incidence, prevalence, mortality, years of life lost (YLLs), years lived with disability (YLDs), and DALYs are generated for each disease and injury, with each metric reported by year, location, age group, and sex.^13^ Each successive GBD iteration supersedes the results of previous GBD rounds for the entire newly estimated time series. GBD 2017 is compliant with the Guidelines for Accurate and Transparent Health Estimates Reporting (appendix p 4).^14^ Data sources used in GBD 2017 are available online.

### Estimation of cancer burden

The GBD cancer estimation process focuses first on the estimation of cancer mortality (see appendix pp 7–8 for flow diagrams of the GBD 2017 cancer estimation process). Cancer mortality data sources include vital registration systems, cancer registration systems, and verbal autopsy data (a map of the site-years of childhood cancer data available in GBD 2017 is available on appendix p 10). Cancer registries are active in some locations that do not have reliable cancer mortality data, and many cancer registries only report incidence. Thus, mortality-to-incidence ratios (MIRs) were used to transform cancer registry incidence data to mortality estimates, maximising data availability in locations with scarce mortality information. MIRs for all age, sex, location, and year combinations were modelled using a spatiotemporal Gaussian process regression with incidence data from cancer registries and mortality data from cancer registries or high-quality vital registration systems. In brief, spatiotemporal Gaussian process regression has three steps: logit random effects models, spatiotemporal smoothing, and Gaussian process regression (appendix p 13; see also the supplementary materials for reference 15).^15^ The mortality estimates derived with this approach were pooled with the directly obtained mortality data from vital registration systems and verbal autopsies, and used in cancer-specific Cause of Death Ensemble models (CODEm), which are necessary because mortality data do not exist for every age, sex, location, and year combination estimated by GBD 2017.^16^ The CODEm approach uses all available mortality data even if data quality varies, tests individual as well as ensemble models, and is capable of selecting the optimal model or set of models on the basis of the out-of-sample predictive validity. Each CODEm used covariates and age group restrictions specific to each cancer type (appendix pp 15–34). Cause-specific mortality estimates were subsequently scaled to independently modelled all-cause mortality.^17^, ^18^

The mortality estimates for each cancer type were divided by the corresponding MIR to obtain incidence estimates. 10-year prevalence was modelled using estimated survival based on the MIR. Total prevalence was divided into sequelae (phases of cancer treatment) to estimate the cancer type-specific YLDs (appendix p 34). Two sequelae were estimated for cohorts that survive 10 years after diagnosis: (1) diagnosis or treatment and (2) remission, after which disability risk is returned to that of the general population. Four sequelae were estimated for cohorts that do not survive 10 years after diagnosis: (1) diagnosis or treatment, (2) remission, (3) metastatic or disseminated, and (4) terminal phases. To generate YLD estimates, each sequela prevalence was multiplied by a sequelae-specific disability weight, representing the magnitude of health loss associated with a specific health outcome, measured on a scale from 0 (full health) to 1 (equivalent to death; appendix p 39).^19^ YLLs were estimated by multiplying the difference between a standard life expectancy at the age of death and the estimated number of deaths at that age.^17^ The YLD and YLL estimates were summed to provide DALY estimates.^13^ More detailed descriptions of the methods for disease burden estimation can be found in the appendix for this paper and in the GBD 2017 capstone publications.^13^, ^17^, ^18^, ^19^

### Definitions

The childhood age group in this analysis encompasses children and adolescents, defined as ages 0–19 years. The 0–14-year age range is used to define paediatrics in some countries and global health organisations, and data for subsets of this age range are available online using the GBD Compare Tool and the GBD Results Tool. All cancers as defined in the 10th revision of the International Classification of Diseases, chapter II (neoplasms), are included in the GBD cancer estimation process (appendix p 10). Only malignant neoplasms were included in this analysis; non-melanoma skin cancers were excluded. In this analysis, we restructured the cancer diagnostic categories to depict the most relevant childhood cancer information, categorising any cancer with less than 1000 global deaths annually as other rare cancers, and any cancer without a specific GBD cause as uncategorised cancers. All rates in this paper are reported per 100 000 person-years, with the GBD 2017 world standard population used for calculation of age-standardised rates.^17^ See the appendix for definitions of GBD world super-regions (p 54) and GBD world regions (p 60).

GBD 2017 produced estimates at global, regional, national, and select subnational levels; ^13^ this analysis focuses on the global and regional estimates. Country and subnational estimates are available online using the GBD Compare and GBD Results tools. Results are presented by Socio-demographic Index (SDI) quintile in a subset of tables and figures given the usefulness of SDI as a summary measure of where countries are on the development spectrum (appendix p 47). SDI is a composite measure of income per capita, total fertility rate under 25 years of age, and average educational attainment, and has been shown to correlate well with health outcomes.^19^

### Uncertainty analysis

Final point estimates are reported with 95% uncertainty intervals (UIs). The UIs were calculated as the 2·5th and 97·5th percentile of the distribution of 1000 draws at each step in the cancer estimation process, with the uncertainty propagated through each step (UI estimation is described in further detail in the appendix p 39).

### Role of the funding source

The funders of this research had no role in the design of the GBD cancer estimation process, collection or analysis of data, interpretation of results, or in the writing of this manuscript. The corresponding author had full access to all data used in this study and had final responsibility for the decision to submit for publication.

## Results

**Figure 1:**
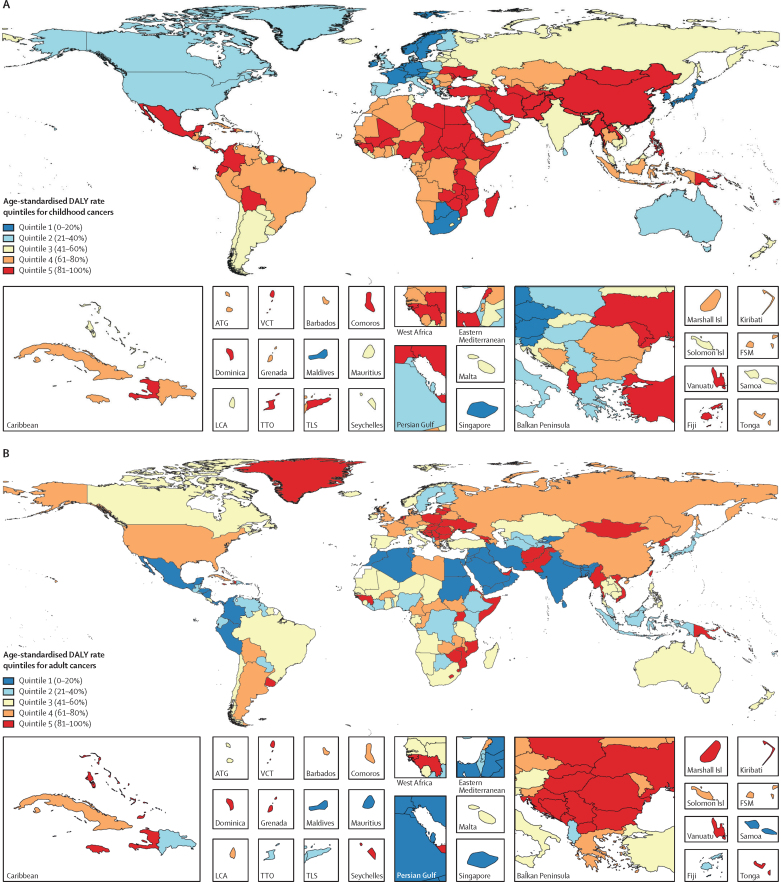
Global map of age-standardised DALY rates for (A) childhood cancers (ages 0–19 years) and (B) adult cancers (20 years or older), both sexes combined, 2017 Quintiles are based on DALYs per 100 000 person-years. For childhood cancers, quintile 1 indicates less than 222, quintile 2 indicates 222 to less than 263, quintile 3 indicates 263 to less than 346, quintile 4 indicates 346 to less than 441, and quintile 5 indicates 441 or more. For adult cancers, quintile 1 indicates less than 3314, quintile 2 indicates 3314 to less than 3915, quintile 3 indicates 3915 to less than 4407, quintile 4 indicates 4407 to less than 4964, and quintile 5 indicates 4964 or more. Adult cancer burden portrayed in this figure excluded non-melanoma skin cancers and benign tumours in order to be comparable to the childhood cancer burden map. ATG=Antigua and Barbuda. DALY=disability-adjusted life-year. FSM=Federated States of Micronesia. Isl=Islands. LCA=Saint Lucia. TLS=Timor-Leste. TTO=Trinidad and Tobago. VCT=Saint Vincent and the Grenadines.

Childhood cancer resulted in 11·5 million (95% UI 10·6–12·3) DALYs globally in 2017, of which 97·3% (97·3–97·3) came from YLLs and 2·7% (2·7–2·7) came from YLDs (table). A substantial portion of the global burden of childhood cancer exists in low, low-middle, and middle SDI countries (82·2% [82·1–82·2] of the global childhood cancer total DALYs; table), countries that are concentrated in Asia, Africa, and Central and South America (figure 1A). This geographical pattern of cancer burden distribution is noticeably different from that observed in adults (figure 1B), with only 50·3% (50·3–50·3) of the global adult cancer absolute DALY burden affecting low, low-middle, and middle SDI countries (appendix p 66).

**Table:** Childhood cancer burden, 2017

		**Absolute incidence (95% UI)**	**Age-standardised incidence rate (95% UI)**	**Absolute mortality (95% UI)**	**Age-standardised mortality rate (95% UI)**	**Absolute YLLs (95% UI)**	**Absolute YLDs (95% UI)**	**Absolute DALYs (95% UI)**
Global		416 500 (384 900–442 100)	16·2 (15·0–17·2)	142 300 (131 500–151 900)	5·5 (5·1–5·9)	11 236 500 (10 380 800–12 005 700)	313 100 (209 600–449 100)	11 549 600 (10 649 900–12 334 700)
SDI status								
	High SDI countries	49 700 (46 200–53 800)	20·8 (19·3–22·5)	6700 (6300–7200)	2·7 (2·6–2·9)	523 100 (489 100–555 400)	49 700 (32 200–72 900)	572 800 (530 700–615 400)
	High-middle SDI countries	97 600 (83 100–106 000)	30·1 (25·5–32·7)	16 900 (15 300–17 900)	5·1 (4·6–5·4)	1 326 100 (1 191 800–1 404 000)	129 900 (78 400–206 200)	1 456 000 (1 293 900–1 580 500)
	Middle SDI countries	107 300 (98 500–115 500)	17·1 (15·7–18·5)	31 900 (29 800–33 700)	5·0 (4·6–5·3)	2 493 900 (2 321 600–2 636 400)	68 800 (45 500–97 700)	2 562 600 (2 380 200–2 711 800)
	Low-middle SDI countries	91 700 (81 600–102 000)	12·6 (11·2–14·1)	48 300 (42 900–54 000)	6·6 (5·9–7·4)	3 821 300 (3 385 500–4 278 800)	36 000 (25 000–49 400)	3 857 300 (3 412 700–4 320 000)
	Low SDI countries	67 900 (61 400–74 100)	10·5 (9·5–11·5)	38 000 (34 300–41 500)	5·9 (5·3–6·4)	3 042 300 (2 738 200–3 317 500)	26 000 (18 100–34 400)	3 068 400 (2 763 800–3 345 100)
Cancers								
	Global acute lymphoblastic leukaemia	59 100 (50 000–66 700)	2·3 (2·0–2·6)	18 700 (16 600–21 100)	0·7 (0·6–0·8)	1 479 400 (1 308 800–1 665 100)	25 700 (17 600–36 200)	1 505 100 (1 331 700–1 701 100)
	Global acute myeloid leukaemia	22 000 (18 700–24 400)	0·9 (0·7–1·0)	10 400 (8800–11 700)	0·4 (0·3–0·5)	827 100 (699 800–921 900)	4900 (3400–6600)	832 000 (704 500–928 500)
	Global leukaemias not otherwise specified*	68 400 (56 900–77 800)	2·7 (2·2–3·1)	19 700 (16 700–22 100)	0·8 (0·6–0·9)	1 565 900 (1 323 600–1 750 800)	30 000 (19 700–42 000)	1 595 900 (1 347 800–1 780 800)
	Global non-Hodgkin lymphomas	29 500 (26 700–32 600)	1·1 (1·0–1·3)	14 000 (12 500–15 700)	0·5 (0·5–0·6)	1 113 200 (987 500–1 253 000)	12 100 (8400–16 800)	1 125 300 (997 000–1 267 300)
	Global Hodgkin lymphomas	14 700 (12 200–17 100)	0·6 (0·5–0·6)	4500 (3500–5400)	0·2 (0·1–0·2)	343 600 (268 400–417 100)	6900 (4600–9500)	350 400 (273 800–425 800)
	Global brain and nervous system cancers	67 400 (58 400–76 400)	2·6 (2·3–3·0)	25 800 (22 300–29 500)	1·0 (0·9–1·1)	2 056 900 (1 774 400–2 353 300)	31 300 (21 400–43 500)	2 088 300 (1 802 700–2 389 500)
	Global liver cancers	3500 (3200–3800)	0·1 (0·1–0·1)	2600 (2400–2800)	0·1 (0·1–0·1)	197 000 (181 000–213 900)	800 (600–1000)	197 800 (181 800–214 700)
	Global renal cancers	24 400 (21 900–26 800)	1·0 (0·9–1·1)	3100 (2800–3300)	0·1 (0·1–0·1)	252 500 (229 200–275 800)	10 500 (6700–15 500)	262 900 (237 800–287 500)
	Global other rare cancers†	29 400 (27 600–31 200)	1·1 (1·0–1·1)	7200 (6800–7600)	0·3 (0·3–0·3)	514 000 (485 400–540 800)	16 100 (11 400–21 600)	530 100 (500 500–558 700)
	Global uncategorised cancers‡	98 300 (89 200–106 400)	3·8 (3·5–4·2)	36 200 (32 600–39 700)	1·4 (1·3–1·5)	2 886 900 (2 590 600–3 171 600)	174 900 (108 400–267 100)	3 061 800 (2 752 800–3 367 000)

Absolute incidence, mortality, YLLs, YLDs, and DALYs represent the total childhood cancer (0–19 years, both sexes combined) values, rounded to the nearest hundred. Rates are reported per 100 000 person-years. SDI categories do not sum to precisely the global total because GBD does not provide separate estimates for all locations globally and an adjustment factor is made between all estimated locations, which each have a corresponding estimated SDI value for 2017, and the global aggregate. Causes refer to overall childhood cancer unless a specific cancer type is stated. DALYs=disability-adjusted life-years. SDI=Socio-demographic Index. UI=uncertainty interval. YLDs=years of life lived with disability. YLLs=years of life lost.

* Included leukaemias not otherwise specified, chronic lymphocytic leukaemias, and chronic myeloid leukaemias.

† Cancers with less than 1000 total deaths globally in 2017.

‡ Cancers without a detailed GBD cause.

**Figure 2:**
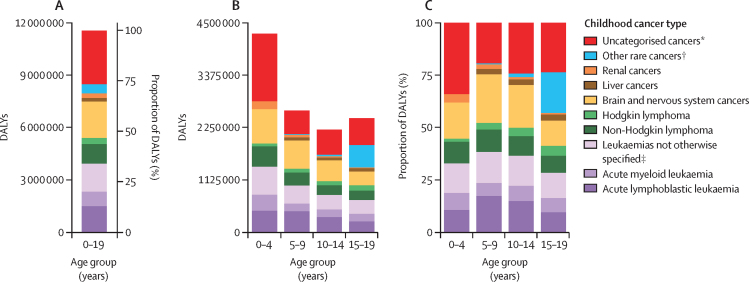
Global DALY burden of childhood cancer types, both sexes combined, 2017, in absolute and proportional burden in the 0–19 years age group (A), and absolute and proportional burden by 5-year childhood age group (B, C) DALY=disability-adjusted life-year. *Cancers without a detailed GBD cause. †Cancers with less than 1000 total deaths globally in 2017. ‡Included leukaemias not otherwise specified, chronic lymphocytic leukaemias, and chronic myeloid leukaemias.

Of the childhood cancer age groups, the 0–4-year age group had the greatest contribution to global childhood cancer DALYs (4·3 million [95% UI 3·8–4·7], or 37·0% [36·9–37·0] of the global 0–19 year childhood cancer absolute DALY burden; figure 2). Across all childhood cancer age groups, a consistently higher proportion of total DALYs was made up by YLLs (96·8% [96·8–96·8] to 98·1% [98·1–98·1] of the total age group-specific DALYs) than by YLDs (1·9% [1·9–1·9] to 3·2% [3·2–3·2] of the total age group-specific DALYs; appendix p 68). Leukaemias constituted the highest proportion of categorised childhood cancer DALY burden globally, followed by brain and nervous system cancers, with 34·1% (34·0–34·1) of all childhood cancer DALYs globally attributable to leukaemias and 18·1% (18·1–18·1) attributable to brain and nervous system cancers. These two cancer types contributed to the greatest proportional categorised DALY burden globally in all childhood age groups, except for adolescents (15–19 years). In adolescents, other rare cancers, which include cancers such as those of the testes, ovaries, and thyroid, contributed the second highest proportional DALY burden categorised (19·5% [19·4–19·5]). There was a substantial proportion of uncategorised cancers, those neoplasms without a specific cancer type noted in the current GBD data structure, throughout the childhood and adolescent age range, representing 26·5% (26·5–26·5) of all childhood cancers globally. The proportion of uncategorised cancers was highest in the 0–4-year age group (34·0% [33·9–34·0]), some of which might be attributable to cancers such as retinoblastoma and neuroblastoma, which are not currently separately estimated.

**Figure 3:**
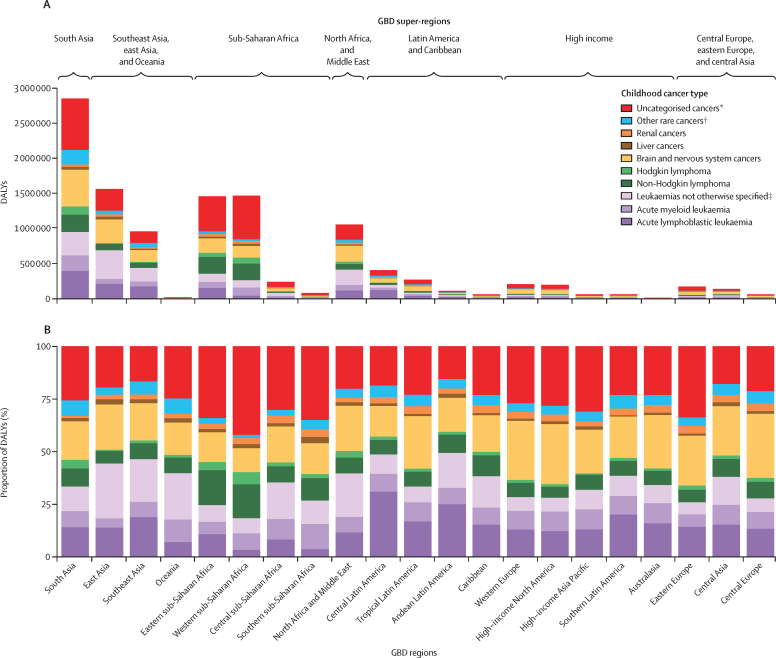
The absolute (A) and proportional (B) DALYs due to childhood (0–19 years) cancer types by GBD world region, both sexes combined, 2017 See the appendix for definitions of GBD world super-regions (p 54) and regions (p 60). DALY=disability-adjusted life-year. GBD=Global Burden of Diseases, Injuries, and Risk Factors Study. *Cancers without a detailed GBD cause. †Cancers with less than 1000 total deaths globally in 2017. ‡Included leukaemias not otherwise specified, chronic lymphocytic leukaemias, and chronic myeloid leukaemias.

When assessed by GBD world region (figure 3), there was substantial variability in the absolute and proportional DALY burden of childhood cancers by cancer type. Estimates of the proportion of childhood cancer DALYs comprised by leukaemias and brain and nervous system cancers, the most common childhood cancer types in many high-resource settings, both varied by up to 2·7 times between world regions. The greatest proportional burden of leukaemias was in Andean Latin America (49·4% [95% UI 49·1–49·7] of all childhood cancers) and central Latin America (48·7% [48·6–48·9] of all childhood cancers), whereas the greatest absolute burden rested in south Asia (954 000 [805 000–1 119 000] DALYs) and east Asia (695 000 [580 000–763 000] DALYs). Non-Hodgkin lymphomas, which include subtypes such as Burkitt's lymphomas that are not separately estimated in the GBD study, varied by approximately three times between world regions, with the greatest proportional childhood cancer DALY burden in eastern sub-Saharan Africa (16·5% [16·5–16·6] of all childhood cancers) and western sub-Saharan Africa (16·1% [16·0–16·1] of all childhood cancers). The proportion of childhood cancers that were uncategorised was prominent in all world regions, but disproportionately high in regions within sub-Saharan Africa (eg, 42·0% [41·9–42·1] in western sub-Saharan Africa).

**Figure 4:**
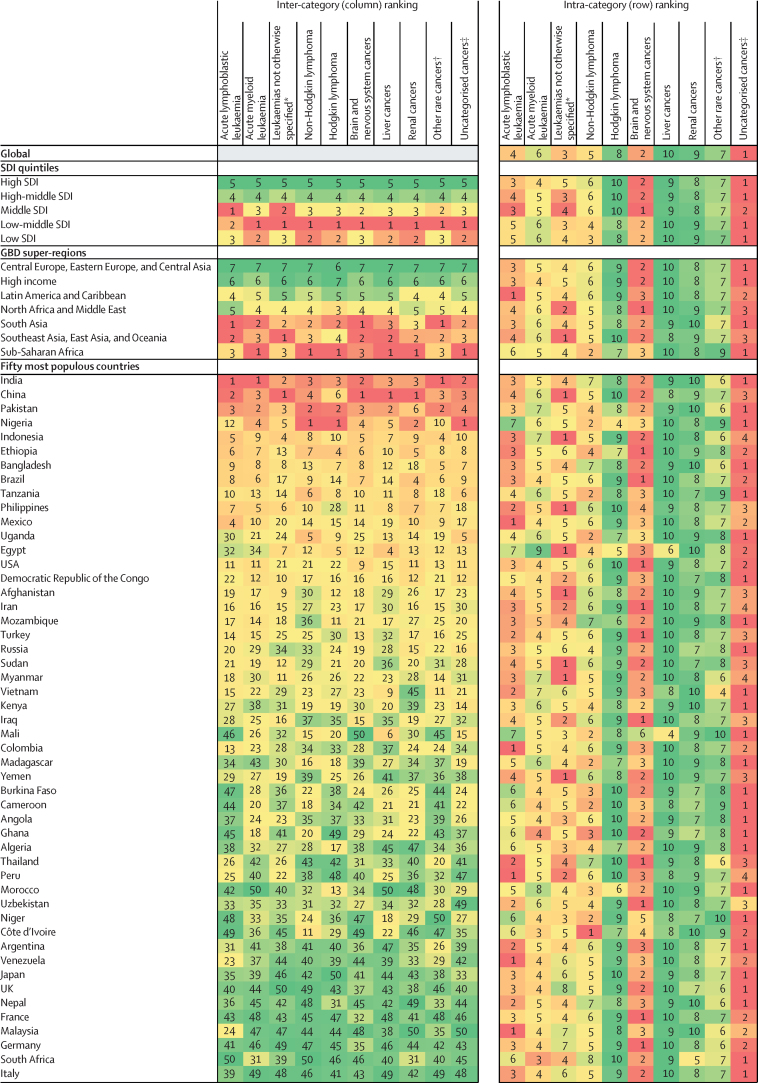
Childhood cancers ranked by number of DALYs for both sexes combined, 2017 Inter-category ranking refers to ranking vertically (ranking between the SDI quintiles, between the GBD super-regions, and between countries). Intra-category ranking refers to ranking horizontally (ranking within each SDI quintile, within each GBD super-region, and within each country). Colour intensity is proportional to absolute DALYs within the category of ranking (within the column or row). Number ranking is assigned by total absolute DALYs, with 1 representing the highest rank and greatest absolute DALY burden. For definition of GBD world superregions see the appendix (p 54). The high-income GBD super-region includes the GBD regions of Australasia, high-income Asia Pacific, high-income North America, western Europe, and southern Latin America. SDI quintiles are ordered from high to low SDI quintile, and GBD super regions are alphabetically ordered. Country order selected by total absolute DALYs; countries with the greatest total absolute DALYs, of the fifty most populous countries in the world, are listed first. The most populous countries are defined by total childhood (ages 0–19 years) population. DALY=disability-adjusted life-year. GBD=Global Burden of Diseases, Injuries, and Risk Factors Study. SDI=Sociodemographic Index. *Included leukaemias not otherwise specified, chronic lymphocytic leukaemias, and chronic myeloid leukaemias. †Cancers with less than 1000 total deaths globally in 2017. ‡Cancers without a detailed GBD cause.

Rankings of the relative burden of childhood cancers are shown in figure 4, expressed in absolute DALYs by SDI quintile, GBD super-region, and the 50 most populous countries for children in 2017. The inter-category rankings show that the low-middle SDI quintile had the greatest DALY burden for the majority of childhood cancer types, and the low SDI quintile had the most childhood cancer types that ranked second in DALY burden. Although four of the five countries with the highest childhood cancer DALYs were in the GBD super-regions (1) south Asia and (2) southeast Asia, east Asia, and Oceania, sub-Saharan Africa had the greatest DALY burden for more childhood cancer types than any other super-region. The intra-category rankings highlight that for most countries, GBD super-regions, and SDI settings, uncategorised cancers had the highest estimated DALY burden of all the childhood cancer types.

**Figure 5:**
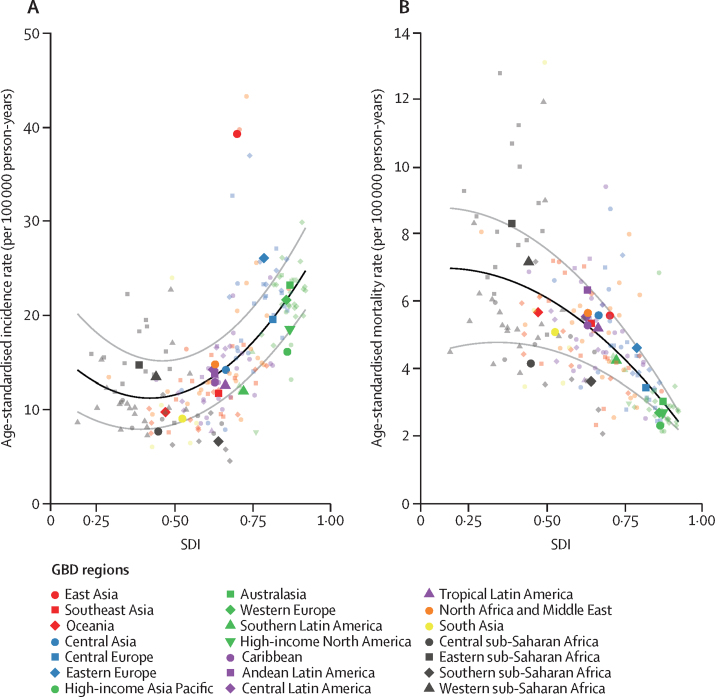
The association between SDI and childhood cancer age-standardised incidence rate (A) and mortality rate (B), 2017 Both panels represent estimates for both sexes combined. Each colour represents one of the seven GBD super-regions (red represents southeast Asia, east Asia, and Oceania; blue represents central Europe, eastern Europe, and central Asia; green represents high-income; purple represents Latin America and the Caribbean; orange represents North Africa and the Middle East; yellow represents south Asia; and grey represents sub-Saharan Africa). GBD region point estimates are median overall childhood cancer incidence or mortality rates due to inter-region variability. Lighter-coloured point estimates without labels in the legend represent countries. Country estimates are mean overall childhood cancer incidence or mortality rates. The black lines represent locally weighted smoothing based on country-level data, and the grey lines represent locally weighted smoothing of country-level 95% uncertainty intervals. See the appendix for definitions of GBD world super-regions (p 54) and regions (p 60). GBD=Global Burden of Diseases, Injuries, and Risk Factors Study. SDI=Socio-demographic Index.

Focusing on the representation of childhood cancer burden in terms of DALYs is not meant to devalue the importance of more standard cancer burden metrics. The absolute incidence and mortality values and age-standardised rates for childhood cancers globally in 2017 are presented in the table, and the relationship between country-level age-standardised childhood cancer incidence or mortality rates and SDI are shown in figure 5. With increasing SDI, age-standardised childhood cancer incidence rates generally increased, and age-standardised childhood cancer mortality rates decreased (figure 5).

**Figure 6:**
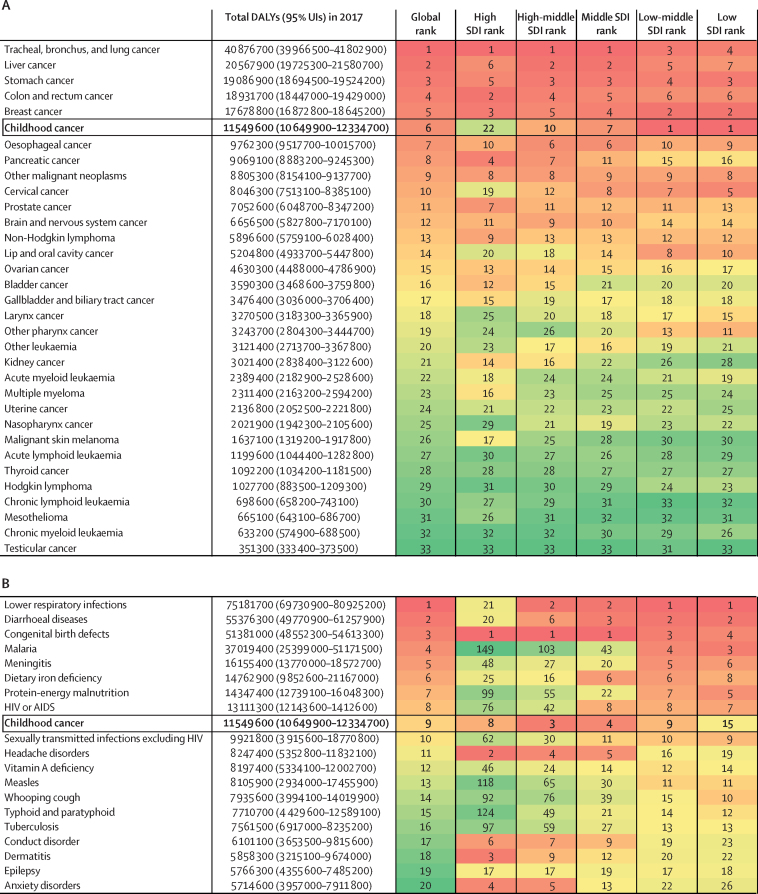
Contribution of childhood cancer to global cancer (A) and child health (B) DALY burden, both sexes combined, 2017 Disease rank assigned by total absolute DALYs globally in 2017. Childhood cancer burden is represented by the total DALYs for population aged 0–19 years. Adult cancer burden is represented by the total DALYs for each cancer subtype for the population aged 20 years and older. Total DALYs are rounded to the nearest hundred. Colour intensity is proportional to rank number. (A) All cancer causes are included. (B) Top 20 global causes of absolute DALY burden in children aged 0–19 years; childhood diseases excluded injuries and perinatal diseases. DALY=disability-adjusted life-year. SDI=Socio-demographic Index. UI=uncertainty interval.

Although the absolute incidence and mortality attributed to childhood cancers numbered in the hundreds of thousands globally, the burden as represented by YLLs and DALYs was substantially greater, in the millions globally (table). Compared with cancers of adulthood (figure 6A), childhood cancers collectively ranked first in terms of DALY contribution in low and low-middle SDI countries, higher than the DALY burden attributable to any single adult cancer type. In higher SDI settings, the burden and ranking of individual adult cancers increased, and the ranking of childhood cancer DALYs congruently decreased. Globally, childhood cancer ranked sixth in terms of DALY burden, with a DALY burden lower only than the burden attributable to cancers of the lung, liver, stomach, colon, and breast. This ranking pattern was different when childhood cancers were compared with other diseases of childhood (figure 6B), in which the highest childhood cancer DALY burden ranking was in high-middle and middle SDI settings—countries that generally have transitioning development status—rather than the lowest SDI settings. Compared with other diseases of childhood, childhood cancer ranked ninth globally in terms of DALY burden, lower than the global burden of lower respiratory infections, diarrhoeal diseases, malaria, and HIV or AIDS, but higher than the global burden of measles, typhoid, and tuberculosis.

## Discussion

To our knowledge, this paper is the first analysis to quantify the global burden of childhood cancer using DALYs. A standard global health metric routinely applied in health policy decision making, DALYs provide a more comprehensive, lifelong perspective to quantifying childhood cancer burden than has been reported in the past. Previous approaches to reporting the global burden of childhood cancers have focused on incidence, mortality, and survival; each of these metrics, although essential, provide a limited assessment when reviewed individually.^6^, ^7^, ^12^ DALYs can provide a useful summary measure of early mortality and treatment-related morbidity, especially for the childhood cancer population, in which early deaths contribute many YLLs to DALYs and in which children surviving cancer treatment often live for many years with chronic disability. In our analysis of GBD 2017, we report that although the absolute numbers of global childhood cancer incident cases and deaths were relatively small, the global burden of childhood cancer as represented in DALYs was substantial. The majority of these childhood cancer DALYs affected countries with a lower SDI, probably due to both the younger population structure observed in lower-income settings as well as a disproportionately large YLL burden, reflective of the lower survival rates observed in countries with frail health systems.

As expected, lower SDI settings were noted to have the highest age-standardised overall childhood cancer mortality rates. However, the association between childhood cancer incidence rates and SDI represented in figure 5A is unexpected, given that there are few established environmental risk factors for the majority of childhood cancers and current evidence suggests that pathological germline cancer predisposition mutations affect less than 10% of the childhood cancer population.^8^, ^20^ The cause of the trend between incidence and SDI is unknown but probably multifactorial. Although there is heterogeneity in environmental exposures between world regions and much to learn regarding potential genetic variability between populations, these factors alone are unlikely to explain the estimated variation in childhood cancer incidence by SDI. Limitations in access to health care and diagnostic capacity for children with cancer have been suggested to contribute to artificially low case ascertainment in resource-limited settings.^21^ Missed diagnoses caused by poor access to health facilities, misdiagnoses as non-oncological diseases, and under-registration due to overburdened cancer registration systems all probably contribute to this phenomenon. The GBD 2017 results highlight that improving the accuracy of global childhood cancer burden assessment will require not only expanding the quantity and quality of population-based cancer registration systems, but also increasing access to health care with the capacity to identify children with cancer regardless of where they live.

Treatment of childhood cancer in LMIC settings has been shown to be very cost-effective according to WHO–Choosing Interventions that are Cost-Effective criteria, but because of finite resources and competing health priorities in many LMIC settings, an accurate appraisal of childhood cancer disease burden using comparable metrics is essential for health policy decision making.^22^, ^23^ As low SDI countries develop, the burden of infectious diseases tends to decline and thus the relative burden of non-communicable diseases, including cancers, tends to rise—a phenomenon known as epidemiological transition. The use of DALYs provides a unique ability to contextualise the burden of childhood cancers in comparison with general diseases of childhood, and we found that childhood cancer ranks among the top five causes of DALY burden in middle and high-middle SDI settings, with a lower ranking on either end of the SDI spectrum, particularly in low SDI settings. This pattern is consistent with the epidemiological transition, with the highest childhood cancer burden relative to the burden of general diseases of childhood occurring in countries transitioning from lower to higher development status.

A different DALY pattern was observed when childhood cancers were compared with individual adult cancers— a suitable comparison for guidance of resource allocation decisions given that childhood cancers are typically treated under one clinical service, whereas adult cancers are often treated under various cancer-specialised services. Specifically, childhood cancers are the top cause of cancer burden, as expressed in DALYs, in low and low-middle SDI settings. This is a markedly different concentration of burden than occurs in adult cancers, in which DALY burden is heavily weighted towards countries with high and middle SDI status, and is probably due in part to the older population structure in higher SDI settings, as well as to lifestyle risk factors that are more prevalent in higher-resourced settings.^24^ This variation in the epidemiological patterns of cancer burden distribution in children and adults supports the view that the mechanisms of addressing cancer burden in adults, which focus on risk-reduction strategies and screening interventions, are not as relevant in the paediatric and adolescent age groups at this time. Childhood cancers generally progress rapidly, are not amenable to screening, and are fatal without swift diagnosis and treatment.^8^ Thus, improving childhood cancer outcomes will require well functioning health systems capable of early diagnosis and effective treatment.

Addressing the global burden of childhood cancer has gained greater relevance during the past 2 years since the World Health Assembly Cancer Resolution in May, 2017, and the WHO Global Initiative for Childhood Cancer announced during the High Level Meeting on non-communicable diseases at the UN General Assembly in September, 2018.^25^, ^26^ The World Health Assembly Cancer Resolution requested resource-stratified guidance for the development of cancer-control programmes, specifically calling for children and adolescents to be included in the design of these programmes. The WHO Global Initiative for Childhood Cancer is the first programme designed to address this resolution with a focus on childhood cancer and aims to increase the overall survival for six key childhood cancers (acute lymphoblastic leukaemia, Burkitt's lymphoma, Hodgkin lymphoma, low-grade glioma, retinoblastoma, and Wilms tumour) to 60% globally by 2030 through integration of childhood cancer into national cancer control policies and capacity-building interventions including the development of national centres of excellence and regional satellites.^5^ As initiatives such as these recommend countries develop and implement paediatric-specific cancer control plans over the next decade, country-specific and region-specific variations in disease burden and identification of high-yield opportunities for improvement in outcomes will be essential. In particular, evaluating the progress made in childhood cancer survival as part of the WHO Global Initiative for Childhood Cancer will be imperative to its success. The GBD study provides valuable estimates of childhood cancer epidemiology in areas where direct disease burden data are scarce or non-existent, provides the most comprehensive and contextualised global burden estimates to date through the use of DALYs, and is updated annually. Moreover, the GBD framework is already monitoring progress of the health-related UN Sustainable Development Goals.^27^, ^28^ As the WHO Global Initiative for Childhood Cancer will develop indicators similar in structure to those used for tracking of Sustainable Development Goal targets, the GBD study provides an ideal platform for monitoring global progress in childhood cancer by quantifying changes in burden and tracking proposed indicators over time.

The deeper analyses of the GBD cancer estimation process described here highlight opportunities to improve the currently applied methodology with regard to childhood cancers in particular. Inclusion of data from paediatric-specific cancer registries would add key existing information for childhood cancer incidence not currently included in the GBD data sources.^7^ However, additional data sources alone will not resolve key structural limitations in the existing GBD approach. The present anatomical site-based system of reporting cancer types functions well for adult cancers, which are primarily carcinomas, but leaves 26·5% of childhood cancer DALYs globally with a label of uncategorised cancers. Morphology is crucial to appropriate diagnosis and treatment of childhood cancers, and thus the current GBD classification system inadequately communicates the burden of childhood cancers and represents a missed opportunity for actionable burden estimates. Using the International Classification of Childhood Cancer system as a framework for reporting childhood cancers would decrease the notable proportion that are uncategorised and should be prioritised in future GBD iterations.^29^ A separate limitation in the reporting is that although GBD 2017 provided estimates for benign tumour burden in aggregate, it did not specify the portion attributable to CNS tumours. Thus, the estimates reported here do not include these tumours, which are important contributors to childhood cancer morbidity and include one of the six indicator cancers (low-grade gliomas) proposed by the WHO Global Initiative for Childhood Cancer.^25^

Furthermore, the current GBD approach to modelling the treatment and survivorship phases of childhood cancer care might lead to a systematic underestimation of YLDs and DALYs. First, the estimation of YLDs relies on data for prevalence sequelae duration from HICs. However, superimposing HIC data in this manner might not accurately represent the duration of disability seen in LMICs. This consideration is important because children in LMIC settings tend to present to care later in their disease course, potentially leading to different distributions of cancer stage at diagnosis than are observed in HICs.^3^ Addressing this issue was not historically possible because of a paucity of childhood cancer staging information in population-based cancer registries.^30^ If the recently published Toronto guidelines providing concrete staging recommendations are adopted by registries in the coming years, however, opportunities to use staging data to improve the estimation of YLDs might be possible in the near future.^30^ Second, the current GBD estimation of YLDs assumes that all children receive and complete treatment. Unfortunately, many children with cancer in LMIC settings have notable risk of therapy abandonment.^31^ Although global data on childhood cancer abandonment are limited, creating a method to account for the proportion of children who abandon therapy upfront is imperative given that untreated childhood cancer is generally fatal. Finally, the current GBD models do not incorporate the well established increased lifelong risk of multimorbidity and early death observed in childhood cancer survivors compared with the general population.^32^, ^33^, ^34^ The existing modelling of disability in childhood cancer survivors is limited to 10 years after cancer diagnosis, with children surviving past 10 years presumed to have the same risk of morbidity and mortality as the general population. Substantial data have shown this assumption to be inaccurate, and incorporation of survivorship cohort data would improve the GBD estimation of childhood cancer survivor burden.^33^, ^34^

These limitations suggest that the GBD 2017 estimates probably underestimate the DALYs associated with childhood cancer. Addressing these limitations in future GBD iterations would improve childhood cancer burden estimates and provide a better evidence base for policy, financial, and clinical decision making. Opportunities to improve on the current GBD methodology are both feasible and necessary to provide the most useful information to global health stakeholders interested in reducing disparities in global childhood cancer outcomes.

In summary, this analysis of the global burden of childhood cancer produced by the GBD 2017 study demonstrates substantial DALY burden, even when compared with cancers in adults and general diseases of childhood. Childhood cancer DALYs disproportionately affect countries with the fewest resources, underscoring the need for effective strategies to address the burden in these settings. These findings provide a global childhood cancer burden baseline from which to evaluate future progress and highlight that childhood cancer has a role in prioritisation frameworks that address global oncology and global child health.

**This online publication has been corrected. The corrected version first appeared at thelancet.com/oncology on August 6, 2019.**
