# Correction to *Lancet Oncol* 2019; 20: 1211–25

**DOI:** 10.1016/S1470-2045(19)30530-3

**Published:** 2019-09

*GBD 2017 Childhood Cancer Collaborators. The global burden of childhood and adolescent cancer in 2017: an analysis of the Global Burden of Disease Study 2017.* Lancet Oncol *2019; **20:** 1211–25*—In figure 4 of this Article, the order of countries should be by absolute disability-adjusted life-years. The following sentence has been added to the figure 4 caption: “SDI quintiles are ordered from high to low SDI quintile, and GBD super-regions are alphabetically ordered.” The next sentence should read: “Country order selected by total absolute DALYs; countries with the greatest total absolute DALYs, of the fifty most populous countries in the world, are listed first.” Amendments have been made to the affiliations list at the end of the Article. This correction has been made to the online version as of Aug 6, 2019.

