# Correction to *Lancet Oncol* 2019; 20: 1211–25

**DOI:** 10.1016/S1470-2045(21)00413-7

**Published:** 2021-08

*GBD 2017 Childhood Cancer Collaborators. The global burden of childhood and adolescent cancer in 2017: an analysis of the Global Burden of Disease Study 2017.* Lancet Oncol *2019; **20:** 1211–25*—In this Article, the order of authors in the GBD 2017 Childhood Cancer Collaborators list has been corrected. This correction has been made to the online version as of July 30, 2021.

