# Supplementary material for: The global burden of childhood and adolescent cancer in 2017: an analysis of the Global Burden of Disease Study 2017
Source: Lancet Oncol. 2019 Sep;20(9):1211–25. doi: 10.1016/S1470-2045(19)30339-0 (PMC6722045; doi:10.1016/S1470-2045(19)30339-0)
Supplement: Supplementary appendix [file mmc1.pdf]

THE LANCET
Oncology

Supplementary appendix

This appendix formed part of the original submission and has been peer reviewed. We post it as supplied by the authors.

Supplement to: GBD 2017 Childhood Cancer Collaborators. The global burden of childhood and adolescent cancer in 2017: an analysis of the Global Burden of Disease Study 2017. *Lancet Oncol* 2019; published online July 29. http://dx.doi.org/10.1016/S1470-2045(19)30339-0.

Supplementary Content

The global burden of childhood and adolescent cancer in 2017: an analysis of the Global Burden of Disease 2017 Study

Please note that portions of this supplement were copied from the supplementary content to the most recent GBD cancer publications:

Global Burden of Disease Cancer Collaboration. Global, Regional, and National Cancer Incidence, Mortality, Years of Life Lost, Years Lived With Disability, and Disability-Adjusted Life-years for 32 Cancer Groups, 1990 to 2015: A Systematic Analysis for the Global Burden of Disease Study. *JAMA Oncol* 2017; **3**(4): 524-48.[1]

and

Global Burden of Disease Cancer Collaboration. Global, Regional, and National Cancer Incidence, Mortality, Years of Life Lost, Years Lived With Disability, and Disability-Adjusted Life-Years for 29 Cancer Groups, 1990 to 2016: A Systematic Analysis for the Global Burden of Disease Study. *JAMA Oncol* 2018; **4**(11): 1553-68.[2]

Table of Contents 2

The Global Burden of Disease (GBD) Study 3

GATHER Guidelines Checklist 4

Definition of Indicator 6

GBD Cancer Estimation Process 7
Appendix Figure 1: Flowchart of GBD 2017 cancer mortality and YLL Estimation 7
Appendix Figure 2: Flowchart of GBD 2017 cancer incidence, prevalence and YLD Estimation 8

Data Sources 9
Cancer incidence data sources 9
Cancer mortality data sources 9
Cancer mortality-to-incidence ratio data sources 9
Bias of categories of input data 9
Appendix Figure 3: Global map of number of site-years of childhood cancer (ages 0-19 years) data used in the GBD 2017 study, from 1980-2017 10

Cancer types estimated in the GBD 2017 study 10
ICD cancer codes mapped to GBD 2017 cancer causes 10
Appendix Table 1: GBD 2017 cancer causes mapped to childhood cancer types 11

Data Analysis 12
Cancer registry data formatting 12
Mortality-to-incidence ratio estimation 13
Appendix Table 2: Restrictions on age and sex by each cancer type included in the cancer estimates for GBD 2017 15
CODEm models 16
Appendix Table 3: GBD 2017 covariates and level of covariates used in cause of death modeling for cancer types estimated 16
CoDCorrect 34
Incidence estimation 34
Prevalence and YLD estimation 34
Appendix Table 4: Duration of four prevalence sequelae by cancer 35
Appendix Table 5: Procedure codes used to estimate cancer procedure proportions 37
Appendix Table 6: Lay description of cancer states and corresponding disability weights 39
Uncertainty Estimation 39

Additional GBD 2017 study Methodology 40
GBD 2017 ST-GPR Methodology Overview 40
GBD 2017 CODEm Methodology Overview 45
Socio-demographic Index (SDI) definition and calculation 47

Additional Methodology Tables and Figures 48
Appendix Table 7: SDI quintile for Countries Estimated in GBD 2017 48
Appendix Figure 4: Map of GBD world super regions, 2017 54
Appendix Table 8: Definition of GBD world super regions, 2017 54
Appendix Figure 5: Map of GBD world regions, 2017 60
Appendix Table 9: Definition of GBD world regions, 2017 60

Additional Results in Tables and Figures 66
Appendix Table 10: Adult Cancer DALY Burden by SDI quintile, GBD 2017 66
Appendix Figure 6: Global Map of Ratio of Childhood to Adult Age-Standardized DALY Rates of All Combined Malignant Cancers for Both Sexes Combined in 2017 67
Appendix Table 11: Total Global YLL, YLD, and DALY Burden by 5-Year Childhood Age Group in 2017 68
Appendix Figure 7: Global DALY Burden by Childhood Cancer Types, Both Sexes, 2017, in (a) Absolute and (b) Proportional DALY Burden by Age Group and SDI Quintile 69
Appendix Figure 8: The Relationship between Socio-demographic Index (SDI) and Childhood Cancer DALY Rates in 2017 71

References 72

The Global Burden of Disease (GBD) study

In response to conflicting disease burden estimates in the 1990s, the Global Burden of Disease (GBD) study was created in an effort to establish comprehensive and comparable health metrics. With this goal in mind, a key principle in the GBD approach to estimation of disease burden is that an individual can have only one cause of death (avoiding the issue of counting an individual death multiple times if that individual had multiple diseases, while recognising that this may underestimate disease burden due to intermediate causes of death). In addition to reporting estimates of mortality and years of life lost (YLLs) for over 300 diseases and injuries, the GBD study also quantifies non-fatal components of disease including years lived with disability (YLDs) and disability-adjusted life-years (DALYs), a metric that represents a combination of both the fatal and non-fatal components of disease. The GBD approach uses all relevant data sources, rather than a single type of data. Finally, as there is continual methodological refinement with each GBD iteration, the results in each successive iteration supersede the results of prior GBD studies for the entire newly estimated time series. A protocol for the GBD study can be found online at http://www.healthdata.org/sites/default/files/files/Projects/GBD/GBD_Protocol.pdf.

GATHER Guidelines Checklist

**Objectives and Funding**	**Reported in the Manuscript and Appendix**
1. Define the indicator(s), populations (including age, sex, and geographic entities), and time period(s) for which estimates were made.	Appendix: "Definition of indicator"
2. List the funding sources for the work.	See main manuscript
**Data Inputs**	
**For all data inputs from multiple sources that are synthesized as part of the study:**	
3. Describe how the data were identified and how the data were accessed.	Appendix: "Data sources"
4. Specify the inclusion and exclusion criteria. Identify all ad-hoc exclusions.	Appendix: "Data sources"
5. Provide information about all included data sources and their main characteristics. For each data source used, report reference information or contact name/institution, population represented, data collection method, year(s) of data collection, sex and age range, diagnostic criteria or measurement method, and sample size, as relevant.	http://ghdx.healthdata.org/gbd-2017
6. Identify and describe any categories of input data that have potentially important biases (e.g., based on characteristics listed in item 5).	Appendix: "Bias of categories of input data"
**For data inputs that contribute to the analysis but were not synthesized as part of the study:**	
7. Describe and give sources for any other data inputs.	http://ghdx.healthdata.org/gbd-2017
**For all data inputs:**	
8. Provide all data inputs in a file format from which data can be efficiently extracted (e.g., a spreadsheet rather than a PDF), including all relevant meta-data listed in item 5. For any data inputs that cannot be shared because of ethical or legal reasons, such as third-party ownership, provide a contact name or the name of the institution that retains the right to the data.	http://ghdx.healthdata.org/gbd-2017
**DATA ANALYSIS**	
9. Provide a conceptual overview of the data analysis method. A diagram may be helpful.	• Appendix Figure 1: Flowchart GBD cancer mortality, YLL estimation<br>• Appendix Figure 2: Flowchart GBD cancer incidence, prevalence, YLD estimation
10. Provide a detailed description of all steps of the analysis, including mathematical formulae. This description should cover, as relevant, data cleaning, data pre-processing, data adjustments and weighting of data sources, and mathematical or statistical model(s).	Appendix: "Data Analysis"
11. Describe how candidate models were evaluated and how the final model(s) were selected.	CODEm models[3]; see Appendix Table 3: GBD 2017 covariates and level of

	covariates used in cause of death modelling for cancer types estimated
12. Provide the results of an evaluation of model performance, if done, as well as the results of any relevant sensitivity analysis.	See SR Figure 5 on p 20 of Supplement 2 to "Global, regional, and national age-sex-specific mortality for 282 causes of death in 195 countries and territories, 1980-2017: a systematic analysis for the Global Burden of Disease Study 2017"[4]
13. Describe methods of calculating uncertainty of the estimates. State which sources of uncertainty were, and were not, accounted for in the uncertainty analysis.	Appendix: "Data Analysis"
14. State how analytic or statistical source code used to generate estimates can be accessed.	http://ghdx.healthdata.org/gbd-2017/code
**RESULTS AND DISCUSSION**	
15. Provide published estimates in a file format from which data can be efficiently extracted.	GBD 2017 estimates are available online (http://vizhub.healthdata.org/gbd-compare and http://ghdx.healthdata.org/gbd-results-tool)
16. Report a quantitative measure of the uncertainty of the estimates (e.g., uncertainty intervals).	See main manuscript "Results"
17. Interpret results in light of existing evidence. If updating a previous set of estimates, describe the reasons for changes in estimates.	See main manuscript "Discussion"
18. Discuss limitations of the estimates. Include a discussion of any modelling assumptions or data limitations that affect interpretation of the estimates.	See main manuscript "Discussion"

Definition of Indicator

In this publication, estimates for ten cancer groups, for both sexes, for the year 2017 and for the five-year GBD age groups (0-4, 5-9, 10-14, and 15-19) included within "childhood cancers" are presented globally and for regions which include 195 countries or territories.

All ICD9 codes pertaining to cancer (140-209) and ICD10 codes (C00-C96) except for Kaposi sarcoma (ICD10: C46) and non-melanoma skin cancer (ICD10: C44) are being included in these estimates. For a complete list of ICD codes and their respective GBD causes, refer to "Appendix Table 4: List of International Classification of Diseases (ICD) codes mapped to the Global Burden of Disease cause list for causes of death" on page 400 of Supplement 1 to "Global, regional, and national age-sex-specific mortality for 282 causes of death in 195 countries and territories, 1980–2017: a systematic analysis for the Global Burden of Disease Study 2017"[4] and "Appendix Table 4: List of International Classification of Diseases (ICD) codes mapped to the Global Burden of Disease cause list" on page 1015 of Supplement 1 to "Global, regional, and national incidence, prevalence, and years lived with disability for 354 diseases and injuries for 195 countries and territories, 1990–2017: a systematic analysis for the Global Burden of Disease Study 2017".[5] Appendix Table 1 demonstrates the mapping from GBD cause to the childhood cancer types estimated in this manuscript.

A complete list of countries and territories estimated in GBD 2017 can be found in "Appendix Table 2: GBD 2017 location hierarchy with levels" in Supplement 1 to "Global, regional, and national age-sex-specific mortality for 282 causes of death in 195 countries and territories, 1980–2017: a systematic analysis for the Global Burden of Disease Study 2017".[4]

GBD Cancer Estimation Process

Appendix Figure 1: Flowchart of GBD 2017 cancer mortality and YLL estimation

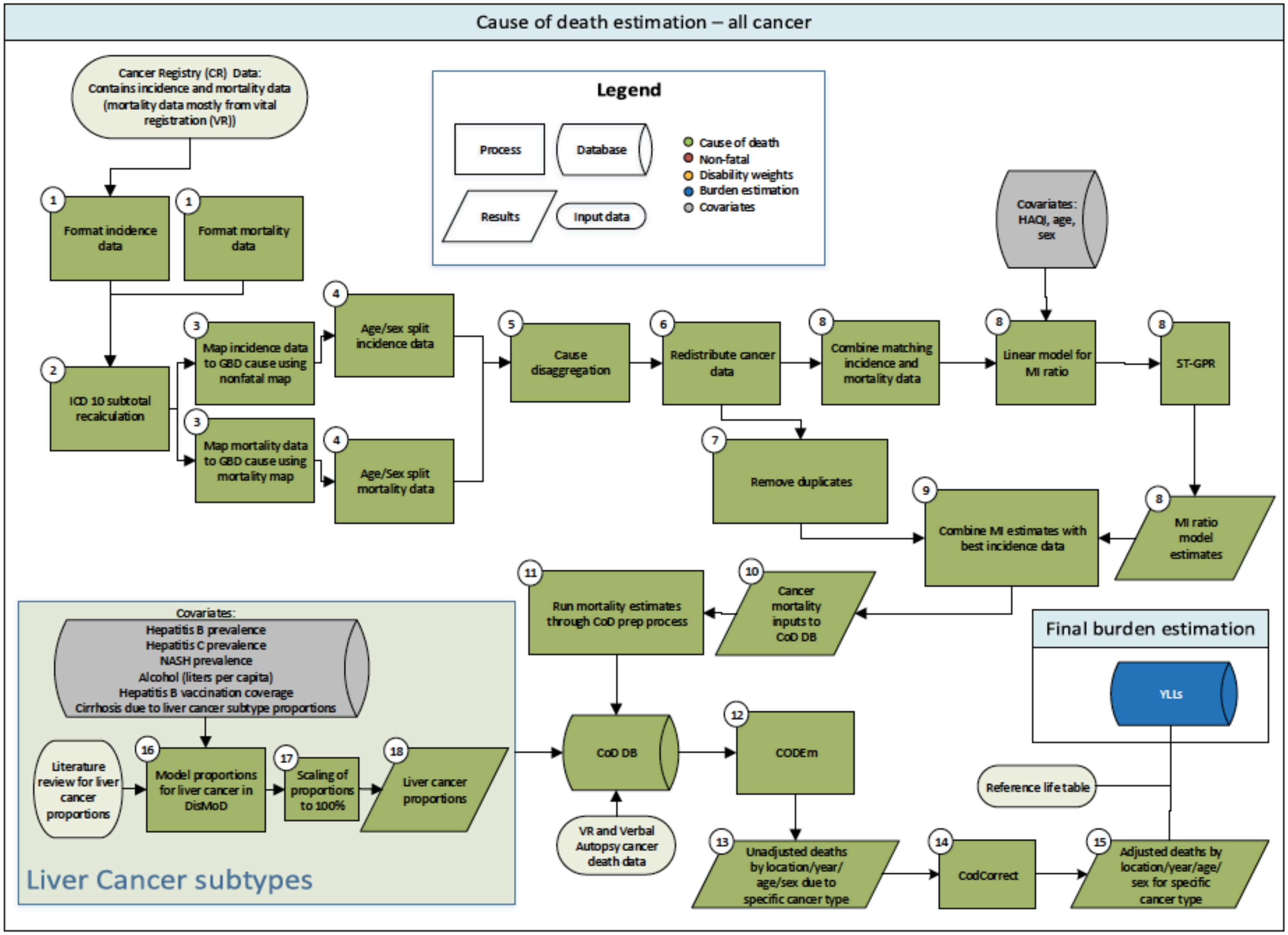

Abbreviations: ICD: International classification of diseases; DB: database, ST-GPR: Space-time smoothing, Gaussian process regression, COD: Causes of death

Appendix Figure 2: Flowchart of GBD 2017 cancer incidence, prevalence and YLD estimation

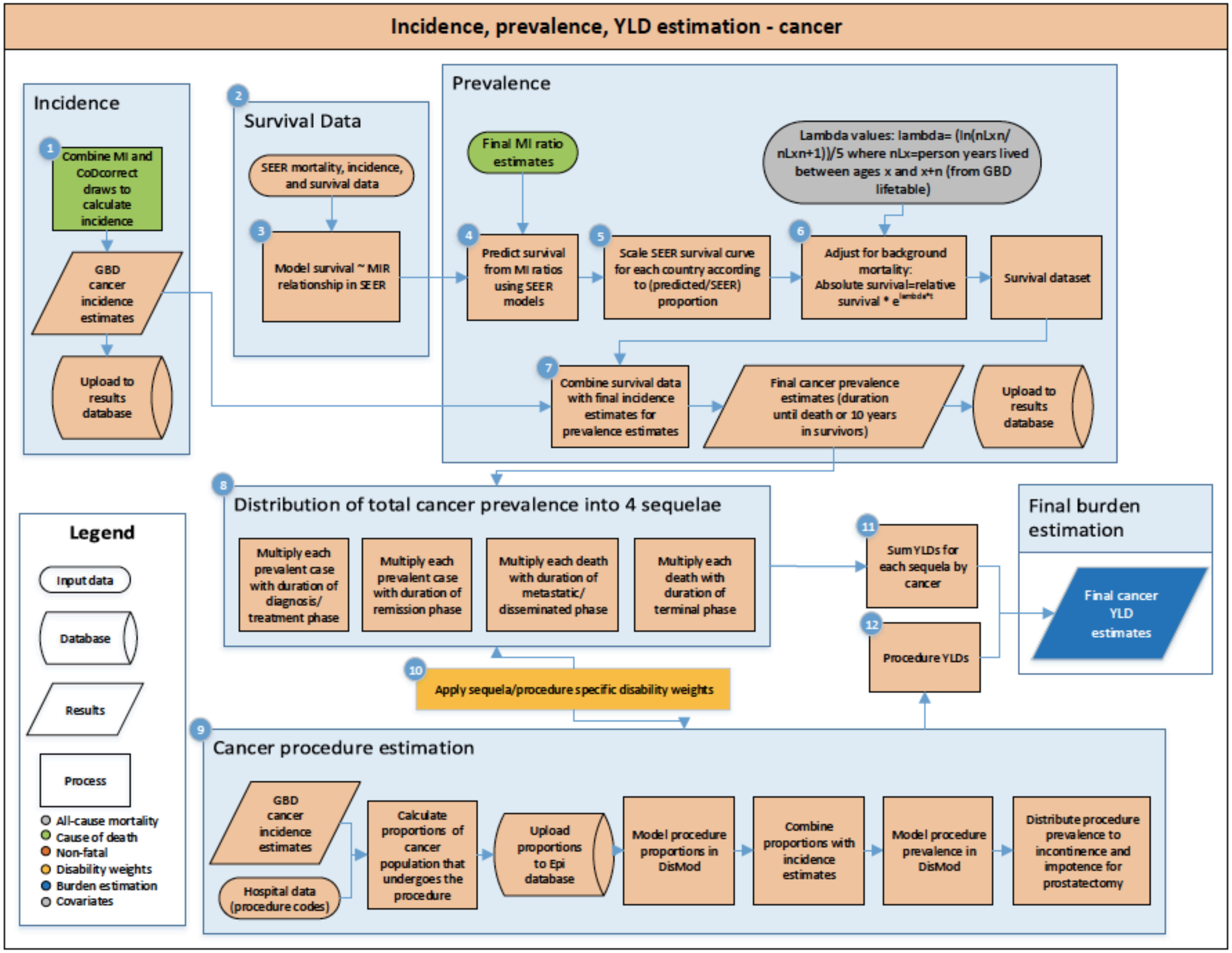

Data Sources

Cancer incidence data sources

Cancer incidence was sought from individual cancer registries and aggregated databases of cancer registry data such as "Cancer Incidence In Five Continents" (CI5)"[6-16], EUREG[17], and NORDCAN[18]. Data were excluded if they were not representative of the coverage population (ie, cancer registries needed to be population-based in order to be included), if they did not cover all malignant neoplasms as defined in ICD9 (140-208) or ICD10 (C00-C96) (eg, specialty or disease-based cancer registries were excluded), if they did not include data for both sexes and all age groups, if the data were limited to years prior to 1980, or if the source did not provide details on the population covered. Preference was given to registries with national coverage over those with only local coverage, except those from countries where the GBD 2017 study provides sub-national estimates. A list of the cancer registries included in our analysis and the years covered can be found in the online GBD citation tool http://ghdx.healthdata.org/gbd-2017.

Cancer mortality data sources

A detailed description of the data sources and processing steps for the cause of death database can be found in the appendix to the GBD 2017 capstone publication "Global, regional, and national age-sex-specific mortality for 282 causes of death in 195 countries and territories, 1980–2017: a systematic analysis for the Global Burden of Disease Study 2017."[4]

Cancer mortality-to-incidence ratio data sources

Most cancer registries only report cancer incidence. However, if a cancer registry reported both cancer incidence and mortality, mortality data were also extracted from the source to be used in the mortality-to-incidence ratio estimation. In the case when high-quality mortality data were available but not reported by the cancer registry, vital registration mortality data were matched to the cancer registry's incidence data.

Bias of categories of input data

Bias of the input data included in the COD database is described elsewhere.[4] Cancer registry data can be biased in multiple ways. A high proportion of ill-defined cancer cases in the registry data require redistribution of these cases to other cancers, which introduces a potential for bias. Changes between coding systems can lead to artificial differences in disease estimates; however, we adjust for this bias by mapping the different coding systems to the GBD causes. Underreporting of cancers that require advanced diagnostic techniques (eg, leukaemias, brain and nervous system cancers, etc.) can be an issue in cancer registries from resource-limited countries. Alternatively, misclassification of metastatic sites as primary cancer can lead to overestimation of cancer sites that are common sites for metastases such as the brain or liver. Since many cancer registries are located in urban areas, the representativeness of the registry for the general population can also be problematic. The accuracy of mortality data reported in cancer registries usually depends on the quality of the local vital registration system. If the vital registration system data are incomplete or of poor quality, the mortality-to-incidence ratio may be biased to lower ratios.

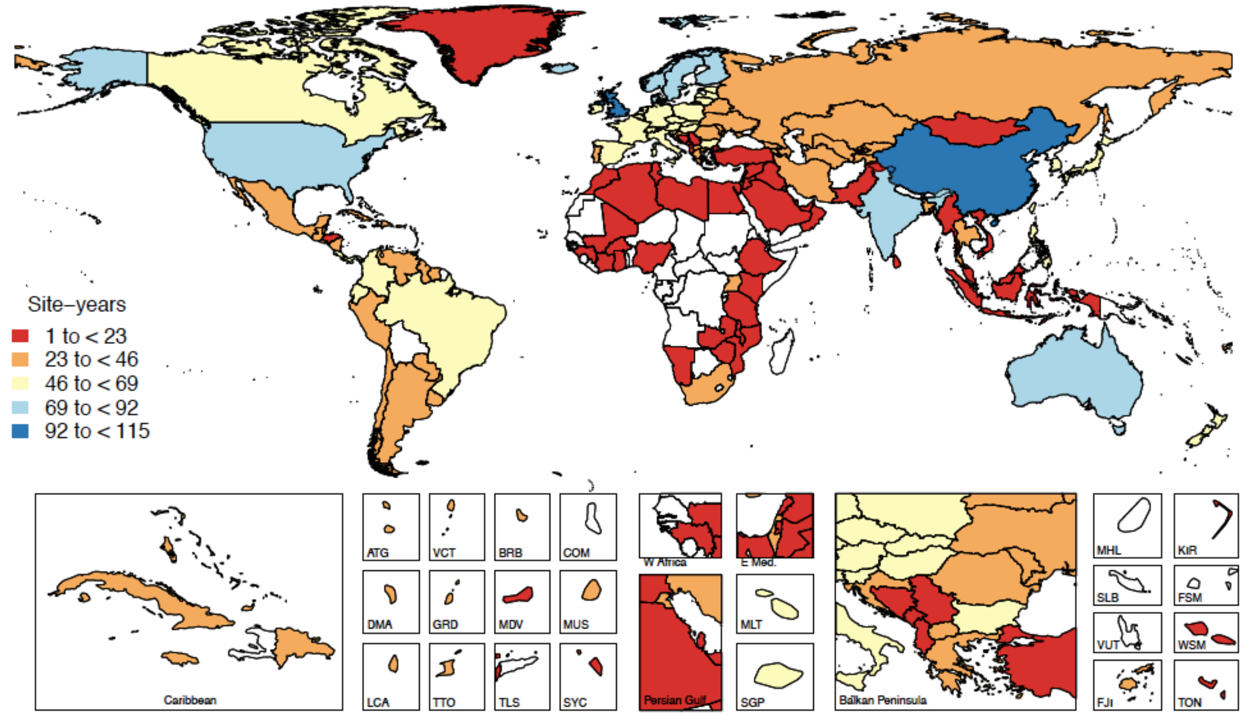

Appendix Figure 3: Global map of number of site-years of childhood cancer (ages 0-19 years) data used in the GBD 2017 study, from 1980 to 2017. Data sources included cancer registries, vital registration systems, and verbal autopsy studies. ATG: Antigua and Barbuda; VCT: Saint Vincent and the Grenadines; BRB: Barbados; COM: Comoros; W Africa: West Africa; E Med: Eastern Mediterranean; MHL: Marshall Islands; KIR: Kiribati; DMA: Dominica; GRD: Grenada; MDV: Maldives; MUS: Mauritius; MLT: Malta; SLB: Solomon Islands; FSM: Federated States of Micronesia; LCA: Saint Lucia; TTO: Trinidad and Tobago; TLS: Timor-Leste; SYC: Seychelles; SGP: Singapore; VUT: Vanuatu; WSM: Samoa; FJI: Fiji; TON: Tonga.

<u>Cancer types estimated in the GBD 2017 study</u>

ICD cancer codes mapped to GBD 2017 cancer causes

Please refer to "Appendix Table 4: List of International Classification of Diseases (ICD) codes mapped to the Global Burden of Disease cause list for causes of death" on page 400 of Supplement 1 to "Global, regional, and national age-sex-specific mortality for 282 causes of death in 195 countries and territories, 1980–2017: a systematic analysis for the Global Burden of Disease Study 2017"[4] and "Appendix Table 4: List of International Classification of Diseases (ICD) codes mapped to the Global Burden of Disease cause list" on page 1015 of Supplement 1 to "Global, regional, and national incidence, prevalence, and years lived with disability for 354 diseases and injuries for 195 countries and territories, 1990–2017: a systematic analysis for the Global Burden of Disease Study 2017".[5]

Appendix Table 1: GBD 2017 cancer causes mapped to childhood cancer types

GBD cancer cause	Childhood cancer cause
Bladder cancer	Other rare cancers
Brain and nervous system cancer	Brain & nervous system cancers
Breast cancer	Other rare cancers
Cervical cancer	Other rare cancers
Colon and rectum cancer	Other rare cancers
Oesophageal cancer	Other rare cancers
Gallbladder and biliary tract cancer	Other rare cancers
Hodgkin lymphoma	HL
Kidney cancer	Renal cancers
Larynx cancer	Other rare cancers
Acute lymphoid leukaemia	ALL
Chronic lymphoid leukaemia	Leukaemias NOS
Acute myeloid leukaemia	AML
Chronic myeloid leukaemia	Leukaemias NOS
Other leukaemia	Leukaemias NOS
Liver cancer	Liver cancers
Tracheal, bronchus, and lung cancer	Other rare cancers
Non-Hodgkin lymphoma	NHL
Malignant skin melanoma	Other rare cancers
Mesothelioma	Other rare cancers
Lip and oral cavity cancer	Other rare cancers
Multiple myeloma	Other rare cancers
Nasopharynx cancer	Other rare cancers
Other malignant neoplasms	Uncategorised cancers
Other pharynx cancer	Other rare cancers
Ovarian cancer	Other rare cancers
Pancreatic cancer	Other rare cancers
Prostate cancer	Other rare cancers
Stomach cancer	Other rare cancers
Testicular cancer	Other rare cancers
Thyroid cancer	Other rare cancers
Uterine cancer	Other rare cancers

ALL=Acute lymphoblastic leukaemia; AML=acute myeloid leukaemia; Leukaemias NOS=leukaemias not otherwise specified, chronic lymphocytic leukaemias (CLL) or chronic myeloid leukaemias (CML). NHL=non-Hodgkin lymphomas; HL=Hodgkin lymphomas; Other rare cancers=cancers with < 1000 total deaths globally in 2017; Uncategorised cancers=cancers without a detailed GBD cause.

Data Analysis

Cancer registry data formatting

Cancer registry data went through multiple processing steps before integration with the COD database. First, the original data were transformed into standardised files, which included standardisation of format, categorisation, and registry names (#1 in "Flowchart of GBD 2017 cancer mortality and YLL estimation"). Second, some cancer registries report individual codes as well as aggregated totals (eg, C18, C19, and C20 are reported individually, but the aggregated group of C18-C20 [colorectal cancer] is also reported in the registry data). The data processing step "subtotal recalculation" (#2 in "Flowchart of GBD 2017 cancer mortality and YLL estimation") verifies these totals and subtracts the values of any individual codes from the aggregates.

In the third step (#3 in "Flowchart of GBD 2017 cancer mortality and YLL estimation"), cancer registry incidence data and cancer registry mortality data are mapped to GBD causes. A different map is used for incidence and for mortality data because of the assumption that there are no deaths for certain cancers. Examples are benign or in situ neoplasms. Benign or in situ neoplasms found in the cancer registry incidence dataset were simply dropped from that dataset. The same neoplasms reported in a cancer registry mortality dataset were mapped to the respective invasive cancer (eg, melanoma in situ in the cancer registry incidence dataset was dropped from the dataset; melanoma in situ in the cancer registry mortality dataset was mapped to melanoma).

In the fourth data processing step (#4 in "Flowchart of GBD 2017 cancer mortality and YLL estimation") cancer registry data were standardised to the GBD age groups. Age-specific incidence rates were generated using all datasets that include microdata, and datasets that report age groups up to 95+ years of age. Age-specific mortality rates were generated from the CoD data through a method described in Section 2.5 in Supplement 1 to "Global, regional, and national age-sex-specific mortality for 282 causes of death in 195 countries and territories, 1980–2017: a systematic analysis for the Global Burden of Disease Study 2017.[4] Age-specific proportions were then generated by applying the age-specific rates to a given registry population that required age-splitting to produce the expected number of cases/deaths for that registry by age. The expected number of cases/deaths for each sex, age, and cancer were then normalised to 1, creating final, age-specific proportions. These proportions were then applied to the total number of cases/deaths by sex and cancer to get the age-specific number of cases/deaths.

In the rare case that the cancer registry only contained data for both sexes combined, the age-specific cases/deaths were split and re-assigned to separate sexes using the same weights that are used for the age-splitting process. Starting from the expected number of deaths, proportions were generated by sex for each age (eg, if for ages 15-19 years old there are six expected deaths for males and four expected deaths for females, then 60% of the combined-sex deaths for ages 15-19 years would be assigned to males and the remaining 40% would be assigned to females).

In the fifth step (#5 in "Flowchart of GBD 2017 cancer mortality and YLL estimation") data for cause entries that are aggregates of GBD causes were redistributed. Examples of these aggregated causes include some registries reporting ICD10 codes C00-C14 together as, "lip, oral cavity, and pharyngeal cancer." These groups were broken down into sub-causes that could be

mapped to single GBD causes. In this example, those include lip and oral cavity cancer (C00-C08), nasopharyngeal cancer (C11), cancer of other parts of the pharynx (C09-C10, C12-C13), and "Malignant neoplasm of other and ill-defined sites in the lip, oral cavity and pharynx" (C14). To redistribute the data, weights were created using the same "rate-applied-to-population" method employed in age-sex splitting (see step four above). For the undefined code (C14 in this example) an "average all cancer" weight was used, which was generated by adding all cases from SEER/NORDCAN/CI5 and dividing those by the combined population. Then, proportions were generated by sub-cause for each aggregate cause as in the sex-splitting example above (see step four). The total number of cases from the aggregated group (C00-C14) was then recalculated for each subgroup and the undefined code (C14). C14 was then redistributed as a "garbage code" in step six. Distinct proportions were used for C44 (non-melanoma skin cancer) and C46 (Kaposi's sarcoma). C46 entries were redistributed as "other cancer," and HIV.

In the sixth step (#6 in "Flowchart of GBD 2017 cancer mortality and YLL estimation") unspecified codes ("garbage codes") were redistributed. Redistribution of cancer registry incidence and mortality data mirrored the process of the redistribution used in the cause of death database (see Section 2.7 in Supplement 1 to "Global, regional, and national age-sex-specific mortality for 282 causes of death in 195 countries and territories, 1980–2017: a systematic analysis for the Global Burden of Disease Study 2017).[4]

In the seventh step (#7 in "Flowchart of GBD 2017 cancer mortality and YLL estimation") duplicate or redundant sources were removed from the processed cancer registry dataset. Duplicate sources were present if, for example, the cancer registry was part of a CI5 dataset, but we also had data directly from the registry. Redundancies occurred and were removed as described in "Inclusion and exclusion criteria," where more detailed data were available, or when national registry data could replace regionally representative data. From here, two parallel selection processes were run to generate input data for the MIR models and to generate incidence for final mortality estimation. Higher priority was given to registry data from the most standardised source when creating the final incidence input, whereas for the MIR model input, only sources that reported incidence and mortality were used.

Mortality-to-incidence ratio estimation
In the eighth step (#8 in "Flowchart of GBD 2017 cancer mortality and YLL estimation") the processed incidence and mortality data from cancer registries were matched by cancer, age, sex, year, and location to generate MIRs. To summarise the MIRs estimation process, incidence and mortality data from cancer registries were matched by cancer, age, sex, year, and location to generate MIRs. These MIRs were used as input for a three-step modelling approach using the GBD 2017 spatiotemporal Gaussian process regression (ST-GPR) approach, with the Healthcare Access and Quality Index (HAQ Index) as a covariate in the linear step mixed effects model using a logit link function. We used a fixed effect logistic regression model to estimate MIRs[19]:

$$\text{logit}\left(MI\ ratio_{c,a,s,t}\right) = \alpha + \beta_1 HAQ\ Index_{c,t} + \sum_{a}^{A} \beta_2 I_a + \beta_3 I_s + \epsilon_{c,a,s,t}$$

c: country, a: age group, t: time (years); s: sex
HAQ Index: Healthcare Access and Quality Index
I: indicator variable
$\epsilon_{c,a,s,t}$: Gaussian error term

Information on ST-GPR is included in this appendix page 40, as well as on page 28 of Supplementary appendix 1 to “Global, regional, and national comparative risk assessment of 84 behavioural, environmental and occupational, and metabolic risks or clusters of risks for 195 countries and territories, 1990–2017: a systematic analysis for the Global Burden of Disease Study 2017”.[20] Predictions were made without the random effects. The ST-GPR model has three main hyper-parameters that control for smoothing across time, age, and geography. The time adjustment parameter (λ) was set to 2, which aims to borrow strength from neighbouring time points (ie, the exposure in this year is highly correlated with exposure in the previous year but less so further back in time). The age adjustment parameter ω was set to 0.5, which borrows strength from data in neighbouring age groups. The space adjustment parameter ξ was set to 0.95 in locations with data and to 0.5 in locations without data (the higher ξ was applied when at least one age-sex group in the country of estimation had at least five unique data points. The lower ξ was applied when estimating data-scarce countries). Zeta aims to borrow strength across the hierarchy of geographical locations. For the amplitude parameter in the Gaussian process regression we used 2, and for the scale we used a value of 15.

For each cancer, MIRs from locations in HAQ Index quintiles 1-4 were dropped if they were below the median of MIRs from locations in HAQ Index quintile 5 in order to remove unrealistic values. As low MIRs imply better survival than high MIRs, we make the assumption that countries with high HAQ Index (quintile 5) should have better cancer outcomes, on average, than countries with lower HAQ Index. Given that it is unlikely that a country in HAQ Index quintiles 1-4 would have the capacity to achieve a MIR lower than the median of HAQ Index quintile 5, these are excluded. We also dropped MIRs from locations in HAQ Index quintiles 1-4 if the MIRs were above the third quartile + 1.5 * IQR (inter-quartile range) within each quintile to account for the potential underreporting of incidence compared to mortality from locations in HAQ Index quintiles 1-4. We dropped all MIRs that were based on less than 25 cases to avoid noise due to small numbers except for mesothelioma and acute lymphoid leukaemia, where we dropped MIRs that were based on less than ten cases because of lower data availability for these two cancers. We also aggregated incidence and mortality to the youngest five-year age bin where we had at least 50 data points to avoid MIR predictions in young age groups that were based on few data points. The MIRs in the age-bin that was used to aggregate MIRs to, was used to backfill the MIRs for younger age groups.

Since MIRs can be above 1, especially in older age groups and cancers with low cure rates, we used the 95th percentile of the cleaned dataset that only included MIRs that were based on 50 or more cases, to cap the MIR input data. This “upper cap” was used to allow MIRs over 1 but to constrain the MIRs to a maximum level. To run the logit model, the input data were divided by the upper caps and model predictions after ST-GPR was rescaled by multiplying them by the upper caps. To constrain the model at the lower end, we used the 5th percentile of the cancer-specific cleaned MIR input data to replace all model predictions with this lower cap. Final MIRs were matched with the cancer registry incidence dataset in the ninth step (#9 in “Flowchart of GBD 2017 cancer mortality and YLL estimation”) to generate mortality estimates (Incidence * Mortality/Incidence = Mortality) (#10 in “Flowchart of GBD 2017 cancer mortality and YLL estimation”). The final mortality estimates were then uploaded into the COD database (#11 in “Flowchart of GBD 2017 cancer mortality and YLL estimation”). Cancer-specific mortality modelling then followed the general CODEm process (see “CODEm models” section on p16 in this Appendix).

Appendix Table 2: Restrictions on age and sex by each cancer type included in the cancer estimates for GBD 2017

Cause	Minimum age	Maximum age	Sex restrictions
Lip and oral cavity cancer	15	None	
Nasopharynx cancer	5	None	
Other pharynx cancer	15	None	
Oesophageal cancer	15	None	
Stomach cancer	15	None	
Colon and rectum cancer	15	None	
Liver cancer	5	None	
Liver cancer due to hepatitis B	5	None	
Liver cancer due to hepatitis C	5	None	
Liver cancer due to alcohol use	15	None	
Liver cancer due to NASH	15	None	
Liver cancer due to other causes	5	None	
Gallbladder and biliary tract cancer	15	None	
Pancreatic cancer	15	None	
Larynx cancer	15	None	
Tracheal, bronchus, and lung cancer	15	None	
Malignant skin melanoma	15	None	
Breast cancer	15	None	
Cervical cancer	15	None	Females Only
Uterine cancer	15	None	Females Only
Ovarian cancer	15	None	Females Only
Prostate cancer	15	None	Males Only
Testicular cancer	15	None	Males Only
Kidney cancer	None	None	
Bladder cancer	15	None	
Brain and nervous system cancer	None	None	
Thyroid cancer	10	None	
Mesothelioma	15	None	
Hodgkin lymphoma	None	None	
Non-Hodgkin lymphoma	None	None	
Multiple myeloma	15	None	
Leukaemia	None	None	
Acute lymphoid leukaemia	None	None	
Chronic lymphoid leukaemia	15	None	
Acute myeloid leukaemia	None	None	
Chronic myeloid leukaemia	15	None	
Other leukaemia	None	None	
Other malignant cancers	None	None	

Age restrictions used are identical for all metrics estimated (incidence, mortality, YLLs, YLDs, and DALYs).

CODEm models

Mortality estimates for each cancer were generated using CODEm. Methods describing the CODEm approach are included in this appendix page 45, Section 3.1 in Supplement 1 to "Global, regional, and national age-sex-specific mortality for 282 causes of death in 195 countries and territories, 1980–2017: a systematic analysis for the Global Burden of Disease Study 2017" and elsewhere.[3,4] In brief, the CODEm modelling approach is based on the principles that all types of available data should be used even if data quality varies; that individual models but also ensemble models should be tested for their predictive validity; and that the best model or sets of models should be chosen based on the out of sample predictive validity. Models were run separately for countries with extensive and complete vital registration data and countries with less VR data to prevent an inflation in the uncertainty around the estimates in "data-rich" countries. Covariates were selected based on a possible predictive relationship between the covariate and the specific cancer mortality.

Appendix Table 3: GBD 2017 covariates and level of covariates used in cause of death modelling for cancer types estimated

Cause	Sex	Covariate	Level 1	Level 2	Level 3
Oesophageal cancer	Female	Tobacco (cigarettes per capita)	X		
Oesophageal cancer	Male	Tobacco (cigarettes per capita)	X		
Oesophageal cancer	Male	Healthcare Access and Quality Index		X	
Oesophageal cancer	Female	LDI (I$ per capita)			X
Oesophageal cancer	Male	LDI (I$ per capita)			X
Oesophageal cancer	Female	Education (years per capita)			X
Oesophageal cancer	Male	Education (years per capita)			X
Oesophageal cancer	Female	Alcohol (litres per capita)	X		
Oesophageal cancer	Male	Alcohol (litres per capita)	X		
Oesophageal cancer	Male	Mean BMI	X		
Oesophageal cancer	Female	Mean BMI	X		
Oesophageal cancer	Male	Socio-demographic Index		X	
Oesophageal cancer	Female	Socio-demographic Index			X
Oesophageal cancer	Female	Socio-demographic Index		X	
Oesophageal cancer	Male	Indoor air pollution (all cooking fuels)		X	
Oesophageal cancer	Female	Indoor air pollution (all cooking fuels)		X	
Oesophageal cancer	Female	Healthcare Access and Quality Index		X	
Oesophageal cancer	Female	Smoking prevalence	X		
Oesophageal cancer	Male	Smoking prevalence	X		
Oesophageal cancer	Female	Tobacco (cigarettes per capita)	X		
Oesophageal cancer	Male	Log-transformed age-standardised SEV scalar: Esophag C	X		
Oesophageal cancer	Female	Log-transformed age-standardised SEV scalar: Esophag C	X		
Oesophageal cancer	Male	Tobacco (cigarettes per capita)	X		

Oesophageal cancer	Female	Improved water source (proportion with access)		X	
Oesophageal cancer	Male	Log-transformed SEV scalar: Esophag C	X		
Oesophageal cancer	Female	Sanitation (proportion with access)		X	
Oesophageal cancer	Male	Sanitation (proportion with access)		X	
Oesophageal cancer	Male	Vegetables adjusted (g)		X	
Oesophageal cancer	Female	Vegetables adjusted (g)		X	
Oesophageal cancer	Male	Fruits adjusted (g)		X	
Oesophageal cancer	Female	Fruits adjusted (g)		X	
Oesophageal cancer	Male	Improved water source (proportion with access)		X	
Stomach cancer	Female	LDI (I$ per capita)			X
Stomach cancer	Male	Cumulative cigarettes (10 years)	X		
Stomach cancer	Female	Vegetables adjusted (g)		X	
Stomach cancer	Female	Education (years per capita)			X
Stomach cancer	Male	LDI (I$ per capita)			X
Stomach cancer	Male	Healthcare Access and Quality Index		X	
Stomach cancer	Female	Smoking prevalence	X		
Stomach cancer	Male	Mean BMI		X	
Stomach cancer	Female	Mean BMI		X	
Stomach cancer	Male	Socio-demographic Index			X
Stomach cancer	Female	Socio-demographic Index			X
Stomach cancer	Male	Smoking prevalence	X		
Stomach cancer	Male	Fruits adjusted (g)		X	
Stomach cancer	Female	Healthcare Access and Quality Index		X	
Stomach cancer	Female	Cumulative cigarettes (10 years)	X		
Stomach cancer	Male	Tobacco (cigarettes per capita)	X		
Stomach cancer	Female	Sanitation (proportion with access)		X	
Stomach cancer	Male	Improved water source (proportion with access)		X	
Stomach cancer	Female	Improved water source (proportion with access)		X	
Stomach cancer	Male	Tobacco (cigarettes per capita)	X		
Stomach cancer	Female	Tobacco (cigarettes per capita)	X		
Stomach cancer	Female	Tobacco (cigarettes per capita)	X		
Stomach cancer	Male	Cumulative cigarettes (15 years)	X		
Stomach cancer	Male	Log-transformed SEV scalar: Stomach C	X		
Stomach cancer	Female	Log-transformed SEV scalar: Stomach C	X		
Stomach cancer	Male	Diet high in sodium	X		
Stomach cancer	Female	Diet high in sodium	X		
Stomach cancer	Female	Fruits adjusted (g)		X	
Stomach cancer	Male	Sanitation (proportion with access)		X	
Stomach cancer	Male	Vegetables adjusted (g)		X	
Liver cancer	Male	Log-transformed SEV scalar: Liver C	X		
Liver cancer	Female	Log-transformed SEV scalar: Liver C	X		
Liver cancer	Female	Tobacco (cigarettes per capita)	X		
Liver cancer	Male	Cumulative cigarettes (20 years)	X		

Liver cancer	Male	Alcohol (litres per capita)	X		
Liver cancer	Female	Alcohol (litres per capita)	X		
Liver cancer	Male	Education (years per capita)			X
Liver cancer	Female	Education (years per capita)			X
Liver cancer	Male	LDI (I$ per capita)			X
Liver cancer	Female	LDI (I$ per capita)			X
Liver cancer	Male	Healthcare Access and Quality Index		X	
Liver cancer	Female	Healthcare Access and Quality Index		X	
Liver cancer	Female	Diabetes age-standardised prevalence (proportion)		X	
Liver cancer	Male	Mean BMI		X	
Liver cancer	Female	Mean BMI		X	
Liver cancer	Male	Socio-demographic Index			X
Liver cancer	Female	Socio-demographic Index			X
Liver cancer	Male	Hepatitis B (HBsAg) seroprevalence	X		
Liver cancer	Female	Hepatitis B (HBsAg) seroprevalence	X		
Liver cancer	Male	Hepatitis C (IgG) seroprevalence	X		
Liver cancer	Female	Hepatitis C (IgG) seroprevalence	X		
Liver cancer	Male	Red meats adjusted (g)		X	
Liver cancer	Female	Red meats adjusted (g)		X	
Liver cancer	Male	Tobacco (cigarettes per capita)	X		
Liver cancer	Male	Tobacco (cigarettes per capita)	X		
Liver cancer	Male	Cumulative cigarettes (15 years)	X		
Liver cancer	Female	Cumulative cigarettes (15 years)	X		
Liver cancer	Female	Cumulative cigarettes (20 years)	X		
Liver cancer	Male	Diabetes age-standardised prevalence (proportion)		X	
Liver cancer	Female	Tobacco (cigarettes per capita)	X		
Larynx cancer	Female	Socio-demographic Index			X
Larynx cancer	Female	Healthcare Access and Quality index		X	
Larynx cancer	Male	Healthcare Access and Quality Index		X	
Larynx cancer	Female	Population density (under 150 ppl/sqkm, proportion)		X	
Larynx cancer	Male	Population density (under 150 ppl/sqkm, proportion)		X	
Larynx cancer	Female	Population density (over 1000 ppl/sqkm, proportion)		X	
Larynx cancer	Male	Population density (over 1000 ppl/sqkm, proportion)		X	
Larynx cancer	Female	LDI (I$ per capita)			X
Larynx cancer	Male	LDI (I$ per capita)			X
Larynx cancer	Female	Education (years per capita)			X
Larynx cancer	Male	Education (years per capita)			X
Larynx cancer	Female	Alcohol (litres per capita)	X		
Larynx cancer	Male	Alcohol (litres per capita)	X		
Larynx cancer	Male	Socio-demographic Index			X
Larynx cancer	Male	Smoking prevalence		X	
Larynx cancer	Female	Smoking prevalence		X	
Larynx cancer	Male	Fruits adjusted (g)		X	

Larynx cancer	Male	Tobacco (cigarettes per capita)		X	
Larynx cancer	Male	Log-transformed SEV scalar: Larynx C	X		
Larynx cancer	Female	Cumulative cigarettes (20 years)		X	
Larynx cancer	Male	Cumulative cigarettes (20 years)		X	
Larynx cancer	Female	Cumulative cigarettes (15 years)		X	
Larynx cancer	Male	Cumulative cigarettes (15 years)		X	
Larynx cancer	Female	Tobacco (cigarettes per capita)		X	
Larynx cancer	Female	Tobacco (cigarettes per capita)		X	
Larynx cancer	Female	Fruits adjusted (g)		X	
Larynx cancer	Female	Log-transformed SEV scalar: Larynx C	X		
Larynx cancer	Female	Cumulative cigarettes (5 years)		X	
Larynx cancer	Male	Cumulative cigarettes (5 years)		X	
Larynx cancer	Female	Cumulative cigarettes (10 years)		X	
Larynx cancer	Male	Cumulative cigarettes (10 years)		X	
Larynx cancer	Female	Vegetables adjusted (g)		X	
Larynx cancer	Male	Vegetables adjusted (g)		X	
Larynx cancer	Male	Tobacco (cigarettes per capita)		X	
Tracheal, bronchus, and lung cancer	Female	Healthcare Access and Quality Index		X	
Tracheal, bronchus, and lung cancer	Male	LDI (I$ per capita)			X
Tracheal, bronchus, and lung cancer	Female	Smoking prevalence	X		
Tracheal, bronchus, and lung cancer	Male	Education (years per capita)			X
Tracheal, bronchus, and lung cancer	Female	Education (years per capita)			X
Tracheal, bronchus, and lung cancer	Male	Healthcare Access and Quality Index		X	
Tracheal, bronchus, and lung cancer	Female	LDI (I$ per capita)			X
Tracheal, bronchus, and lung cancer	Female	Socio-demographic Index			X
Tracheal, bronchus, and lung cancer	Female	Cumulative cigarettes (5 years)	X		
Tracheal, bronchus, and lung cancer	Female	Indoor air pollution (all cooking fuels)		X	
Tracheal, bronchus, and lung cancer	Male	Log-transformed age-standardised SEV scalar: Lung C	X		
Tracheal, bronchus, and lung cancer	Female	Log-transformed age-standardised SEV scalar: Lung C	X		
Tracheal, bronchus, and lung cancer	Male	Log-transformed SEV scalar: Lung C	X		
Tracheal, bronchus, and lung cancer	Female	Log-transformed SEV scalar: Lung C	X		
Tracheal, bronchus, and lung cancer	Male	Cumulative cigarettes (20 years)	X		
Tracheal, bronchus, and lung cancer	Female	Cumulative cigarettes (20 years)	X		
Tracheal, bronchus, and lung cancer	Male	Cumulative cigarettes (15 years)	X		
Tracheal, bronchus, and lung cancer	Male	Socio-demographic Index			X
Tracheal, bronchus, and lung cancer	Male	Tobacco (cigarettes per capita)	X		
Tracheal, bronchus, and lung cancer	Female	Cumulative cigarettes (15 years)	X		
Tracheal, bronchus, and lung cancer	Male	Cumulative cigarettes (5 years)	X		

Tracheal, bronchus, and lung cancer	Male	Cumulative cigarettes (10 years)	X		
Tracheal, bronchus, and lung cancer	Female	Cumulative cigarettes (10 years)	X		
Tracheal, bronchus, and lung cancer	Male	Smoking prevalence	X		
Tracheal, bronchus, and lung cancer	Male	Outdoor air pollution ($PM_{2.5}$)		X	
Tracheal, bronchus, and lung cancer	Female	Outdoor air pollution ($PM_{2.5}$)		X	
Tracheal, bronchus, and lung cancer	Male	Indoor air pollution (all cooking fuels)		X	
Tracheal, bronchus, and lung cancer	Female	Tobacco (cigarettes per capita)	X		
Breast cancer	Male	Cumulative cigarettes (10 years)		X	
Breast cancer	Female	Fruits adjusted (g)		X	
Breast cancer	Male	Vegetables adjusted (g)		X	
Breast cancer	Female	Vegetables adjusted (g)		X	
Breast cancer	Female	Cumulative cigarettes (10 years)		X	
Breast cancer	Male	Alcohol (litres per capita)	X		
Breast cancer	Female	Total fertility rate		X	
Breast cancer	Male	Log-transformed SEV scalar: Breast C	X		
Breast cancer	Female	Log-transformed SEV scalar: Breast C	X		
Breast cancer	Female	Age-specific fertility rate		X	
Breast cancer	Female	Age-specific fertility rate		X	
Breast cancer	Female	Total fertility rate		X	
Breast cancer	Female	Socio-demographic Index			X
Breast cancer	Male	Fruits adjusted (g)		X	
Breast cancer	Female	Mean BMI	X		
Breast cancer	Male	Socio-demographic Index			X
Breast cancer	Male	Education (years per capita)			X
Breast cancer	Female	Education (years per capita)			X
Breast cancer	Male	LDI (I$ per capita)			X
Breast cancer	Female	Alcohol (litres per capita)	X		
Breast cancer	Male	Healthcare Access and Quality Index		X	
Breast cancer	Female	Healthcare Access and Quality Index		X	
Breast cancer	Female	LDI (I$ per capita)			X
Breast cancer	Male	Mean BMI	X		
Cervical cancer	Female	Fruits adjusted (g)		X	
Cervical cancer	Female	Smoking prevalence		X	
Cervical cancer	Female	Cumulative cigarettes (5 years)	X		
Cervical cancer	Female	Total fertility rate		X	
Cervical cancer	Female	Total fertility rate		X	
Cervical cancer	Female	Cumulative cigarettes (15 years)	X		
Cervical cancer	Female	Age-specific fertility rate		X	
Cervical cancer	Female	Age-specific fertility rate		X	
Cervical cancer	Female	HIV age-standardised prevalence	X		
Cervical cancer	Female	HIV age-standardised prevalence	X		

Cervical cancer	Female	Vegetables adjusted (g)		X	
Cervical cancer	Female	Cumulative cigarettes (10 years)	X		
Cervical cancer	Female	Education (years per capita)			X
Cervical cancer	Female	LDI (I$ per capita)			X
Cervical cancer	Female	Healthcare Access and Quality Index		X	
Cervical cancer	Female	Socio-demographic Index			X
Uterine cancer	Female	Socio-demographic Index			X
Uterine cancer	Female	Log-transformed SEV scalar: Uterus C	X		
Uterine cancer	Female	Tobacco (cigarettes per capita)		X	
Uterine cancer	Female	Tobacco (cigarettes per capita)		X	
Uterine cancer	Female	Total fertility rate		X	
Uterine cancer	Female	Total fertility rate		X	
Uterine cancer	Female	Cumulative cigarettes (5 years)		X	
Uterine cancer	Female	Smoking prevalence		X	
Uterine cancer	Female	Cumulative cigarettes (10 years)		X	
Uterine cancer	Female	Education (years per capita)			X
Uterine cancer	Female	Fruits adjusted (g)		X	
Uterine cancer	Female	Healthcare Access and Quality Index		X	
Uterine cancer	Female	Diabetes age-standardised prevalence (proportion)		X	
Uterine cancer	Female	Mean BMI	X		
Uterine cancer	Female	Vegetables adjusted (g)		X	
Uterine cancer	Female	LDI (I$ per capita)			X
Prostate cancer	Male	Education (years per capita)			X
Prostate cancer	Male	LDI (I$ per capita)			X
Prostate cancer	Male	Healthcare Access and Quality Index		X	
Prostate cancer	Male	Socio-demographic Index			X
Prostate cancer	Male	Log-transformed SEV scalar: Prostate C	X		
Colon and rectum cancer	Male	Milk adjusted (g)		X	
Colon and rectum cancer	Female	Log-transformed SEV scalar: Colorect C	X		
Colon and rectum cancer	Male	Alcohol (litres per capita)	X		
Colon and rectum cancer	Female	Alcohol (litres per capita)	X		
Colon and rectum cancer	Male	Education (years per capita)			X
Colon and rectum cancer	Male	LDI (I$ per capita)			X
Colon and rectum cancer	Female	LDI (I$ per capita)			X
Colon and rectum cancer	Male	Healthcare Access and Quality Index		X	
Colon and rectum cancer	Female	Healthcare Access and Quality Index		X	
Colon and rectum cancer	Male	Mean BMI	X		
Colon and rectum cancer	Female	Mean BMI	X		
Colon and rectum cancer	Male	Socio-demographic Index			X
Colon and rectum cancer	Female	Socio-demographic Index			X
Colon and rectum cancer	Male	Red meats adjusted (g)	X		
Colon and rectum cancer	Female	Red meats adjusted (g)	X		
Colon and rectum cancer	Male	Smoking prevalence	X		

Colon and rectum cancer	Female	Smoking prevalence		X	
Colon and rectum cancer	Male	Fruits adjusted (g)		X	
Colon and rectum cancer	Female	Fruits adjusted (g)	X		
Colon and rectum cancer	Female	Fruits adjusted (g)		X	
Colon and rectum cancer	Male	Nuts and seeds adjusted (g)		X	
Colon and rectum cancer	Female	Nuts and seeds adjusted (g)		X	
Colon and rectum cancer	Male	PUFA adjusted (percent)		X	
Colon and rectum cancer	Female	PUFA adjusted (percent)		X	
Colon and rectum cancer	Male	Vegetables adjusted (g)		X	
Colon and rectum cancer	Female	Vegetables adjusted (g)	X		
Colon and rectum cancer	Female	Vegetables adjusted (g)		X	
Colon and rectum cancer	Male	Tobacco (cigarettes per capita)	X		
Colon and rectum cancer	Male	Log-transformed SEV scalar: Colorect C	X		
Colon and rectum cancer	Female	Milk adjusted (g)		X	
Colon and rectum cancer	Female	Tobacco (cigarettes per capita)		X	
Colon and rectum cancer	Female	Education (years per capita)			X
Lip and oral cavity cancer	Male	Cumulative cigarettes (20 years)	X		
Lip and oral cavity cancer	Female	Cumulative cigarettes (20 years)	X		
Lip and oral cavity cancer	Male	Alcohol (litres per capita)	X		
Lip and oral cavity cancer	Female	Alcohol (litres per capita)	X		
Lip and oral cavity cancer	Male	Education (years per capita)			X
Lip and oral cavity cancer	Female	Education (years per capita)			X
Lip and oral cavity cancer	Male	LDI (I$ per capita)			X
Lip and oral cavity cancer	Female	LDI (I$ per capita)			X
Lip and oral cavity cancer	Male	Healthcare Access and Quality Index		X	
Lip and oral cavity cancer	Female	Healthcare Access and Quality Index		X	
Lip and oral cavity cancer	Female	Health system access 2 (unitless)		X	
Lip and oral cavity cancer	Male	Socio-demographic Index			X
Lip and oral cavity cancer	Male	Tobacco (cigarettes per capita)	X		
Lip and oral cavity cancer	Male	Red meats adjusted (g)		X	
Lip and oral cavity cancer	Female	Socio-demographic Index			X
Lip and oral cavity cancer	Male	Log-transformed SEV scalar: Mouth C	X		
Lip and oral cavity cancer	Male	Cumulative cigarettes (5 years)	X		
Lip and oral cavity cancer	Female	Cumulative cigarettes (10 years)	X		
Lip and oral cavity cancer	Male	Cumulative cigarettes (15 years)	X		
Lip and oral cavity cancer	Male	Vegetables adjusted (g)		X	
Lip and oral cavity cancer	Male	Fruits adjusted (g)		X	
Lip and oral cavity cancer	Female	Smoking prevalence	X		
Lip and oral cavity cancer	Male	Smoking prevalence	X		
Lip and oral cavity cancer	Male	Cumulative cigarettes (10 years)	X		
Nasopharynx cancer	Male	Vegetables adjusted (g)		X	
Nasopharynx cancer	Female	Population density (under 150 ppl/sqkm, proportion)		X	
Nasopharynx cancer	Male	Population density (under 150 ppl/sqkm, proportion)		X	

Nasopharynx cancer	Female	Socio-demographic Index			X
Nasopharynx cancer	Male	Socio-demographic Index			X
Nasopharynx cancer	Female	Smoking prevalence	X		
Nasopharynx cancer	Male	Smoking prevalence	X		
Nasopharynx cancer	Female	Fruits adjusted (g)		X	
Nasopharynx cancer	Male	Fruits adjusted (g)		X	
Nasopharynx cancer	Female	Vegetables adjusted (g)		X	
Nasopharynx cancer	Female	Cumulative cigarettes (10 years)	X		
Nasopharynx cancer	Male	Tobacco (cigarettes per capita)	X		
Nasopharynx cancer	Female	Cumulative cigarettes (5 years)	X		
Nasopharynx cancer	Male	Cumulative cigarettes (5 years)	X		
Nasopharynx cancer	Female	Tobacco (cigarettes per capita)	X		
Nasopharynx cancer	Female	Population density (over 1000 ppl/sqkm, proportion)		X	
Nasopharynx cancer	Female	Cumulative cigarettes (15 years)	X		
Nasopharynx cancer	Male	Cumulative cigarettes (15 years)	X		
Nasopharynx cancer	Female	Cumulative cigarettes (20 years)	X		
Nasopharynx cancer	Male	Cumulative cigarettes (20 years)	X		
Nasopharynx cancer	Female	Log-transformed SEV scalar: Nasoph C	X		
Nasopharynx cancer	Male	Log-transformed SEV scalar: Nasoph C	X		
Nasopharynx cancer	Male	Cumulative cigarettes (10 years)	X		
Nasopharynx cancer	Male	LDI (I$ per capita)			X
Nasopharynx cancer	Male	Population density (over 1000 ppl/sqkm, proportion)		X	
Nasopharynx cancer	Male	Education (years per capita)			X
Nasopharynx cancer	Female	Education (years per capita)			X
Nasopharynx cancer	Female	LDI (I$ per capita)			X
Nasopharynx cancer	Male	Alcohol (litres per capita)	X		
Nasopharynx cancer	Female	Alcohol (litres per capita)	X		
Other pharynx cancer	Male	Vegetables adjusted (g)		X	
Other pharynx cancer	Female	Vegetables adjusted (g)		X	
Other pharynx cancer	Male	Cumulative cigarettes (5 years)		X	
Other pharynx cancer	Female	Cumulative cigarettes (5 years)		X	
Other pharynx cancer	Female	Alcohol (litres per capita)	X		
Other pharynx cancer	Male	Fruits adjusted (g)		X	
Other pharynx cancer	Male	Education (years per capita)			X
Other pharynx cancer	Female	Education (years per capita)			X
Other pharynx cancer	Male	Log-transformed SEV scalar: Oth Phar C	X		
Other pharynx cancer	Female	Log-transformed SEV scalar: Oth Phar C	X		
Other pharynx cancer	Male	Alcohol (litres per capita)	X		
Other pharynx cancer	Female	Smoking prevalence	X		
Other pharynx cancer	Female	Fruits adjusted (g)		X	
Other pharynx cancer	Female	Socio-demographic Index			X
Other pharynx cancer	Male	Socio-demographic Index			X
Other pharynx cancer	Female	Population density (under 150 ppl/sqkm, proportion)		X	

Other pharynx cancer	Male	Population density (under 150 ppl/sqkm, proportion)		X	
Other pharynx cancer	Female	Population density (over 1000 ppl/sqkm, proportion)		X	
Other pharynx cancer	Male	Population density (over 1000 ppl/sqkm, proportion)		X	
Other pharynx cancer	Female	LDI (I$ per capita)			X
Other pharynx cancer	Male	LDI (I$ per capita)			X
Other pharynx cancer	Male	Smoking prevalence	X		
Gallbladder and biliary tract cancer	Female	Socio-demographic Index			X
Gallbladder and biliary tract cancer	Male	Mean BMI	X		
Gallbladder and biliary tract cancer	Male	Socio-demographic Index			X
Gallbladder and biliary tract cancer	Female	Mean BMI	X		
Gallbladder and biliary tract cancer	Male	Diabetes age-standardised prevalence (proportion)		X	
Gallbladder and biliary tract cancer	Female	Diabetes age-standardised prevalence (proportion)		X	
Gallbladder and biliary tract cancer	Female	LDI (I$ per capita)			X
Gallbladder and biliary tract cancer	Female	Healthcare Access and Quality Index		X	
Gallbladder and biliary tract cancer	Male	Education (years per capita)			X
Gallbladder and biliary tract cancer	Male	LDI (I$ per capita)			X
Gallbladder and biliary tract cancer	Female	Education (years per capita)			X
Gallbladder and biliary tract cancer	Male	Alcohol (litres per capita)		X	
Gallbladder and biliary tract cancer	Female	Alcohol (litres per capita)		X	
Gallbladder and biliary tract cancer	Male	Healthcare Access and Quality Index		X	
Gallbladder and biliary tract cancer	Female	Smoking prevalence		X	
Gallbladder and biliary tract cancer	Male	Vegetables adjusted (g)		X	
Gallbladder and biliary tract cancer	Female	Fruits adjusted (g)		X	
Gallbladder and biliary tract cancer	Male	Smoking prevalence		X	
Gallbladder and biliary tract cancer	Male	Log-transformed SEV scalar: Gallblad C	X		
Gallbladder and biliary tract cancer	Female	Log-transformed SEV scalar: Gallblad C	X		
Gallbladder and biliary tract cancer	Female	Tobacco (cigarettes per capita)		X	
Gallbladder and biliary tract cancer	Male	Cumulative cigarettes (5 years)		X	
Gallbladder and biliary tract cancer	Male	Tobacco (cigarettes per capita)		X	
Gallbladder and biliary tract cancer	Male	Cumulative cigarettes (10 years)		X	
Gallbladder and biliary tract cancer	Female	Cumulative cigarettes (10 years)		X	
Gallbladder and biliary tract cancer	Female	Vegetables adjusted (g)		X	
Gallbladder and biliary tract cancer	Male	Fruits adjusted (g)		X	
Gallbladder and biliary tract cancer	Female	Cumulative cigarettes (5 years)		X	
Pancreatic cancer	Female	Smoking prevalence	X		
Pancreatic cancer	Male	Smoking prevalence	X		
Pancreatic cancer	Male	Energy unadjusted (kcal)		X	
Pancreatic cancer	Female	Energy unadjusted (kcal)		X	
Pancreatic cancer	Female	Red meats adjusted (g)		X	
Pancreatic cancer	Female	Socio-demographic Index			X
Pancreatic cancer	Male	Socio-demographic Index			X
Pancreatic cancer	Female	Mean BMI	X		
Pancreatic cancer	Male	Mean BMI	X		

Pancreatic cancer	Female	Diabetes age-standardised prevalence (proportion)		X	
Pancreatic cancer	Male	Red meats adjusted (g)		X	
Pancreatic cancer	Male	Fruits adjusted (g)		X	
Pancreatic cancer	Male	Vegetables adjusted (g)		X	
Pancreatic cancer	Female	Vegetables adjusted (g)		X	
Pancreatic cancer	Female	Vegetables adjusted (g)	X		
Pancreatic cancer	Male	Cumulative cigarettes (10 years)	X		
Pancreatic cancer	Female	Cumulative cigarettes (10 years)	X		
Pancreatic cancer	Male	Cumulative cigarettes (5 years)		X	
Pancreatic cancer	Female	Cumulative cigarettes (5 years)	X		
Pancreatic cancer	Male	Tobacco (cigarettes per capita)	X		
Pancreatic cancer	Female	Tobacco (cigarettes per capita)	X		
Pancreatic cancer	Male	Cumulative cigarettes (20 years)	X		
Pancreatic cancer	Female	Cumulative cigarettes (20 years)	X		
Pancreatic cancer	Male	Log-transformed SEV scalar: Pancreas C	X		
Pancreatic cancer	Female	Log-transformed SEV scalar: Pancreas C	X		
Pancreatic cancer	Female	Fruits adjusted (g)		X	
Pancreatic cancer	Male	Diabetes age-standardised prevalence (proportion)		X	
Pancreatic cancer	Female	LDI (I$ per capita)			X
Pancreatic cancer	Male	Healthcare Access and Quality Index		X	
Pancreatic cancer	Female	Healthcare Access and Quality Index		X	
Pancreatic cancer	Male	Alcohol (litres per capita)	X		
Pancreatic cancer	Male	Education (years per capita)			X
Pancreatic cancer	Female	Education (years per capita)			X
Pancreatic cancer	Male	LDI (I$ per capita)			X
Pancreatic cancer	Female	Alcohol (litres per capita)		X	
Malignant skin melanoma	Male	Alcohol (litres per capita)		X	
Malignant skin melanoma	Male	Alcohol (litres per capita)	X		
Malignant skin melanoma	Female	Alcohol (litres per capita)	X		
Malignant skin melanoma	Male	Education (years per capita)			X
Malignant skin melanoma	Female	Education (years per capita)			X
Malignant skin melanoma	Male	LDI (I$ per capita)			X
Malignant skin melanoma	Female	LDI (I$ per capita)			X
Malignant skin melanoma	Male	Healthcare Access and Quality Index		X	
Malignant skin melanoma	Female	Healthcare Access and Quality Index		X	
Malignant skin melanoma	Male	Socio-demographic Index			X
Malignant skin melanoma	Female	Socio-demographic Index			X
Malignant skin melanoma	Male	Vegetables adjusted (g)		X	
Malignant skin melanoma	Female	Vegetables adjusted (g)		X	
Malignant skin melanoma	Male	Latitude 15 to 30 (proportion)		X	
Malignant skin melanoma	Female	Latitude 15 to 30 (proportion)		X	
Malignant skin melanoma	Male	Latitude 30 to 45 (proportion)		X	
Malignant skin melanoma	Male	Latitude over 45 (proportion)		X	

Malignant skin melanoma	Female	Latitude over 45 (proportion)		X	
Malignant skin melanoma	Male	Latitude under 15 (proportion)		X	
Malignant skin melanoma	Female	Latitude under 15 (proportion)		X	
Malignant skin melanoma	Male	Fruits adjusted (g)		X	
Malignant skin melanoma	Female	Fruits adjusted (g)		X	
Malignant skin melanoma	Female	Latitude 30 to 45 (proportion)		X	
Ovarian cancer	Female	Smoking prevalence		X	
Ovarian cancer	Female	Mean BMI		X	
Ovarian cancer	Female	Diabetes age-standardised prevalence (proportion)		X	
Ovarian cancer	Female	Healthcare Access and Quality Index		X	
Ovarian cancer	Female	Log-transformed SEV scalar: Ovary C	X		
Ovarian cancer	Female	LDI (I$ per capita)			X
Ovarian cancer	Female	Education (years per capita)			X
Ovarian cancer	Female	Alcohol (litres per capita)	X		
Ovarian cancer	Female	Socio-demographic Index			X
Ovarian cancer	Female	Contraception (modern) prevalence (proportion)	X		
Ovarian cancer	Female	Cumulative cigarettes (20 years)	X		
Ovarian cancer	Female	Energy unadjusted (kcal)		X	
Ovarian cancer	Female	Total fertility rate		X	
Ovarian cancer	Female	Vegetables adjusted (g)		X	
Ovarian cancer	Female	Fruits adjusted (g)		X	
Ovarian cancer	Female	Tobacco (cigarettes per capita)	X		
Testicular cancer	Male	Education (years per capita)			X
Testicular cancer	Male	LDI (I$ per capita)			X
Testicular cancer	Male	Healthcare Access and Quality Index		X	
Testicular cancer	Male	Socio-demographic Index			X
Testicular cancer	Male	Cumulative cigarettes (10 years)		X	
Testicular cancer	Male	Cumulative cigarettes (5 years)		X	
Testicular cancer	Male	Cumulative cigarettes (15 years)		X	
Kidney cancer	Male	Log-transformed SEV scalar: Kidney C	X		
Kidney cancer	Female	Log-transformed SEV scalar: Kidney C	X		
Kidney cancer	Female	Cumulative cigarettes (15 years)	X		
Kidney cancer	Male	Cumulative cigarettes (10 years)	X		
Kidney cancer	Female	Cumulative cigarettes (5 years)	X		
Kidney cancer	Female	Education (years per capita)			X
Kidney cancer	Male	LDI (I$ per capita)			X
Kidney cancer	Female	LDI (I$ per capita)			X
Kidney cancer	Male	Diabetes age-standardised prevalence (proportion)		X	
Kidney cancer	Female	Diabetes age-standardised prevalence (proportion)		X	
Kidney cancer	Female	Mean BMI	X		
Kidney cancer	Male	Socio-demographic Index			X
Kidney cancer	Female	Socio-demographic Index			X
Kidney cancer	Male	Systolic blood pressure (mmHg)		X	

Kidney cancer	Female	Systolic blood pressure (mmHg)		X	
Kidney cancer	Male	Smoking prevalence		X	
Kidney cancer	Female	Smoking prevalence		X	
Kidney cancer	Male	Alcohol (litres per capita)		X	
Kidney cancer	Female	Cumulative cigarettes (10 years)	X		
Kidney cancer	Male	Cumulative cigarettes (5 years)	X		
Kidney cancer	Male	Cumulative cigarettes (15 years)	X		
Kidney cancer	Male	Mean BMI	X		
Kidney cancer	Female	Alcohol (litres per capita)		X	
Kidney cancer	Male	Education (years per capita)			X
Bladder cancer	Female	Alcohol (litres per capita)		X	
Bladder cancer	Female	Cumulative cigarettes (10 years)	X		
Bladder cancer	Male	Cumulative cigarettes (5 years)	X		
Bladder cancer	Female	Cumulative cigarettes (5 years)	X		
Bladder cancer	Male	Cumulative cigarettes (15 years)	X		
Bladder cancer	Female	Cumulative cigarettes (15 years)	X		
Bladder cancer	Male	Log-transformed SEV scalar: Bladder C	X		
Bladder cancer	Female	Log-transformed SEV scalar: Bladder C	X		
Bladder cancer	Male	Vegetables adjusted (g)		X	
Bladder cancer	Female	Fruits adjusted (g)		X	
Bladder cancer	Male	Fruits adjusted (g)		X	
Bladder cancer	Female	Smoking prevalence	X		
Bladder cancer	Male	Smoking prevalence	X		
Bladder cancer	Female	Socio-demographic Index			X
Bladder cancer	Male	Socio-demographic Index			X
Bladder cancer	Female	Healthcare Access and Quality Index		X	
Bladder cancer	Male	Healthcare Access and Quality Index		X	
Bladder cancer	Female	LDI (I$ per capita)			X
Bladder cancer	Female	Education (years per capita)			X
Bladder cancer	Male	Education (years per capita)			X
Bladder cancer	Male	Alcohol (litres per capita)		X	
Bladder cancer	Male	LDI (I$ per capita)			X
Bladder cancer	Female	Vegetables adjusted (g)		X	
Bladder cancer	Male	Cumulative cigarettes (10 years)	X		
Brain and nervous system cancer	Female	Fruits adjusted (g)		X	
Brain and nervous system cancer	Female	Vegetables adjusted (g)		X	
Brain and nervous system cancer	Male	Fruits adjusted (g)		X	
Brain and nervous system cancer	Male	Smoking prevalence	X		
Brain and nervous system cancer	Female	Smoking prevalence	X		
Brain and nervous system cancer	Male	Red meats adjusted (g)		X	
Brain and nervous system cancer	Female	Red meats adjusted (g)		X	
Brain and nervous system cancer	Male	Systolic blood pressure (mmHg)		X	
Brain and nervous system cancer	Male	Cholesterol (total, mean per capita)		X	

Brain and nervous system cancer	Male	Vegetables adjusted (g)		X	
Brain and nervous system cancer	Female	Cholesterol (total, mean per capita)		X	
Brain and nervous system cancer	Female	Socio-demographic Index			X
Brain and nervous system cancer	Male	Healthcare Access and Quality Index		X	
Brain and nervous system cancer	Female	Healthcare Access and Quality Index		X	
Brain and nervous system cancer	Male	LDI (I$ per capita)			X
Brain and nervous system cancer	Female	LDI (I$ per capita)			X
Brain and nervous system cancer	Male	Education (years per capita)			X
Brain and nervous system cancer	Female	Education (years per capita)			X
Brain and nervous system cancer	Male	Alcohol (litres per capita)	X		
Brain and nervous system cancer	Female	Alcohol (litres per capita)	X		
Brain and nervous system cancer	Male	Socio-demographic Index			X
Brain and nervous system cancer	Female	Cumulative cigarettes (10 years)	X		
Brain and nervous system cancer	Female	Systolic blood pressure (mmHg)		X	
Brain and nervous system cancer	Female	Cumulative cigarettes (15 years)	X		
Brain and nervous system cancer	Male	Cumulative cigarettes (15 years)	X		
Brain and nervous system cancer	Male	Cumulative cigarettes (10 years)	X		
Thyroid cancer	Male	Tobacco (cigarettes per capita)		X	
Thyroid cancer	Female	Tobacco (cigarettes per capita)		X	
Thyroid cancer	Male	Improved water source (proportion with access)		X	
Thyroid cancer	Female	Improved water source (proportion with access)		X	
Thyroid cancer	Male	Sanitation (proportion with access)		X	
Thyroid cancer	Female	Sanitation (proportion with access)		X	
Thyroid cancer	Male	Vegetables adjusted (g)		X	
Thyroid cancer	Female	Education (years per capita)			X
Thyroid cancer	Male	Education (years per capita)			X
Thyroid cancer	Female	LDI (I$ per capita)			X
Thyroid cancer	Male	LDI (I$ per capita)			X
Thyroid cancer	Female	Healthcare Access and Quality Index		X	
Thyroid cancer	Male	Healthcare Access and Quality Index		X	
Thyroid cancer	Female	Mean BMI		X	
Thyroid cancer	Female	Socio-demographic Index			X
Thyroid cancer	Male	Socio-demographic Index			X
Thyroid cancer	Female	Red meats adjusted (g)		X	
Thyroid cancer	Male	Red meats adjusted (g)		X	
Thyroid cancer	Female	Vegetables adjusted (g)		X	
Thyroid cancer	Female	Smoking prevalence		X	
Thyroid cancer	Male	Smoking prevalence		X	
Thyroid cancer	Male	Smoking prevalence		X	
Thyroid cancer	Male	Smoking prevalence	X		
Thyroid cancer	Male	Smoking prevalence	X		
Thyroid cancer	Female	Fruits adjusted (g)		X	
Thyroid cancer	Male	Fruits adjusted (g)		X	

Thyroid cancer	Male	Mean BMI		X	
Thyroid cancer	Male	Alcohol (litres per capita)	X		
Thyroid cancer	Female	Alcohol (litres per capita)	X		
Thyroid cancer	Male	Log-transformed SEV scalar: Thyroid C	X		
Thyroid cancer	Female	Log-transformed SEV scalar: Thyroid C	X		
Mesothelioma	Female	Asbestos consumption (metric tons per year per capita)	X		
Mesothelioma	Female	Healthcare Access and Quality Index		X	
Mesothelioma	Male	Healthcare Access and Quality Index		X	
Mesothelioma	Female	Socio-demographic Index			X
Mesothelioma	Male	Socio-demographic Index			X
Mesothelioma	Female	Indoor air pollution (all cooking fuels)	X		
Mesothelioma	Male	Indoor air pollution (all cooking fuels)	X		
Mesothelioma	Male	LDI (I$ per capita)			X
Mesothelioma	Male	Asbestos consumption (metric tons per year per capita)	X		
Mesothelioma	Female	LDI (I$ per capita)			X
Mesothelioma	Male	Education (years per capita)			X
Mesothelioma	Female	Education (years per capita)			X
Mesothelioma	Male	Population density (over 1000 ppl/sqkm, proportion)		X	
Mesothelioma	Female	Population density (over 1000 ppl/sqkm, proportion)		X	
Mesothelioma	Male	Cumulative cigarettes (5 years)	X		
Mesothelioma	Female	Log-transformed SEV scalar: Mesothel	X		
Mesothelioma	Male	Gold production (kg) per capita		X	
Mesothelioma	Female	Gold production (kg) per capita		X	
Mesothelioma	Male	Gold production (binary)		X	
Mesothelioma	Female	Gold production (binary)		X	
Mesothelioma	Female	Smoking prevalence	X		
Mesothelioma	Male	Smoking prevalence	X		
Mesothelioma	Female	Cumulative cigarettes (5 years)	X		
Mesothelioma	Female	Asbestos production (kg) per capita		X	
Mesothelioma	Female	Asbestos production (binary)	X		
Hodgkin lymphoma	Male	LDI (I$ per capita)			X
Hodgkin lymphoma	Female	Socio-demographic Index			X
Hodgkin lymphoma	Male	Socio-demographic Index			X
Hodgkin lymphoma	Female	Healthcare Access and Quality Index		X	
Hodgkin lymphoma	Male	Education (years per capita)			X
Hodgkin lymphoma	Female	LDI (I$ per capita)			X
Hodgkin lymphoma	Female	Education (years per capita)			X
Hodgkin lymphoma	Male	Healthcare Access and Quality Index		X	
Non-Hodgkin lymphoma	Female	Cumulative cigarettes (10 years)		X	
Non-Hodgkin lymphoma	Female	Smoking prevalence		X	
Non-Hodgkin lymphoma	Male	Smoking prevalence		X	
Non-Hodgkin lymphoma	Female	Socio-demographic Index			X
Non-Hodgkin lymphoma	Male	Socio-demographic Index			X

Non-Hodgkin lymphoma	Female	Healthcare Access and Quality Index		X	
Non-Hodgkin lymphoma	Male	Healthcare Access and Quality Index		X	
Non-Hodgkin lymphoma	Female	LDI (I$ per capita)			X
Non-Hodgkin lymphoma	Male	LDI (I$ per capita)			X
Non-Hodgkin lymphoma	Female	Alcohol (litres per capita)		X	
Non-Hodgkin lymphoma	Male	Alcohol (litres per capita)		X	
Non-Hodgkin lymphoma	Female	Total fertility rate			X
Non-Hodgkin lymphoma	Male	Cumulative cigarettes (10 years)		X	
Multiple myeloma	Male	Tobacco (cigarettes per capita)	X		
Multiple myeloma	Female	Tobacco (cigarettes per capita)	X		
Multiple myeloma	Male	Improved water source (proportion with access)		X	
Multiple myeloma	Female	Sanitation (proportion with access)		X	
Multiple myeloma	Female	Red meats adjusted (g)		X	
Multiple myeloma	Male	Red meats adjusted (g)		X	
Multiple myeloma	Female	Socio-demographic Index			X
Multiple myeloma	Male	Socio-demographic Index			X
Multiple myeloma	Female	Improved water source (proportion with access)		X	
Multiple myeloma	Male	Mean BMI		X	
Multiple myeloma	Male	Smoking prevalence	X		
Multiple myeloma	Female	Healthcare Access and Quality Index		X	
Multiple myeloma	Female	LDI (I$ per capita)			X
Multiple myeloma	Male	LDI (I$ per capita)			X
Multiple myeloma	Female	Education (years per capita)			X
Multiple myeloma	Male	Education (years per capita)			X
Multiple myeloma	Female	Alcohol (litres per capita)	X		
Multiple myeloma	Male	Alcohol (litres per capita)	X		
Multiple myeloma	Male	Healthcare Access and Quality Index		X	
Multiple myeloma	Female	Smoking prevalence	X		
Multiple myeloma	Female	Mean BMI		X	
Multiple myeloma	Male	Sanitation (proportion with access)		X	
Multiple myeloma	Female	Fruits adjusted (g)		X	
Multiple myeloma	Male	Vegetables adjusted (g)		X	
Multiple myeloma	Female	Vegetables adjusted (g)		X	
Multiple myeloma	Male	Fruits adjusted (g)		X	
Leukaemia	Female	Tobacco (cigarettes per capita)		X	
Leukaemia	Female	Tobacco (cigarettes per capita)		X	
Leukaemia	Male	Tobacco (cigarettes per capita)		X	
Leukaemia	Male	Tobacco (cigarettes per capita)		X	
Leukaemia	Female	Cumulative cigarettes (15 years)		X	
Leukaemia	Female	Cumulative cigarettes (20 years)		X	
Leukaemia	Male	Cumulative cigarettes (20 years)		X	
Leukaemia	Female	Log-transformed SEV scalar: Leukaemia	X		
Leukaemia	Male	Log-transformed SEV scalar: Leukaemia	X		

Leukaemia	Female	Log-transformed age-standardised SEV scalar: Leukaemia	X		
Leukaemia	Male	Cumulative cigarettes (15 years)		X	
Leukaemia	Male	Cumulative cigarettes (5 years)		X	
Leukaemia	Male	Alcohol (litres per capita)		X	
Leukaemia	Male	Cumulative cigarettes (10 years)		X	
Leukaemia	Female	Cumulative cigarettes (10 years)		X	
Leukaemia	Male	Smoking prevalence		X	
Leukaemia	Female	Smoking prevalence		X	
Leukaemia	Male	Socio-demographic Index			X
Leukaemia	Female	Socio-demographic Index			X
Leukaemia	Female	Healthcare Access and Quality Index		X	
Leukaemia	Male	LDI (I$ per capita)			X
Leukaemia	Female	LDI (I$ per capita)			X
Leukaemia	Male	Education (years per capita)			X
Leukaemia	Female	Education (years per capita)			X
Leukaemia	Female	Cumulative cigarettes (5 years)		X	
Leukaemia	Male	Log-transformed age-standardised SEV scalar: Leukaemia	X		
Leukaemia	Female	Alcohol (litres per capita)		X	
Acute lymphoid leukaemia	Female	Socio-demographic Index			X
Acute lymphoid leukaemia	Male	Smoking prevalence		X	
Acute lymphoid leukaemia	Female	Smoking prevalence		X	
Acute lymphoid leukaemia	Male	Socio-demographic Index			X
Acute lymphoid leukaemia	Female	Cumulative cigarettes (5 years)		X	
Acute lymphoid leukaemia	Male	LDI (I$ per capita)			X
Acute lymphoid leukaemia	Female	LDI (I$ per capita)			X
Acute lymphoid leukaemia	Male	Education (years per capita)			X
Acute lymphoid leukaemia	Female	Education (years per capita)			X
Acute lymphoid leukaemia	Male	Alcohol (litres per capita)		X	
Acute lymphoid leukaemia	Female	Alcohol (litres per capita)		X	
Acute lymphoid leukaemia	Male	Cumulative cigarettes (5 years)		X	
Acute lymphoid leukaemia	Female	Tobacco (cigarettes per capita)		X	
Acute lymphoid leukaemia	Male	Tobacco (cigarettes per capita)		X	
Acute lymphoid leukaemia	Female	Cumulative cigarettes (15 years)		X	
Acute lymphoid leukaemia	Male	Cumulative cigarettes (15 years)		X	
Acute lymphoid leukaemia	Female	Cumulative cigarettes (20 years)		X	
Acute lymphoid leukaemia	Male	Cumulative cigarettes (20 years)		X	
Acute lymphoid leukaemia	Female	Log-transformed SEV scalar: Leukaemia	X		
Acute lymphoid leukaemia	Male	Log-transformed SEV scalar: Leukaemia	X		
Acute lymphoid leukaemia	Female	Log-transformed age-standardised SEV scalar: Leukaemia	X		
Acute lymphoid leukaemia	Male	Log-transformed age-standardised SEV scalar: Leukaemia	X		
Acute lymphoid leukaemia	Female	Cumulative cigarettes (10 years)		X	
Acute lymphoid leukaemia	Male	Cumulative cigarettes (10 years)		X	

Chronic lymphoid leukaemia	Male	Smoking prevalence		X	
Chronic lymphoid leukaemia	Female	Socio-demographic Index			X
Chronic lymphoid leukaemia	Male	Cumulative cigarettes (20 years)		X	
Chronic lymphoid leukaemia	Female	Smoking prevalence		X	
Chronic lymphoid leukaemia	Male	Cumulative cigarettes (10 years)		X	
Chronic lymphoid leukaemia	Female	Cumulative cigarettes (10 years)		X	
Chronic lymphoid leukaemia	Male	Cumulative cigarettes (5 years)		X	
Chronic lymphoid leukaemia	Female	Cumulative cigarettes (5 years)		X	
Chronic lymphoid leukaemia	Male	Tobacco (cigarettes per capita)		X	
Chronic lymphoid leukaemia	Female	Tobacco (cigarettes per capita)		X	
Chronic lymphoid leukaemia	Female	Cumulative cigarettes (15 years)		X	
Chronic lymphoid leukaemia	Female	Cumulative cigarettes (20 years)		X	
Chronic lymphoid leukaemia	Male	Log-transformed SEV scalar: Leukaemia	X		
Chronic lymphoid leukaemia	Female	Log-transformed SEV scalar: Leukaemia	X		
Chronic lymphoid leukaemia	Male	Log-transformed age-standardised SEV scalar: Leukaemia	X		
Chronic lymphoid leukaemia	Female	Log-transformed age-standardised SEV scalar: Leukaemia	X		
Chronic lymphoid leukaemia	Male	Socio-demographic Index			X
Chronic lymphoid leukaemia	Female	LDI (I$ per capita)			X
Chronic lymphoid leukaemia	Male	Cumulative cigarettes (15 years)		X	
Chronic lymphoid leukaemia	Female	Education (years per capita)			X
Chronic lymphoid leukaemia	Male	Education (years per capita)			X
Chronic lymphoid leukaemia	Female	Alcohol (litres per capita)		X	
Chronic lymphoid leukaemia	Male	Alcohol (litres per capita)		X	
Chronic lymphoid leukaemia	Male	LDI (I$ per capita)			X
Acute myeloid leukaemia	Female	Socio-demographic Index			X
Acute myeloid leukaemia	Male	Socio-demographic Index			X
Acute myeloid leukaemia	Female	Smoking prevalence		X	
Acute myeloid leukaemia	Male	Smoking prevalence		X	
Acute myeloid leukaemia	Female	Cumulative cigarettes (10 years)		X	
Acute myeloid leukaemia	Male	Cumulative cigarettes (10 years)		X	
Acute myeloid leukaemia	Female	Cumulative cigarettes (5 years)		X	
Acute myeloid leukaemia	Male	Cumulative cigarettes (5 years)		X	
Acute myeloid leukaemia	Female	Cumulative cigarettes (15 years)		X	
Acute myeloid leukaemia	Male	Healthcare Access and Quality Index		X	
Acute myeloid leukaemia	Male	Cumulative cigarettes (15 years)		X	
Acute myeloid leukaemia	Female	Cumulative cigarettes (20 years)		X	
Acute myeloid leukaemia	Male	Cumulative cigarettes (20 years)		X	
Acute myeloid leukaemia	Female	Log-transformed SEV scalar: Leukaemia	X		
Acute myeloid leukaemia	Male	Log-transformed SEV scalar: Leukaemia	X		
Acute myeloid leukaemia	Female	Log-transformed age-standardised SEV scalar: Leukaemia	X		
Acute myeloid leukaemia	Male	Log-transformed age-standardised SEV scalar: Leukaemia	X		
Acute myeloid leukaemia	Male	Tobacco (cigarettes per capita)		X	

Acute myeloid leukaemia	Male	LDI (I$ per capita)			X
Acute myeloid leukaemia	Female	Tobacco (cigarettes per capita)		X	
Acute myeloid leukaemia	Male	Education (years per capita)			X
Acute myeloid leukaemia	Female	Education (years per capita)			X
Acute myeloid leukaemia	Male	Alcohol (litres per capita)		X	
Acute myeloid leukaemia	Female	Alcohol (litres per capita)		X	
Acute myeloid leukaemia	Female	LDI (I$ per capita)			X
Chronic myeloid leukaemia	Female	Healthcare Access and Quality Index		X	
Chronic myeloid leukaemia	Male	Healthcare Access and Quality Index		X	
Chronic myeloid leukaemia	Female	Socio-demographic Index			X
Chronic myeloid leukaemia	Male	Socio-demographic Index			X
Chronic myeloid leukaemia	Female	Smoking prevalence		X	
Chronic myeloid leukaemia	Male	Smoking prevalence		X	
Chronic myeloid leukaemia	Female	Cumulative cigarettes (10 years)		X	
Chronic myeloid leukaemia	Male	Cumulative cigarettes (10 years)		X	
Chronic myeloid leukaemia	Female	Tobacco (cigarettes per capita)		X	
Chronic myeloid leukaemia	Male	Cumulative cigarettes (5 years)		X	
Chronic myeloid leukaemia	Male	Tobacco (cigarettes per capita)		X	
Chronic myeloid leukaemia	Female	Cumulative cigarettes (15 years)		X	
Chronic myeloid leukaemia	Male	Cumulative cigarettes (15 years)		X	
Chronic myeloid leukaemia	Female	Cumulative cigarettes (20 years)		X	
Chronic myeloid leukaemia	Male	Cumulative cigarettes (20 years)		X	
Chronic myeloid leukaemia	Female	Log-transformed age-standardised SEV scalar: Leukaemia	X		
Chronic myeloid leukaemia	Male	Log-transformed age-standardised SEV scalar: Leukaemia	X		
Chronic myeloid leukaemia	Female	Cumulative cigarettes (5 years)		X	
Chronic myeloid leukaemia	Male	LDI (I$ per capita)			X
Chronic myeloid leukaemia	Male	Education (years per capita)			X
Chronic myeloid leukaemia	Female	Education (years per capita)			X
Chronic myeloid leukaemia	Male	Alcohol (litres per capita)		X	
Chronic myeloid leukaemia	Female	Alcohol (litres per capita)		X	
Chronic myeloid leukaemia	Female	LDI (I$ per capita)			X
Other leukaemia	Male	Education (years per capita)			X
Other leukaemia	Male	Cumulative cigarettes (20 years)		X	
Other leukaemia	Female	Cumulative cigarettes (20 years)		X	
Other leukaemia	Female	Education (years per capita)			X
Other leukaemia	Female	Log-transformed SEV scalar: Leukaemia	X		
Other leukaemia	Male	Log-transformed SEV scalar: Leukaemia	X		
Other leukaemia	Male	Alcohol (litres per capita)		X	
Other leukaemia	Female	Alcohol (litres per capita)		X	
Other leukaemia	Male	Tobacco (cigarettes per capita)		X	
Other leukaemia	Female	Tobacco (cigarettes per capita)		X	
Other leukaemia	Male	Cumulative cigarettes (15 years)		X	

Other leukaemia	Male	Cumulative cigarettes (5 years)		X	
Other leukaemia	Male	Cumulative cigarettes (10 years)		X	
Other leukaemia	Female	Cumulative cigarettes (10 years)		X	
Other leukaemia	Male	Smoking prevalence		X	
Other leukaemia	Female	Smoking prevalence		X	
Other leukaemia	Male	Socio-demographic Index			X
Other leukaemia	Female	Socio-demographic Index			X
Other leukaemia	Male	LDI (I$ per capita)			X
Other leukaemia	Female	LDI (I$ per capita)			X
Other leukaemia	Female	Cumulative cigarettes (5 years)		X	
Other leukaemia	Female	Cumulative cigarettes (15 years)		X	

Level 1 covariates are weighted more heavily that Level 2 covariates and Level 2 covariates are weighted more heavily than Level 3 covariates.

CoDCorrect

CODEm models estimate the individual cause-level mortality without taking into account the all-cause mortality. To ensure that all single causes add up to the all-cause mortality and that all child-causes add up to the parent cause, an algorithm called "CoDCorrect" is used. Details regarding the algorithm can be found in Section 4.2 in Supplement 1 to "Global, regional, and national age-sex-specific mortality for 282 causes of death in 195 countries and territories, 1980–2017: a systematic analysis for the Global Burden of Disease Study 2017".[4]

Incidence estimation

GBD cancer incidence estimates were generated by dividing final mortality estimates (after CoDCorrect adjustment) by the MIR for the specific cancer (step 1 in "Flowchart of GBD 2017 cancer incidence, prevalence and YLD estimation"). To propagate uncertainty from the MIRs and the mortality estimates to incidence this process was done at the 1000 draw level. It was assumed that uncertainty in the MIRs is independent of uncertainty in the estimated age-specific death rates.

Prevalence and YLD estimation

After transforming the final GBD cancer mortality estimates to incidence estimates, incidence was combined with the relative yearly survival estimates up to ten years (step 7 in "Flowchart of GBD 2017 cancer incidence, prevalence and YLD estimation"). Prior publications suggest that the value of (1 – MIR) may serve as a proxy for five-year relative survival, with the exact correlation varying slightly by cancer type.[21] We used SEER*Stat[22] to obtain mortality, incidence, and relative survival statistics from the nine SEER registries reporting from 1980–2014 (step 2 in "Flowchart of GBD 2017 cancer incidence, prevalence and YLD estimation"), by cancer type, sex, five-year blocks (ie, 1980–1984, 1985–1989, etc.), and five-year age groups (except combining 80+)[23]. For each cancer, we modelled five-year relative survival with the SEER MIRs using Poisson regression, weighted by the number of incident cases (step 3 in "Flowchart of GBD 2017 cancer incidence, prevalence and YLD estimation"). To reduce variability due to small samples, we only included MIRs based on at least 25 incident cases (except for the rarer cancers mesothelioma, nasopharyngeal cancer, and acute myeloid leukaemia, where MIRs based on at least ten cases were included). These models were then

applied to the GBD MIR estimates to predict an estimated five-year survival for each age/sex/year/location (step 4 in “Flowchart of GBD 2017 cancer incidence, prevalence, and YLD estimation”). To prevent unrealistic values, predicted survival values were winsorised to be between 100% survival and the worst-case survival scenario from SurvCan and USA 1950 survival data[24,25] utilised in previous GBD cycles. To obtain yearly survival estimates up to ten years, we compared these five-year relative survival estimates to the SEER sex-specific all-ages relative five-year survival data from 2004 (the latest year with ten-year survival available).[26] The proportion of the predicted GBD five-year survival estimate to the SEER five-year survival statistic was used as a scalar to generate yearly survival estimates from the GBD survival predictions under the proportional hazard assumption (step 5 in “Flowchart of GBD 2017 cancer incidence, prevalence and YLD estimation”). To transform relative (adjusting for background mortality) to absolute survival, GBD 2017 lifetables were used (steps 6 and 7 in “Flowchart of GBD 2017 cancer incidence, prevalence, and YLD estimation”) to calculate lambda values: lambda= (ln(nLxn/nLxn+1))/5, where nLx=person-years lived between ages x and x+n (from GBD lifetable[27]). Absolute survival was then calculated using an exponential survival function (absolute survival = relative survival * $e^{lambda*t}$).

For the purposes of calculating disability due to cancer, survivors beyond ten years were considered cured. For this group, the survivor population prevalence was divided into two sequelae (1. diagnosis and primary therapy; 2. controlled phase). For the population that did not survive beyond ten years, the yearly prevalence was divided into the four sequelae by assigning the fixed durations for each (1. diagnosis and primary therapy phase, 2. metastatic phase, 3. terminal phase, and assigning the remaining prevalence to the 4. controlled phase) (step 9 in “Flowchart of GBD 2017 cancer incidence, prevalence and YLD estimation”). Table 4 lists the duration of each of the four sequelae, along with the sources used to determine their length. For those values without a source listed, expert opinion was used to determine length.

Appendix Table 4: Duration of four prevalence sequelae by cancer

	Diagnosis/ Treatment (months)	Remission	Disseminated/metastatic (months)	Note	Terminal (months)
Oesophageal cancer	5[28]	Calculated based on remainder of time after attributing other sequelae.	4.6[26]	SEER Summary Stage 1997 (Distant site/node involved) 1995–2000	1 month
Stomach cancer	5.2[28]		3.88[26]	SEER Summary Stage 1997 (Distant site/node involved) 1995–2000	
Liver cancer	4		2.51[26]	SEER Summary Stage 1997 (Distant site/node involved) 1995–2000	
Larynx cancer	5.3[28]		8.84[26]	SEER Stage IVc	
Lung cancer	3.3[29]		4.51[26]	SEER Summary Stage 1997 (Distant site/node involved) 1995–2000	

Breast cancer	3[29]	17.7[26]	SEER Summary Stage 1997 (Distant site/node involved) 1995–2000
Cervical cancer	4.8[28]	9.21[26]	SEER Summary Stage 1997 (Distant site/node involved) 1995–2000
Uterine cancer	4.6[28]	11.6[26]	SEER Summary Stage 1997 (Distant site/node involved) 1995–2000
Prostate cancer	4[29]	30.35[26]	SEER Summary Stage 1997 (Distant site/node involved) 1995–2000
Colorectal cancer	4[29]	9.69[26]	SEER Summary Stage 1997 (Distant site/node involved) 1995–2000
Oral cancer	5.3[28]	9.33[26]	SEER Stage IVc
Nasopharyngeal cancer	5.3[28]	13.19[26]	SEER Stage IVc
Cancer of other part of pharynx	5.3[28]	7.91[26]	SEER Stage IVc
Gallbladder cancer	4	3.47[26]	SEER Summary Stage 1997 (Distant site/node involved) 1995–2000
Pancreas cancer	4.1[28]	2.54[26]	SEER Summary Stage 1997 (Distant site/node involved) 1995–2000
Melanoma	2.9[30]	7.18[26]	SEER Summary Stage 1997 (Distant site/node involved) 1995–2000
Ovarian cancer	3.2[29]	25.6[26]	SEER Summary Stage 1997 (Distant site/node involved) 1995–2000
Testicular cancer	3.7[28]	19.47[26]	SEER Stage III
Kidney cancer	5.3[28]	5.38[26]	SEER Summary Stage 1997 (Distant site/node involved) 1995–2000
Bladder cancer	5.1[28]	5.8[26]	SEER Summary Stage 1997 (Distant site/node involved) 1995–2000
Brain cancer	5	6.93[26]	SEER Median age-standardised survival, all patients, all years
Thyroid cancer	3	19.39[26]	SEER Stage IVc
Mesothelioma	4	7.75[26]	SEER Summary Stage 1997 (Distant site/node involved) 1995–2000
Hodgkin lymphoma	3.7[29]	26[31]	

Non-Hodgkin lymphoma	3.7[29]		7.7[31]		
Multiple myeloma	7[28]		36.82[26]	SEER Median age-standardised survival, all patients, all years	
Leukaemia	5[28]		43.67[26]	SEER Median age-standardised survival, all patients, all years	
ALL	12		7.02[26]	SEER Median age-standardised survival, all patients, all years	
AML	6		4.6[26]	SEER Median age-standardised survival, all patients, all years	
CLL	6		48[32]	SEER Median age-standardised survival, all patients, all years	
CML	6		4.6[26]	SEER Median age-standardised survival for AML (patients with CML die in blast crisis, which is treated like AML), all patients, all years	
Leukaemia other	6		48[32]	SEER Median age-standardised survival, all patients, all years	
Other	4.4 (mean of other cancer durations)		15.81[26]	SEER Median age-standardised survival, all patients, all years	

For cancer-specific procedure sequelae, hospital data were used to estimate the number of cancer patients undergoing mastectomy, laryngectomy, stoma, prostatectomy, and cystectomy (step 10 in "Flowchart of GBD 2017 cancer incidence, prevalence, and YLD estimation"). Proportions were generated by dividing the rate of procedures generated from the diagnostic codes in the hospital dataset and the coverage population by the GBD age-, and sex-specific disease incidence rates for that country. Diagnostic codes used are listed in Appendix Table 5 below.

Appendix Table 5: Procedure codes used to estimate cancer procedure proportions

Procedure	Cancer	Procedure code (ICD-9_CM)
Mastectomy	Breast cancer	854, 8541, 8542, 8543, 8544, 8545<br>8546, 8547, 8548
Laryngectomy	Larynx cancer	301, 303, 304, 3029
Stoma	Colon and rectum cancer	461, 4610, 4611, 4613, 4862
Cystectomy	Bladder cancer	5771, 5779
Prostatectomy	Prostate	603, 604, 605, 606, 6062

To estimate procedure-related disability for each of these five cancers, the procedure proportions (proportion of each cancer population that undergo these procedures) from hospital data were used as input for a proportion model in DisMod-MR 2.1 to estimate the proportions for all locations, by age, year, and by sex.

Since colostomy or ileostomy procedures are done for reasons other than cancer, a literature review was conducted to determine the proportion of ostomies due to colorectal cancer. The "all-cause" colostomy proportions were multiplied by 0.58, based on the results of the literature review that an average of 58% of ostomies are done for colorectal cancer.[33-35]

The final procedure proportions were applied to the incidence cases of the respective cancers and multiplied with the proportion of the incidence population surviving for ten years to determine the incident cases of the cancer population that underwent procedures and that survived beyond ten years. These incident cases were used again as an input for DisMod-MR 2.1, with a remission specification of zero and an excess mortality rate prior of 0 to 0.1, as well as with increasing the age of the population and the year by ten years to reflect prevalence after that population has survived ten years (See Section 2.3 in Supplement 1 to: "Global, regional, and national incidence, prevalence, and years lived with disability for 354 diseases and injuries for 195 countries and territories, 1990–2017: a systematic analysis for the Global Burden of Disease Study 2017" for a more complete description of DisMod-MR 2.1).[5] The results from this model are incidence and lifetime prevalent cases of persons with these cancer-related sequelae who have survived beyond ten years.

Since disability associated with prostatectomy comes from impotence and incontinence, and not from the prostatectomy itself, 18% of the prostatectomy prevalence was assumed to have incontinence and 55% was assumed to have impotence, based on a literature review done for GBD 2013.[36-43] Cases were assigned disability for either impotence or incontinence, but no cases were assigned disability from both.

We assumed that for the population surviving up to ten years, only the prevalence population being in remission experiences additional disability due to procedures (eg, women suffering from metastatic breast cancer do not experience additional disability due to a mastectomy during this phase). To estimate the prevalence of the cancer population in remission during the first ten years after diagnosis with and without procedure-related disability, we multiplied the prevalence of the population in the remission phase with the proportion of the population undergoing a procedure. This step allowed us to estimate disability during the remission phase for both the population experiencing disability due to the remission phase alone, as well as the population experiencing disability from the remission phase and the additional procedure-related disability.
Lastly, the procedure sequelae prevalence and general sequelae prevalence were multiplied with their respective disability weights (Appendix Table 6) to obtain the number of YLDs (steps 11 and 12 in "Flowchart of GBD 2017 cancer incidence, prevalence and YLD estimation"). Further description of disability weights can be found in Section 2.5 and 2.6 in "Global, regional, and national incidence, prevalence, and years lived with disability for 354 diseases and injuries for 195 countries and territories, 1990–2017: a systematic analysis for the Global Burden of Disease Study 2017".[5] The sum of these YLDs is the final YLD estimate associated with each cancer.

Appendix Table 6: Lay description of cancer states and corresponding disability weights			
**Health state**	**Lay description**	**Estimate**	**95% Uncertainty interval**
Cancer, diagnosis and primary therapy (cancer_diagnosis)	This person has pain, nausea, fatigue, weight loss, and high anxiety.	0.288	0.193-0.399
Cancer, controlled phase (generic_medication)	This person has a chronic disease that requires medication every day and causes some worry but minimal interference with daily activities.	0.049	0.031-0.072
Cancer, metastatic (cancer_metastatic)	This person has severe pain, extreme fatigue, weight loss, and high anxiety.	0.451	0.307-0.600
Terminal phase, with medication (cancer_terminal_treat)	This person has lost a lot of weight and regularly uses strong medication to avoid constant pain. The person has no appetite, feels nauseated, and needs to spend most of the day in bed.	0.540	0.377-0.687
Mastectomy (cancer_mastectomy)	This person had one of her breasts removed and sometimes has pain or swelling in the arms.	0.036	0.020-0.057
Stoma (cancer_stoma)	This person has a pouch attached to an opening in the belly to collect and empty stools.	0.095	0.063-0.131
Laryngectomy (speech_problems)	This person has difficulty speaking, and others find it difficult to understand.	0.051	0.032-0.078
Urinary incontinence (incontinence)	This person cannot control urinating.	0.139	0.094-0.198
Impotence (impotence)	This person has difficulty in obtaining or maintaining an erection.	0.017	0.009-0.030

Uncertainty Estimation

Uncertainty in cancer estimates begins with the availability of and variability in cancer cause-specific data by age, sex, location and year. The uncertainty in cancer mortality estimates arises from CODEm and CoD Correct. For more information see this appendix page 45, the CODEm methodology paper by Foreman et al., and Supplement 1 to the GBD 2017 cause of death capstone.[3,4] Uncertainty in cancer incidence estimates results from both the uncertainty in mortality estimates as well as the uncertainty in the MIR estimates, which result from the ST-GPR models (see appendix p 40). Uncertainty from the mortality estimates and the MIRs were assumed to be independent.

Cancer prevalence uncertainty results from both the incidence uncertainty as well as the uncertainty from survival estimates. These were assumed to be independent. Uncertainty in cancer YLD estimation results from the uncertainty in the prevalence of each cancer sequela and

uncertainty in the disability weight and is propagated into the final comorbidity-corrected YLD result. The uncertainty in prevalence and the uncertainty in disability weights are assumed to have no correlation. Cancer YLL uncertainty results from uncertainty in mortality estimates as well as uncertainty in life expectancy estimates. Uncertainty in cancer DALY estimates results from the uncertainty in YLLs and the uncertainty in YLDs, which were assumed to be independent.[44]

The same technique for propagating uncertainty elsewhere in the GBD study is applied in the cancer estimation process. In brief, the distribution of each step in the computation process is stored in 1000 draws. The distributions are determined from the data input sampling error, the uncertainty of the model coefficients, and the uncertainty of severity distributions and disability weights. The 1000 draws are used for every step in the process, with final estimates computed using the mean estimate across 1000 draws. The 95% uncertainty intervals are determined by the 25th and 975th ranked values across all 1000 draws.[5] More specific information regarding uncertainty intervals can be found in the GBD 2017 capstone papers.[4,5,44,45]

Additional GBD 2017 study Methodology

GBD 2017 ST-GPR Methodology Overview

This section was copied from Supplement 1 to "GBD 2017 Risk Factor Collaborators. Global, regional, and national comparative risk assessment of 84 behavioural, environmental and occupational, and metabolic risks or clusters of risks for 195 countries and territories, 1990–2017: a systematic analysis for the Global Burden of Disease Study 2017. *Lancet* 2018; **392**: 1923–45." It is included in this supplementary appendix for convenience to our readers. References in the original supplement are updated to be correctly cited within this supplement.

Spatiotemporal Gaussian process regression

Spatiotemporal Gaussian process regression (ST-GPR) has been used for risk factors where the data density is sufficient to estimate a very flexible time trend. The flowchart showing the analytic steps can be found in Appendix Figure 3 [in Supplement 1 to the GBD 2017 Risk Factor capstone]. The approach is a stochastic modelling technique that is designed to detect signals amidst noisy data. It also serves as a powerful tool for interpolating non-linear trends.[46,47] Unlike classical linear models that assume that the trend underlying data follows a definitive functional form, GPR assumes that the specific trend of interest follows a Gaussian Process, which is defined by a mean function $m(\cdot)$ and a covariance function $Cov(\cdot)$. For example, let $p_{c,a,s,t}$ be the exposure, in normal, log, or logit space, observed in country *c*, for age group a, and sex *s* at time t:

$$\left(p_{c,a,s,t}\right) = g_{c,a,s}(t) + \epsilon_{c,a,s,t}$$

where

$$\epsilon_{c,a,s,t} \sim Normal\left(0, \sigma_p^2\right),$$
$$g_{c,a,s}(t) \sim GP\left(m_{c,a,s}(t), Cov\left(g_{c,a,s}(t)\right)\right).$$

The derivation of the mean and covariance functions, $m_{c,a,s}(t)$ and $Cov\left(g_{c,a,s}(t)\right)$, along with a more detailed description of the error variance (σ_p^2), is described below.

Estimating mean functions
We estimated mean functions using a two-step approach. To be more specific, $m_{c,a,s}(t)$ can be expressed, depending on the exposure transformation, as:

$$log\left(p_{c,a,s}(t)\right) = X_{c,a,s}\beta + h(r_{c,a,s,t})$$

$$logit\left(p_{c,a,s}(t)\right) = X_{c,a,s}\beta + h(r_{c,a,s,t})$$

$$p_{c,a,s}(t) = X_{c,a,s}\beta + h(r_{c,a,s,t})$$

where $X\beta$ is the summation of the components of a hierarchical mixed-effects linear regression, including the intercept and the product of covariates with their corresponding fixed effect coefficients. Some models were run as hierarchical mixed-effects linear regressions, with random effects on the levels of the geographic hierarchy. For most mixed-effects models, random effects were only used in the fit, not in the prediction. The second part of the equation, $h(r_{c,a,s,t})$, is a smoothing function for the residuals, $r_{c,a,s,t}$, derived from the linear model.[48] Descriptions of exposure transformations and which covariates were used in linear models can be found in Appendix Section 4 [in Supplement 1 to the GBD 2017 Risk Factor capstone], which described the risk-specific estimation approaches. Some models used a custom stage-1 estimate – these risks will have detailed information on their mixed-effect estimation process in the risk-specific appendix sections.

While the linear component captures the general trend in exposures over time, much of the data varaibility may still not be adequately accounted for. To address this, we fit a locally weighted polynomial regression (LOESS) function $h(r_{c,a,s,t})$ to systematically estimate this residual variability by borrowing strength across time, age, and space patterns (the spatiotemporal component of ST-GPR).[49,50] The time adjustment parameter, defined by λ , aims to borrow strength from neighboring time points (i.e. the exposure in this year is highly correlated with exposure in the previous year but less so further back in time). The age adjustment parameter, defined by ω, borrows strength from data in neighboring age groups. The space adjustment parameter, defined by ξ, aims to borrow strength across the hierarchy of geographical locations. This year, we further combined the spatial and temporal weights into a single space-time weight, to allow the amount of spatial weight given to a particular point $r_{c,a,s,t}$ to fluctuate given the data availability at each time *t* and location-level l in the location hierarchy.

Let $w_{c,a,s,t}$ be the final weight assigned to observation $r_{c,a,s,t}$ with reference to a focal observation r_{c_0,a_0,s_0,t_0}. We first generated a temporal weight $t.w_{c,a,s,t}$ for smoothing over time, which was based on the scaled distance along the time dimension of the two observations[49]:

$$t.w_{c,a,s,t} = \frac{1}{e^{\lambda|t-t_0|}}$$

Next, we generated a spatial weight to smooth over geography. Specifically, we defined a geospatial relationship by categorizing data based on the GBD location hierarchy (Appendix Table 7 [in Supplement 1 to the GBD 2017 Risk Factor capstone]).

In previous GBD iterations, a vector of spatial weights corresponding to each level of the location hierarchy was derived as $[\xi, \xi * (1-\xi)^{n_1-1}, \ldots, \xi * (1-\xi)^{n_i-1}, (1-\xi)^{n_i}]$, where n_i, designated the number of location levels in between the given location and the global level and ζ was typically between .7 - .99. Under the previous spatial weighting system, all country datapoints would receive a weight of ζ, all regional datapoints a weight of ζ *(1- ζ), etc, no matter how much data was available in the country compared to the region. For example, if there was only a single datapoint for a given country and ζ was set to .7 , that lone datapoint would receive 70% of the spatial weight.

This year, we reformulated zeta to act as a scalar on a given datapoint given its proximity to the target location:

$$t.w_{c,a,s,t} = \zeta^{|c-c_0|}$$

For example, estimating a country would use the following weighting scheme:

- Country data: ζ^0 = 1
- Regional data not from the country being estimated: ζ^1
- Data from other regions in the same super region: ξ^2
- Global data from other super regions: ζ^3

Under the new spatial weighting specification, typical values of ζ range from [.001, .2], where ζ can be interpreted as the amount to downweight regional datapoints compared to country datapoints for a given estimating country. For example, for a given datapoint $r_{c,a,s,t}$ and ζ = .01, a datapoint not within country *c* but within the same region *r* as $r_{c,a,s,t}$ would be assigned $\frac{1}{100}$ the weight of a datapoint within the country.

The spatial and temporal weights were then multiplied and summed across each level of the location hierarchy, and normalized for each time period *t* . This allows the space-time weight to implicitly take into account the amount of data available at the country vs. region vs super-region level and attribute spatial weight accordingly.

Given a normalization constant,

$$K_i = \sum_{c \epsilon C} s.w_{c,t} * t.w_{c.t} + \sum_{c \epsilon R} s.w_{c,t} * t.w_{c.t} + \sum_{c \epsilon SR} s.w_{c,t} * t.w_{c.t}$$

the final space-time weight would then equal

$$w'_{c,a,s,t} = \frac{s.w_{c,t} * t.w_{c,t}}{K_i}$$

Finally, we calculated the weight $w''_{c,a,s,t}$ to smooth over age, which is based on a distance along the age dimension of two observations. For a point between the age a of the observation $r_{c,a,s,t}$ and a focal observation r_{c_0,a_0,s_0,t_0}, the weight is defined as follows:

$$w''_{c,a,s,t} = \frac{1}{e^{\omega|a-a_0|}}$$

The final weights would then be computed by simply multiplying the space-time weights and age weights and normalizing so all weights for a given time period *t* sum to 1. A full derivation of weights for each category follow, assuming the location being estimated was a country, follows:

1) If the observation $r_{c,t}$ belongs to the same country c_0 of the focal observation r_{c_0,t_0}:

$$w_{c,a,s,t} = \frac{(w'_{c,a,s,t} w''_{c,a,s,t})}{\sum_{c=c_0}(w'_{c,a,s,t} w''_{c,a,s,t})} \qquad \forall c = c_0$$

2) If the observation $r_{c,t}$ belongs to a different country than the focal observation r_{c_0,t_0}, but both belong to the same region R:

$$w_{c,a,s,t} = \frac{(w'_{c,a,s,t} w''_{c,a,s,t})}{\sum_{c\neq c_0}(w'_{c,a,s,t} w''_{c,a,s,t})} \qquad \forall c \neq c_0 \cap R[c] = R[c_0]$$

3) If the observation $r_{c,t}$ belongs to the same super region SR but to a both different country c_0 and region $R[c_0]$ than the focal observation r_{c_0,t_0}:

$$w_{c,a,s,t} = \frac{(w'_{c,a,s,t} w''_{c,a,s,t})}{\sum_{c\neq c_0}(w'_{c,a,s,t} w''_{c,a,s,t})} \qquad \forall c \neq c_0 \cap R[c] \neq R[c_0] \cap SR[c] = SR[c_0]$$

4) If the observation $r_{c,t}$ is from a different super region than the focal observation r_{c_0,t_0}(ie. all other data currently not receiving a weight):

$$w_{c,a,s,t} = \frac{(w'_{c,a,s,t} w''_{c,a,s,t})}{\sum_{c\neq c_0}(w'_{c,a,s,t} w''_{c,a,s,t})} \qquad \forall c \neq c_0 \cap R[c] \neq R[c_0] \cap SR[c] \neq SR[c_0]$$

Observations could be downweighted by a factor of 0.1, usually because they were not geographically representative at the unit of estimation. Details of reasons for downweighting can be found in risk-specific modeling summaries. The final weights were then normalized such that the sum of weights across age, time, and geographic hierarchy for a reference group was 1.

Estimating error variance

σ_p^2 represents the error variance in normal or transformed space including sampling variance of the estimates and prediction error from any crosswalks performed. First, variance was systematically imputed if the data extraction did not include any measure of uncertainty. When some sample sizes for data were available, missing sample sizes were imputed as the 5th percentile of available sample sizes. Missing variances were then calculated as $\sigma_p^2 = \frac{p*(1-p)}{n}$ for proportions or were predicted from the mean using a regression for continuous values. When sample sizes were entirely missing and could not be imputed, the 95th percentile of available variances at the most granular geographic level (ie, first country, then region, etc.) were used to impute missing variances. For proportions where *p*n* or *(1-p)*n* is < 20, variance was replaced using the Wilson Interval Score method.

Next, if the exposure was modelled as a log transformation, the error variance was transformed into log-space using the delta method approximation as follows,

$$\sigma_p^2 \cong \frac{\sigma_{p'}^2}{p_{c,a,s,t}^2}$$

where $\sigma_{p'}^2$ represents the error variance in normal space. If the exposure was modelled as a logit transformation, the error variance was transformed into logit-space using the delta method approximation as follows,

$$\sigma_p^2 \cong \frac{\sigma_{p'}^2}{(p_{c,a,s,t} * (1 - p_{c,a,s,t}))^2}$$

Finally, prior to GPR, an approximation of non-sampling variance was added to the error variance. Calculations of non-sampling variance were performed on normal-space variances. Non-sampling variance was calculated as the variance of inverse-variance weighted residuals from the space-time estimate at a given location level hierarchy. If there were fewer than 10 data points at a given level of the location hierarchy the non-sampling variance was replaced with that of the next highest geography level with more than 10 data points.

Estimating the covariance function

The final input into GPR is the covariance function, which defines the shape and distribution of the trends. Here, we have chosen the Matern-Euclidian covariance function, which offers the flexibility to model a wide spectrum of trends with varying degrees of smoothness. The function is defined as follows:

$$M(t,t') = \sigma^2 \frac{2^{1-v}}{\Gamma(v)} \left(\frac{d(t,t')\sqrt{2v}}{l}\right)^v K_v\left(\frac{d(t,t')\sqrt{2v}}{l}\right)$$

where $d(\cdot)$is a distance function; $\sigma^2,\ v,\ l$, and K_v are hyperparameters of the covariance function—specifically σ^2 is the marginal variance, v is the smoothness parameter that defines the differentiability of the function, l is the length scale, which roughly defines the distance between which two points become uncorrelated, and K_v is the Bessel function. We approximated σ^2 by taking the normalized median absolute deviation $MADN(r_c')$ of the difference which is the normalized absolute deviation of the difference of the first-stage linear regression estimate from the second-stage spatiotemporal smoothing step for each country. We then took the mean of these country-level MADN estimates for all countries with 10+ country-years of data, to ensure that differences between first- and second-stage estimates had sufficient data to truly convey meaningful information on model uncertainty. We used the parameter specifications $v = 2$ for all models. The scale parameter l used for each risk is reported in Appendix Section 4 [in Supplement 1 to the GBD 2017 Risk Factor capstone].

Prediction using GPR

We integrated over $g_{c,t}(t_*)$ to predict a full time series for country c, age a, sex s, and the prediction time t_*:

$$p_{c,a,s}(t_*) \sim N\left(m_{c,a,s,t}(t_*), \sigma_p^2 I + Cov\left(g_{c,a,s,t}(t_*)\right)\right)$$

Random draws of 1000 samples were obtained from the distributions above for every country for a given indicator. The final estimated mean for each country was the mean of the draws. In

addition, 95% uncertainty intervals were calculated by taking the 2.5 and 97.5 percentile of the sample distribution. The linear modelling process was implemented using the lmer4 package in R, and the ST-GPR analysis was implemented through the PyMC2 package in Python.

GBD 2017 CODEm Methodology Overview

This section was copied from "Section 3.1: CODEm" in Supplement 1 to Roth GA, GBD 2017 Causes of Death Collaborators, Abate D, et al. Global, regional, and national age-sex-specific mortality for 282 causes of death in 195 countries and territories, 1980-2017: a systematic analysis for the Global Burden of Disease Study 2017. *Lancet* 2018; **392**(10159): 1736-88. It is included in this supplementary appendix for convenience to our readers. References in the original supplement are updated to be correctly cited within this supplement.

Section 3.1.1: Overview of methods

CoD ensemble modelling (CODEm) is the framework used to model most cause-specific death rates in the GBD.[3] It relies on four key components:

First, all available data are identified and gathered to be used in the modelling process. Though the data may vary in quality, they all contain some signal of the true epidemiological process.

Second, a diverse set of plausible models are developed to capture well-documented associations in the estimates. Using a wide variety of individual models to create an ensemble predictive model has been shown to outperform techniques using only a single model both in CoD estimation[3] and in more general prediction applications.[51,52]

Third, the out-of-sample predictive validity is assessed for all individual models, which are then ranked for use in the ensemble modelling stage.

Finally, differently weighted combinations of individual models are evaluated to select the ensemble model with the highest out-of-sample predictive validity.

For some causes (see, for example, "lower respiratory infections"), there is evidence that the relationship between covariates and death rates might differ between children and adults. Separate models are therefore run for different age ranges, when applicable. Additionally, separate models are developed for countries with extensive, complete, and representative VR for every cause to ensure that uncertainty can better reflect the more complete data in these locations.

Section 3.1.2: Model pool development

Because many factors may covary with any given CoD, a range of plausible statistical models are developed for each cause. In the CODEm framework, four families of statistical models are used: linear mixed effects regression (LMER) models of the natural log of the cause-specific death rate, LMER models of the logit of the cause fraction, spatiotemporal Gaussian process regression (ST-GPR) models of the natural logarithm of the cause-specific death rate, and ST GPR models of the logit of the cause fraction (see the 2x2 table in Foreman et al).[3] For each family of models, all plausible relationships between covariates and the response variable are identified. Because all possible combinations of selected covariates are considered for each

family of models, multicollinearity between covariates may produce implausible signs on coefficients or unstable coefficients. Each combination is therefore tested for statistical significance (covariate coefficients must have a coefficient with p-value < 0·05) and plausibility (the coefficients must have the directions expected based on the literature). Only covariate combinations meeting these criteria are retained. This selection process is run for both cause fractions and death rates, then ST-GPR and LMER-only models are created for each set of covariates. For a detailed explanation of the covariate selection algorithm, see Foreman et al 2012.[3]

Section 3.1.3: Data Variance Estimation

The families of models that go through ST-GPR described in Section 3.1.2 incorporate information about data variance. The main inputs for a Gaussian process regression (GPR) are a mean function, a covariance function, and data variance for each data point. These inputs are described in detail in Foreman et al 2012.[3] For GBD 2017, we have updated this calculation to incorporate garbage code redistribution uncertainty.

There are now three components of data variance used in CODEm: sampling variance, non-sampling variance, and garbage code redistribution variance. The computation of sampling variance and nonsampling variance have not changed since previous iterations of the GBD and are also described in Foreman et al 2012.[3] Garbage code redistribution variance is computed in the cause of death database process described in Section 2.7 of [Supplement 1 to the GBD 2017 cause of death capstone[4]]. Since variance is additive, we calculate total data variance as the sum of sampling variance, non-sampling variance, and redistribution variance. Increased data variance in GPR results in the GPR draws not following the data point as closely.

Section 3.1.4: Testing model pool on 15% sample

The performance of all models (individual and ensemble) is evaluated using out-of-sample predictive validity tests. Thirty percent of the data are excluded from the initial model fits. These individual model fits are evaluated and ranked using half of the excluded data (15% of the total), then used to construct the ensembles based on their performance. Data are held out from the analysis based on the cause-specific missingness patterns for ages and years across locations. Out-of-sample predictive validity testing is repeated 20 times for each model, which has been shown to produce stable results.[3] These performance tests include the root mean square error (RMSE) for the log of the causespecific death rate, the direction of the predicted versus actual trend in the data, and the coverage of the predicted 95% UI.

Section 3.1.5: Ensemble development and testing

The component models are weighted based on their predictive validity rank in order to determine their contribution to the ensemble estimate. The relative weights are determined both by the model ranks and by a parameter ψ, whose value determines how quickly the weights taper off as rank decreases. The distribution of ψ is described in more detail in Foreman et al 2012.[3] A set of ensemble models is then created using the weights constructed from the combinations of ranks and ψ values. These ensembles are tested using the predictive validity metrics described in Section 3.1.3 on the remaining 15% of the data, and the ensemble with the best performance in out-of-sample trend and RMSE is chosen as the final model.

Section 3.1.6: Final Estimation
Once a weighting scheme has been chosen, 1,000 draws are created for the final ensemble, with the number of draws contributed by each model proportional to its weight. The mean of the draws is used as the final estimate for the CODEm process, and a 95% uncertainty interval (UI) is created from the 0·025 and 0·975 quantiles of the draws. The validity of the UI can be checked via its coverage of the out-of-sample data; ideally, the 95% UI would capture 95% of these data. Higher coverage suggests that the UIs are too large, and lower coverage suggests overfitting.

Section 3.1.7: Selection of causes for which CODEm is used
CODEm is used to model 193 causes, described in detail below. However, it is unsuitable for use in modelling certain causes, including those with very low death counts, those where cause specific death record availability is inadequate, or those for which there are marked biases or variability for CoD certification over time that cannot be fully accounted for with the current garbage code redistribution algorithms. Criteria for causes where CODEm is not used are discussed in further detail in Section 3.2 [of Supplement 1 to the GBD 2017 cause of death capstone[4]].

Section 3.1.8: Model-specific covariates
A table of CODEm covariates used, level of the covariate, and expected direction of the covariate by cause, sex, age, and location can be found in Appendix Table 9 [of Supplement 1 to the GBD 2017 cause of death capstone[4]]. Modelers select covariates to be used in CODEm, but those covariates may not be significant or in the direction specified during the covariate selection step of CODEm, and will therefore not be used in the model. These covariates are listed with a '—' for number of draws. Additionally, covariates may be selected by CODEm, but only exist in submodels that perform poorly, and may end up with zero draws included in the final ensemble. Finally, all other covariates are listed with the number of draws in the final ensemble from submodels that had the covariate. A comparison of GBD 2016 and GBD 2017 covariates using CoD modeling is provided in Appendix Table 10 [of Supplement 1 to the GBD 2017 cause of death capstone[4]].

Section 3.1.9: Fit statistics for CODEm models
A table of CODEm predictive validity results by cause, sex, and, and location can be found in Appendix Table 11 [of Supplement 1 to the GBD 2017 cause of death capstone[4]].

Socio-demographic Index (SDI) Definition and Calculation
Socio-demographic Index (SDI) is the geometric mean of three rescaled components: (1) total fertility rate under age 25 years (ie, the number of births expected per woman aged 10–24 years), (2) lag-distributed income per capita, and (3) average educational attainment in populations aged 15 years or older.[44] SDI scores are scaled from 0 to 1, with 0 representing the "lowest income, fewest years of schooling, and highest fertility" and 1 representing the "highest income, most years of schooling, and lowest fertility."[44] See Section 2.3 in Supplement 1 to the GBD 2017 DALY capstone[44] for more details regarding SDI estimation, and page 48 of this appendix for the SDI quintile estimate for each country in GBD 2017.

Additional Methodology Tables and Figures

Appendix Table 7: SDI quintile for countries estimated in GBD 2017

Country name	SDI quintile
Afghanistan	Low SDI
Albania	Middle SDI
Algeria	Middle SDI
American Samoa	High-middle SDI
Andorra	High SDI
Angola	Low-middle SDI
Antigua and Barbuda	High-middle SDI
Argentina	High-middle SDI
Armenia	High-middle SDI
Australia	High SDI
Austria	High SDI
Azerbaijan	High-middle SDI
Bahrain	High-middle SDI
Bangladesh	Low SDI
Barbados	High-middle SDI
Belarus	High-middle SDI
Belgium	High SDI
Belize	Low-middle SDI
Benin	Low SDI
Bermuda	High-middle SDI
Bhutan	Low-middle SDI
Bolivia	Low-middle SDI
Bosnia and Herzegovina	High-middle SDI
Botswana	Middle SDI
Brazil	Middle SDI
Brunei	High SDI
Bulgaria	High-middle SDI
Burkina Faso	Low SDI
Burundi	Low SDI
Cambodia	Low-middle SDI
Cameroon	Low-middle SDI
Canada	High SDI
Cape Verde	Low-middle SDI
Central African Republic	Low SDI
Chad	Low SDI

Chile	High-middle SDI
China	High-middle SDI
Colombia	Middle SDI
Comoros	Low SDI
Congo	Low-middle SDI
Costa Rica	Middle SDI
Côte d'Ivoire	Low SDI
Croatia	High SDI
Cuba	Middle SDI
Cyprus	High SDI
Czech Republic	High SDI
Democratic Republic of the Congo	Low SDI
Denmark	High SDI
Djibouti	Low-middle SDI
Dominica	Middle SDI
Dominican Republic	Low-middle SDI
Ecuador	Middle SDI
Egypt	Low-middle SDI
El Salvador	Low-middle SDI
Equatorial Guinea	Middle SDI
Eritrea	Low SDI
Estonia	High SDI
Ethiopia	Low SDI
Federated States of Micronesia	Low-middle SDI
Fiji	Middle SDI
Finland	High SDI
France	High SDI
Gabon	Middle SDI
Georgia	High-middle SDI
Germany	High SDI
Ghana	Low-middle SDI
Greece	High SDI
Greenland	High-middle SDI
Grenada	Middle SDI
Guam	High-middle SDI
Guatemala	Low-middle SDI
Guinea	Low SDI
Guinea-Bissau	Low SDI
Guyana	Low-middle SDI

Haiti	Low SDI
Honduras	Low-middle SDI
Hungary	High-middle SDI
Iceland	High SDI
India	Low-middle SDI
Indonesia	Middle SDI
Iran	High-middle SDI
Iraq	Low-middle SDI
Ireland	High SDI
Israel	High-middle SDI
Italy	High SDI
Jamaica	Middle SDI
Japan	High SDI
Jordan	Middle SDI
Kazakhstan	High-middle SDI
Kenya	Low-middle SDI
Kiribati	Low SDI
Kuwait	High-middle SDI
Kyrgyzstan	Low-middle SDI
Laos	Low-middle SDI
Latvia	High SDI
Lebanon	High-middle SDI
Lesotho	Low-middle SDI
Liberia	Low SDI
Libya	High-middle SDI
Lithuania	High SDI
Luxembourg	High SDI
Macedonia	High-middle SDI
Madagascar	Low SDI
Malawi	Low SDI
Malaysia	High-middle SDI
Maldives	Middle SDI
Mali	Low SDI
Malta	High SDI
Marshall Islands	Low-middle SDI
Mauritania	Low-middle SDI
Mauritius	High-middle SDI
Mexico	Middle SDI
Moldova	Middle SDI

Mongolia	Middle SDI
Montenegro	High-middle SDI
Morocco	Low-middle SDI
Mozambique	Low SDI
Myanmar	Low-middle SDI
Namibia	Middle SDI
Nepal	Low SDI
Netherlands	High SDI
New Zealand	High SDI
Nicaragua	Low-middle SDI
Niger	Low SDI
Nigeria	Low-middle SDI
North Korea	Low-middle SDI
Northern Mariana Islands	High-middle SDI
Norway	High SDI
Oman	High-middle SDI
Pakistan	Low-middle SDI
Palestine	Low-middle SDI
Panama	Middle SDI
Papua New Guinea	Low SDI
Paraguay	Middle SDI
Peru	Middle SDI
Philippines	Middle SDI
Poland	High SDI
Portugal	High-middle SDI
Puerto Rico	High-middle SDI
Qatar	High-middle SDI
Romania	High-middle SDI
Russia	High-middle SDI
Rwanda	Low SDI
Saint Lucia	Middle SDI
Saint Vincent and the Grenadines	Middle SDI
Samoa	Low-middle SDI
São Tomé and Príncipe	Low-middle SDI
Saudi Arabia	High-middle SDI
Senegal	Low SDI
Serbia	High-middle SDI
Seychelles	Middle SDI
Sierra Leone	Low SDI

Singapore	High SDI
Slovakia	High SDI
Slovenia	High SDI
Solomon Islands	Low SDI
Somalia	Low SDI
South Africa	Middle SDI
South Korea	High SDI
South Sudan	Low SDI
Spain	High SDI
Sri Lanka	Middle SDI
Sudan	Low-middle SDI
Suriname	Middle SDI
Swaziland	Low-middle SDI
Sweden	High SDI
Switzerland	High SDI
Syria	Middle SDI
Taiwan (province of China)	High SDI
Tajikistan	Low-middle SDI
Tanzania	Low SDI
Thailand	Middle SDI
The Bahamas	High-middle SDI
The Gambia	Low SDI
Timor-Leste	Low-middle SDI
Togo	Low SDI
Tonga	Middle SDI
Trinidad and Tobago	Middle SDI
Tunisia	Middle SDI
Turkey	High-middle SDI
Turkmenistan	Middle SDI
Uganda	Low SDI
Ukraine	High-middle SDI
United Arab Emirates	High-middle SDI
United Kingdom	High SDI
United States	High SDI
Uruguay	High-middle SDI
Uzbekistan	Middle SDI
Vanuatu	Low-middle SDI
Venezuela	Middle SDI
Vietnam	Middle SDI

Virgin Islands, US	High-middle SDI
Yemen	Low SDI
Zambia	Low-middle SDI
Zimbabwe	Low-middle SDI

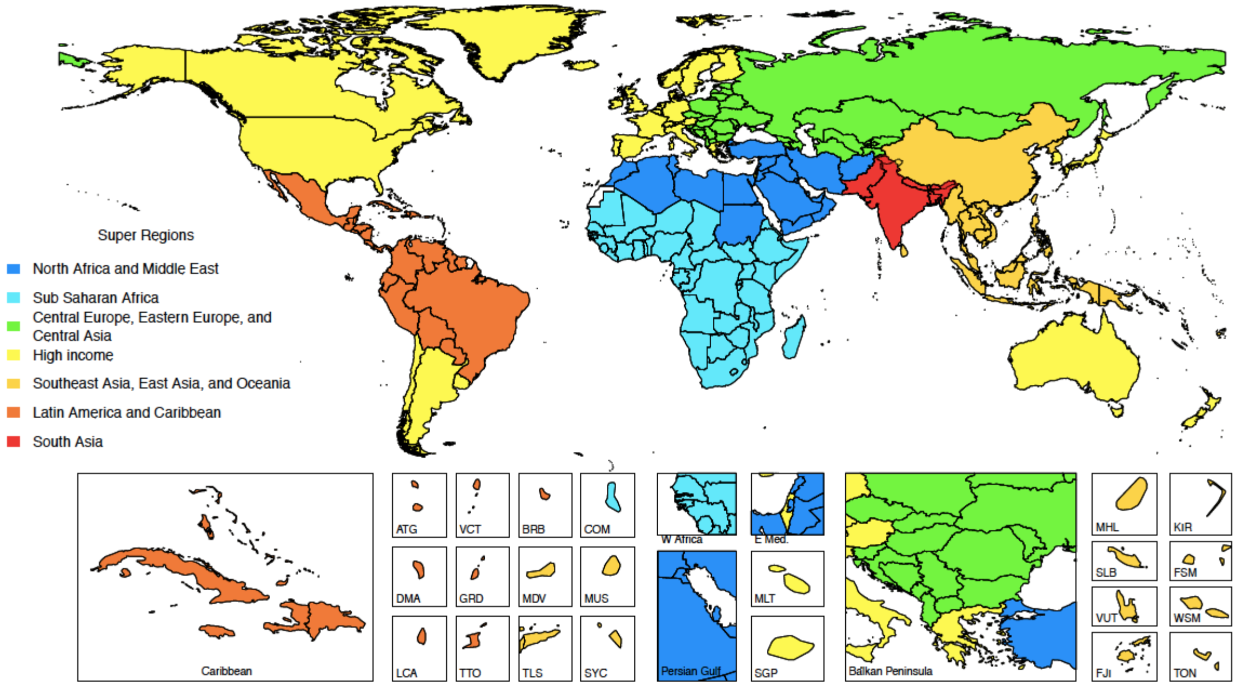

Appendix Figure 4: Map of GBD world super-regions, 2017. ATG: Antigua and Barbuda; VCT: Saint Vincent and the Grenadines; BRB: Barbados; COM: Comoros; W Africa: West Africa; E Med: Eastern Mediterranean; MHL: Marshall Islands; KIR: Kiribati; DMA: Dominica; GRD: Grenada; MDV: Maldives; MUS: Mauritius; MLT: Malta; SLB: Solomon Islands; FSM: Federated States of Micronesia; LCA: Saint Lucia; TTO: Trinidad and Tobago; TLS: Timor-Leste; SYC: Seychelles; SGP: Singapore; VUT: Vanuatu; WSM: Samoa; FJI: Fiji; TON: Tonga.

Appendix Table 8: Definition of GBD world super-regions, 2017

**Country name**	**GBD world super-region**
Afghanistan	North Africa and Middle East
Albania	Central Europe, Eastern Europe, and Central Asia
Algeria	North Africa and Middle East
American Samoa	Southeast Asia, East Asia, and Oceania
Andorra	High-income
Angola	Sub-Saharan Africa
Antigua and Barbuda	Latin America and Caribbean
Argentina	High-income
Armenia	Central Europe, Eastern Europe, and Central Asia
Australia	High-income
Austria	High-income
Azerbaijan	Central Europe, Eastern Europe, and Central Asia
The Bahamas	Latin America and Caribbean
Bahrain	North Africa and Middle East

Bangladesh	South Asia
Barbados	Latin America and Caribbean
Belarus	Central Europe, Eastern Europe, and Central Asia
Belgium	High-income
Belize	Latin America and Caribbean
Benin	Sub-Saharan Africa
Bermuda	Latin America and Caribbean
Bhutan	South Asia
Bolivia	Latin America and Caribbean
Bosnia and Herzegovina	Central Europe, Eastern Europe, and Central Asia
Botswana	Sub-Saharan Africa
Brazil	Latin America and Caribbean
Brunei	High-income
Bulgaria	Central Europe, Eastern Europe, and Central Asia
Burkina Faso	Sub-Saharan Africa
Burundi	Sub-Saharan Africa
Cambodia	Southeast Asia, East Asia, and Oceania
Cameroon	Sub-Saharan Africa
Canada	High-income
Cape Verde	Sub-Saharan Africa
Central African Republic	Sub-Saharan Africa
Chad	Sub-Saharan Africa
Chile	High-income
China	Southeast Asia, East Asia, and Oceania
Colombia	Latin America and Caribbean
Comoros	Sub-Saharan Africa
Congo	Sub-Saharan Africa
Costa Rica	Latin America and Caribbean
Côte d'Ivoire	Sub-Saharan Africa
Croatia	Central Europe, Eastern Europe, and Central Asia
Cuba	Latin America and Caribbean
Cyprus	High-income
Czech Republic	Central Europe, Eastern Europe, and Central Asia
Democratic Republic of the Congo	Sub-Saharan Africa
Denmark	High-income
Djibouti	Sub-Saharan Africa
Dominica	Latin America and Caribbean
Dominican Republic	Latin America and Caribbean

Ecuador	Latin America and Caribbean
Egypt	North Africa and Middle East
El Salvador	Latin America and Caribbean
Equatorial Guinea	Sub-Saharan Africa
Eritrea	Sub-Saharan Africa
Estonia	Central Europe, Eastern Europe, and Central Asia
Ethiopia	Sub-Saharan Africa
Federated States of Micronesia	Southeast Asia, East Asia, and Oceania
Fiji	Southeast Asia, East Asia, and Oceania
Finland	High-income
France	High-income
Gabon	Sub-Saharan Africa
The Gambia	Sub-Saharan Africa
Georgia	Central Europe, Eastern Europe, and Central Asia
Germany	High-income
Ghana	Sub-Saharan Africa
Greece	High-income
Greenland	High-income
Grenada	Latin America and Caribbean
Guam	Southeast Asia, East Asia, and Oceania
Guatemala	Latin America and Caribbean
Guinea	Sub-Saharan Africa
Guinea-Bissau	Sub-Saharan Africa
Guyana	Latin America and Caribbean
Haiti	Latin America and Caribbean
Honduras	Latin America and Caribbean
Hungary	Central Europe, Eastern Europe, and Central Asia
Iceland	High-income
India	South Asia
Indonesia	Southeast Asia, East Asia, and Oceania
Iran	North Africa and Middle East
Iraq	North Africa and Middle East
Ireland	High-income
Israel	High-income
Italy	High-income
Jamaica	Latin America and Caribbean
Japan	High-income
Jordan	North Africa and Middle East
Kazakhstan	Central Europe, Eastern Europe, and Central Asia

Kenya	Sub-Saharan Africa
Kiribati	Southeast Asia, East Asia, and Oceania
Kuwait	North Africa and Middle East
Kyrgyzstan	Central Europe, Eastern Europe, and Central Asia
Laos	Southeast Asia, East Asia, and Oceania
Latvia	Central Europe, Eastern Europe, and Central Asia
Lebanon	North Africa and Middle East
Lesotho	Sub-Saharan Africa
Liberia	Sub-Saharan Africa
Libya	North Africa and Middle East
Lithuania	Central Europe, Eastern Europe, and Central Asia
Luxembourg	High-income
Macedonia	Central Europe, Eastern Europe, and Central Asia
Madagascar	Sub-Saharan Africa
Malawi	Sub-Saharan Africa
Malaysia	Southeast Asia, East Asia, and Oceania
Maldives	Southeast Asia, East Asia, and Oceania
Mali	Sub-Saharan Africa
Malta	High-income
Marshall Islands	Southeast Asia, East Asia, and Oceania
Mauritania	Sub-Saharan Africa
Mauritius	Southeast Asia, East Asia, and Oceania
Mexico	Latin America and Caribbean
Moldova	Central Europe, Eastern Europe, and Central Asia
Montenegro	Central Europe, Eastern Europe, and Central Asia
Mongolia	Central Europe, Eastern Europe, and Central Asia
Morocco	North Africa and Middle East
Mozambique	Sub-Saharan Africa
Myanmar	Southeast Asia, East Asia, and Oceania
Namibia	Sub-Saharan Africa
Nepal	South Asia
Netherlands	High-income
New Zealand	High-income
Nicaragua	Latin America and Caribbean
Niger	Sub-Saharan Africa
Nigeria	Sub-Saharan Africa
North Korea	Southeast Asia, East Asia, and Oceania
Northern Mariana Islands	Southeast Asia, East Asia, and Oceania
Norway	High-income

Oman	North Africa and Middle East
Pakistan	South Asia
Palestine	North Africa and Middle East
Panama	Latin America and Caribbean
Papua New Guinea	Southeast Asia, East Asia, and Oceania
Paraguay	Latin America and Caribbean
Peru	Latin America and Caribbean
Philippines	Southeast Asia, East Asia, and Oceania
Poland	Central Europe, Eastern Europe, and Central Asia
Portugal	High-income
Puerto Rico	Latin America and Caribbean
Qatar	North Africa and Middle East
Romania	Central Europe, Eastern Europe, and Central Asia
Russia	Central Europe, Eastern Europe, and Central Asia
Rwanda	Sub-Saharan Africa
Saint Lucia	Latin America and Caribbean
Saint Vincent and the Grenadines	Latin America and Caribbean
Samoa	Southeast Asia, East Asia, and Oceania
São Tomé and Príncipe	Sub-Saharan Africa
Saudi Arabia	North Africa and Middle East
Senegal	Sub-Saharan Africa
Serbia	Central Europe, Eastern Europe, and Central Asia
Seychelles	Southeast Asia, East Asia, and Oceania
Sierra Leone	Sub-Saharan Africa
Singapore	High-income
Slovakia	Central Europe, Eastern Europe, and Central Asia
Slovenia	Central Europe, Eastern Europe, and Central Asia
Solomon Islands	Southeast Asia, East Asia, and Oceania
Somalia	Sub-Saharan Africa
South Africa	Sub-Saharan Africa
South Korea	High-income
South Sudan	Sub-Saharan Africa
Spain	High-income
Sri Lanka	Southeast Asia, East Asia, and Oceania
Sudan	North Africa and Middle East
Suriname	Latin America and Caribbean
Swaziland	Sub-Saharan Africa
Sweden	High-income
Switzerland	High-income

Syria	North Africa and Middle East
Taiwan (province of China)	Southeast Asia, East Asia, and Oceania
Tajikistan	Central Europe, Eastern Europe, and Central Asia
Tanzania	Sub-Saharan Africa
Thailand	Southeast Asia, East Asia, and Oceania
Timor-Leste	Southeast Asia, East Asia, and Oceania
Togo	Sub-Saharan Africa
Tonga	Southeast Asia, East Asia, and Oceania
Trinidad and Tobago	Latin America and Caribbean
Tunisia	North Africa and Middle East
Turkey	North Africa and Middle East
Turkmenistan	Central Europe, Eastern Europe, and Central Asia
Uganda	Sub-Saharan Africa
Ukraine	Central Europe, Eastern Europe, and Central Asia
United Arab Emirates	North Africa and Middle East
United Kingdom	High-income
United States	High-income
Uruguay	High-income
Uzbekistan	Central Europe, Eastern Europe, and Central Asia
Vanuatu	Southeast Asia, East Asia, and Oceania
Venezuela	Latin America and Caribbean
Vietnam	Southeast Asia, East Asia, and Oceania
Virgin Islands, US	Latin America and Caribbean
Yemen	North Africa and Middle East
Zambia	Sub-Saharan Africa
Zimbabwe	Sub-Saharan Africa

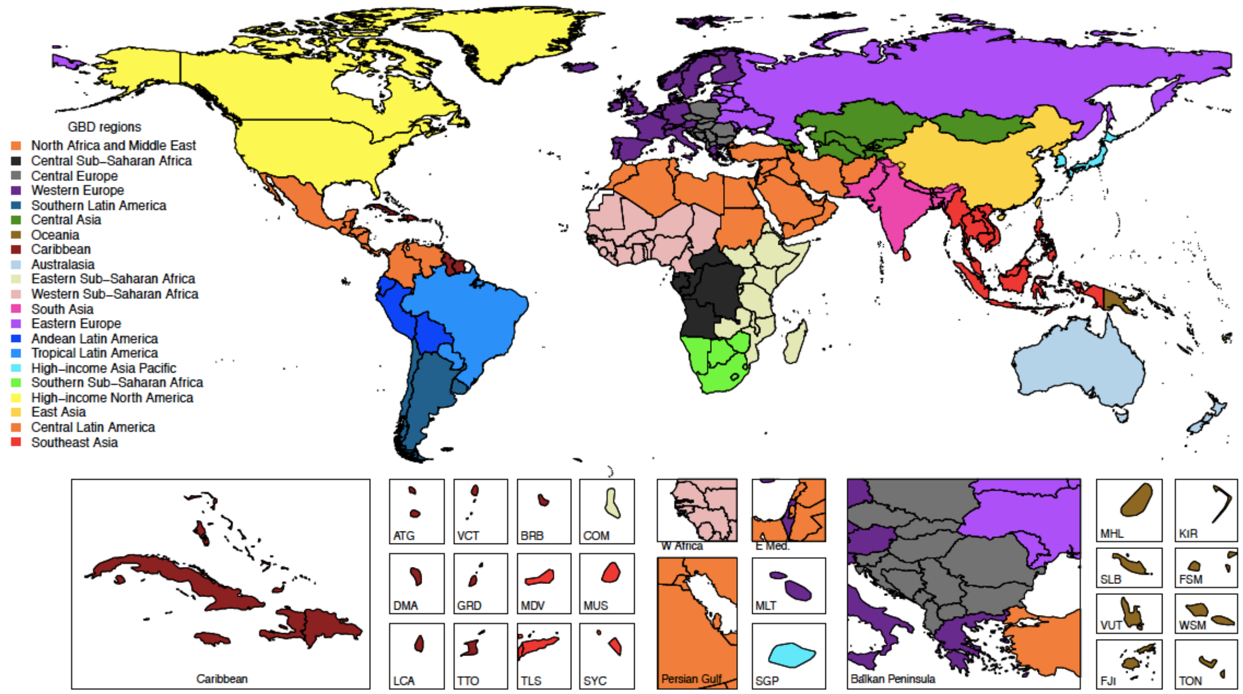

Appendix Figure 5: Map of GBD world regions, 2017. ATG: Antigua and Barbuda; VCT: Saint Vincent and the Grenadines; BRB: Barbados; COM: Comoros; W Africa: West Africa; E Med: Eastern Mediterranean; MHL: Marshall Islands; KIR: Kiribati; DMA: Dominica; GRD: Grenada; MDV: Maldives; MUS: Mauritius; MLT: Malta; SLB: Solomon Islands; FSM: Federated States of Micronesia; LCA: Saint Lucia; TTO: Trinidad and Tobago; TLS: Timor-Leste; SYC: Seychelles; SGP: Singapore; VUT: Vanuatu; WSM: Samoa; FJI: Fiji; TON: Tonga.

Appendix Table 9: Definition of GBD world regions, 2017

**Country name**	**GBD world region**
Afghanistan	North Africa and Middle East
Albania	Central Europe
Algeria	North Africa and Middle East
American Samoa	Oceania
Andorra	Western Europe
Angola	Central sub-Saharan Africa
Antigua and Barbuda	Caribbean
Argentina	Southern Latin America
Armenia	Central Asia
Australia	Australasia
Austria	Western Europe
Azerbaijan	Central Asia
The Bahamas	Caribbean
Bahrain	North Africa and Middle East

Bangladesh	South Asia
Barbados	Caribbean
Belarus	Eastern Europe
Belgium	Western Europe
Belize	Caribbean
Benin	Western sub-Saharan Africa
Bermuda	Caribbean
Bhutan	South Asia
Bolivia	Andean Latin America
Bosnia and Herzegovina	Central Europe
Botswana	Southern sub-Saharan Africa
Brazil	Tropical Latin America
Brunei	High-income Asia Pacific
Bulgaria	Central Europe
Burkina Faso	Western sub-Saharan Africa
Burundi	Eastern sub-Saharan Africa
Cambodia	Southeast Asia
Cameroon	Western sub-Saharan Africa
Canada	High-income North America
Cape Verde	Western sub-Saharan Africa
Central African Republic	Central sub-Saharan Africa
Chad	Western sub-Saharan Africa
Chile	Southern Latin America
China	East Asia
Colombia	Central Latin America
Comoros	Eastern sub-Saharan Africa
Congo	Central sub-Saharan Africa
Costa Rica	Central Latin America
Côte d'Ivoire	Western sub-Saharan Africa
Croatia	Central Europe
Cuba	Caribbean
Cyprus	Western Europe
Czech Republic	Central Europe
Democratic Republic of the Congo	Central sub-Saharan Africa
Denmark	Western Europe
Djibouti	Eastern sub-Saharan Africa
Dominica	Caribbean

Dominican Republic	Caribbean
Ecuador	Andean Latin America
Egypt	North Africa and Middle East
El Salvador	Central Latin America
Equatorial Guinea	Central sub-Saharan Africa
Eritrea	Eastern sub-Saharan Africa
Estonia	Eastern Europe
Ethiopia	Eastern sub-Saharan Africa
Federated States of Micronesia	Oceania
Finland	Western Europe
Fiji	Oceania
France	Western Europe
Gabon	Central sub-Saharan Africa
The Gambia	Western sub-Saharan Africa
Georgia	Central Asia
Germany	Western Europe
Ghana	Western sub-Saharan Africa
Greece	Western Europe
Greenland	High-income North America
Grenada	Caribbean
Guam	Oceania
Guatemala	Central Latin America
Guinea	Western sub-Saharan Africa
Guinea-Bissau	Western sub-Saharan Africa
Guyana	Caribbean
Haiti	Caribbean
Honduras	Central Latin America
Hungary	Central Europe
Iceland	Western Europe
India	South Asia
Indonesia	Southeast Asia
Iran	North Africa and Middle East
Iraq	North Africa and Middle East
Ireland	Western Europe
Israel	Western Europe
Italy	Western Europe
Jamaica	Caribbean
Japan	High-income Asia Pacific

Jordan	North Africa and Middle East
Kazakhstan	Central Asia
Kenya	Eastern sub-Saharan Africa
Kiribati	Oceania
Kuwait	North Africa and Middle East
Kyrgyzstan	Central Asia
Laos	Southeast Asia
Latvia	Eastern Europe
Lebanon	North Africa and Middle East
Lesotho	Southern sub-Saharan Africa
Liberia	Western sub-Saharan Africa
Libya	North Africa and Middle East
Lithuania	Eastern Europe
Luxembourg	Western Europe
Macedonia	Central Europe
Madagascar	Eastern sub-Saharan Africa
Malawi	Eastern sub-Saharan Africa
Malaysia	Southeast Asia
Maldives	Southeast Asia
Mali	Western sub-Saharan Africa
Malta	Western Europe
Marshall Islands	Oceania
Mauritania	Western sub-Saharan Africa
Mauritius	Southeast Asia
Mexico	Central Latin America
Moldova	Eastern Europe
Montenegro	Central Europe
Mongolia	Central Asia
Morocco	North Africa and Middle East
Mozambique	Eastern sub-Saharan Africa
Myanmar	Southeast Asia
Namibia	Southern sub-Saharan Africa
Nepal	South Asia
Netherlands	Western Europe
New Zealand	Australasia
Nicaragua	Central Latin America
Niger	Western sub-Saharan Africa
Nigeria	Western sub-Saharan Africa
North Korea	East Asia

Northern Mariana Islands	Oceania
Norway	Western Europe
Oman	North Africa and Middle East
Pakistan	South Asia
Palestine	North Africa and Middle East
Panama	Central Latin America
Papua New Guinea	Oceania
Paraguay	Tropical Latin America
Peru	Andean Latin America
Philippines	Southeast Asia
Poland	Central Europe
Portugal	Western Europe
Puerto Rico	Caribbean
Qatar	North Africa and Middle East
Romania	Central Europe
Russia	Eastern Europe
Rwanda	Eastern sub-Saharan Africa
Saint Lucia	Caribbean
Saint Vincent and the Grenadines	Caribbean
Samoa	Oceania
São Tomé and Príncipe	Western sub-Saharan Africa
Saudi Arabia	North Africa and Middle East
Senegal	Western sub-Saharan Africa
Serbia	Central Europe
Seychelles	Southeast Asia
Sierra Leone	Western sub-Saharan Africa
Singapore	High-income Asia Pacific
Slovakia	Central Europe
Slovenia	Central Europe
Solomon Islands	Oceania
Somalia	Eastern sub-Saharan Africa
South Africa	Southern sub-Saharan Africa
South Korea	High-income Asia Pacific
South Sudan	Eastern sub-Saharan Africa
Spain	Western Europe
Sri Lanka	Southeast Asia
Suriname	Caribbean

Swaziland	Southern sub-Saharan Africa
Sweden	Western Europe
Switzerland	Western Europe
Sudan	North Africa and Middle East
Syria	North Africa and Middle East
Taiwan (province of China)	East Asia
Tajikistan	Central Asia
Tanzania	Eastern sub-Saharan Africa
Thailand	Southeast Asia
Timor-Leste	Southeast Asia
Togo	Western sub-Saharan Africa
Tonga	Oceania
Trinidad and Tobago	Caribbean
Tunisia	North Africa and Middle East
Turkey	North Africa and Middle East
Turkmenistan	Central Asia
Uganda	Eastern sub-Saharan Africa
Ukraine	Eastern Europe
United Arab Emirates	North Africa and Middle East
United Kingdom	Western Europe
United States	High-income North America
Uruguay	Southern Latin America
Uzbekistan	Central Asia
Vanuatu	Oceania
Venezuela	Central Latin America
Vietnam	Southeast Asia
Virgin Islands, US	Caribbean
Yemen	North Africa and Middle East
Zambia	Eastern sub-Saharan Africa
Zimbabwe	Southern sub-Saharan Africa

Additional Results in Tables & Figures

Appendix Table 10: Adult cancer DALY burden by SDI quintile, GBD 2017

Location	Absolute DALYs	95% uncertainty interval	Percentage of global burden
High SDI	53,239,000	(52,071,800–54,565,900)	24.4%
High-middle SDI	54,083,600	(52,171,400–55,908,700)	24.8%
Middle SDI	59,939,500	(57,979,300–61,831,500)	27.5%
Low-middle SDI	31,306,600	(29,787,200–33,182,200)	14.4%
Low SDI	18,424,800	(17,440,000–19,288,400)	8.4%
Global	218,153,300	(214,123,078–222,408,900)	100.0%

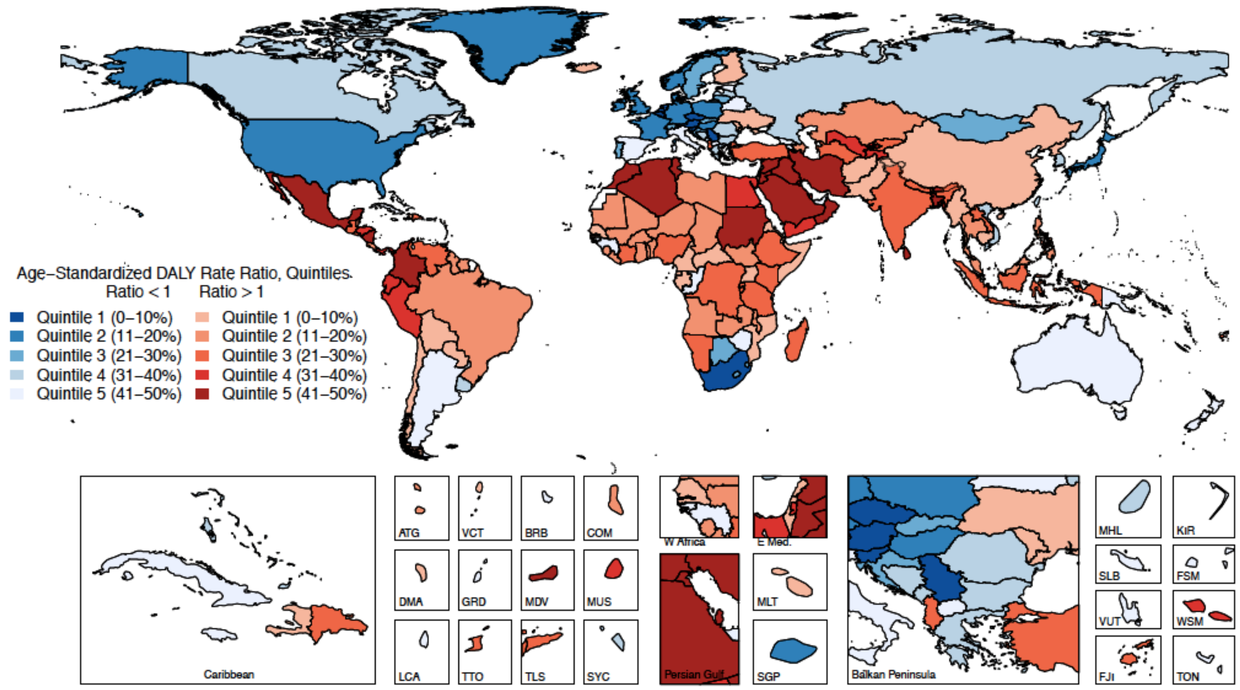

Appendix Figure 6: Global map of ratio of childhood to adult age-standardised DALY rates of all combined malignant cancers for both sexes combined in 2017. Ratio was calculated as childhood cancer age-standardised DALY rate (numerator) to adult cancer age-standardised DALY rate (denominator). Ratio < 1 indicates countries in which childhood cancer age-standardised DALY rates are less than adult cancer age-standardised DALY rates. Ratio > 1 indicates countries in which childhood cancer age-standardised DALY rates are greater than adult cancer age-standardised DALY rates. For Ratio < 1: Quintile 1 (0-10%): < 0.25, Quintile 2 (11-20%): 0.25 to < 0.42, Quintile 3 (21-30%): 0.42 to < 0.56, Quintile 4 (31-40%): 0.56 to < 0.74, Quintile 5 (41-50%): 0.74 to < 1. For Ratio > 1: Quintile 1 (0-10%): 1 to < 1.31, Quintile 2 (11-20%): 1.31 to < 1.88, Quintile 3 (21-30%): 1.88 to < 3.02, Quintile 4 (31-40%): 3.02 to < 6.58, Quintile 5 (41-50%): ≥ 6.58. Adult cancer burden portrayed in this figure excluded non-melanoma skin cancers in order to be comparable to the childhood cancer burden map. ATG: Antigua and Barbuda; VCT: Saint Vincent and the Grenadines; BRB: Barbados; COM: Comoros; W Africa: West Africa; E Med: Eastern Mediterranean; MHL: Marshall Islands; KIR: Kiribati; DMA: Dominica; GRD: Grenada; MDV: Maldives; MUS: Mauritius; MLT: Malta; SLB: Solomon Islands; FSM: Federated States of Micronesia; LCA: Saint Lucia; TTO: Trinidad and Tobago; TLS: Timor-Leste; SYC: Seychelles; SGP: Singapore; VUT: Vanuatu; WSM: Samoa; FJI: Fiji; TON: Tonga.

Appendix Table 11: Total Global YLL, YLD, and DALY Burden by 5-Year Childhood Age Group in 2017

Age Groups	0 to 4	5 to 9	10 to 14	15 to 19
Total YLLs	4,132,200 (3,681,700-4,564,600)	2,534,600 (2,262,500-2,774,300)	2,160,100 (1,874,900 - 2,458,300)	2,409,600 (2,250,900-2,544,900)
Total YLDs	137,300 (83,700-214,400)	83,900 (52,600-129,700)	46,100 (31,100-65,000)	45,900 (32,400-62,600)
Total DALYs	4,269,400 (3,805,200-4,721,600)	2,618,500 (2,344,600-2,868,300)	2,206,200 (2,023,800-2,360,800)	2,455,500 (2,291,800-2,589,700)

Age groups refer to years. Values in parentheses are 95% uncertainty intervals.

High SDI

(a)

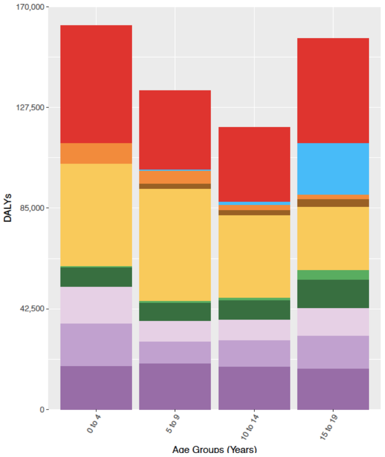

(b)

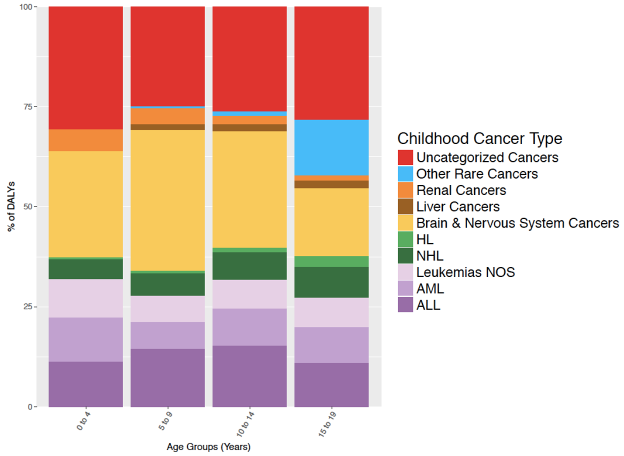

High-Middle SDI

(a)

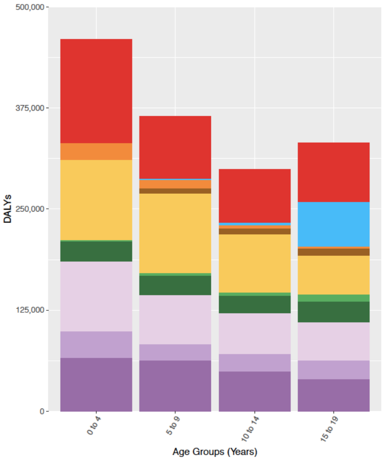

(b)

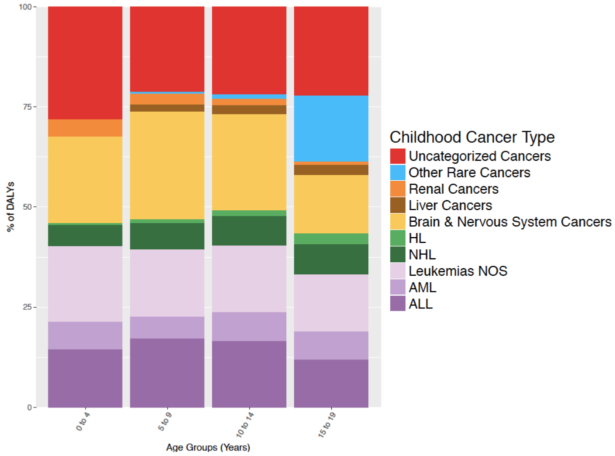

Middle SDI

(a)

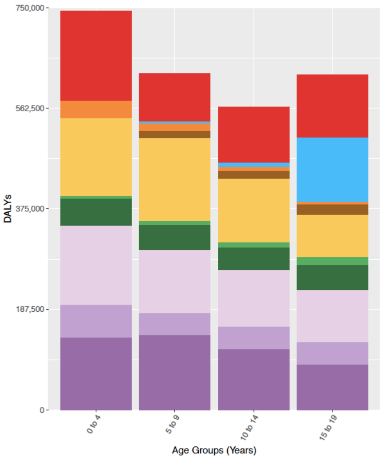

(b)

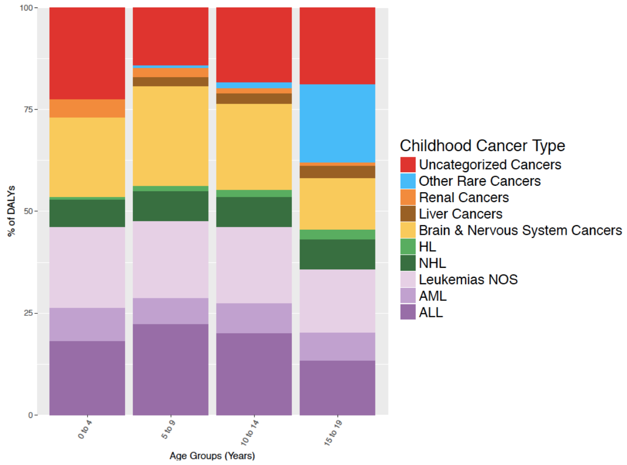

Low-Middle SDI

(a)

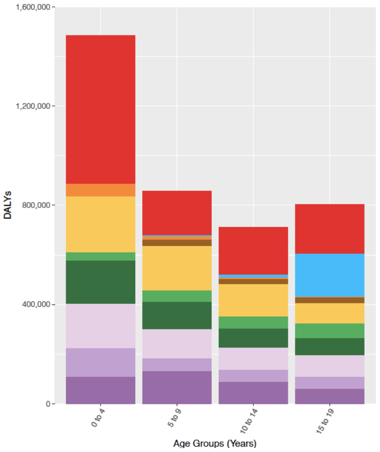

(b)

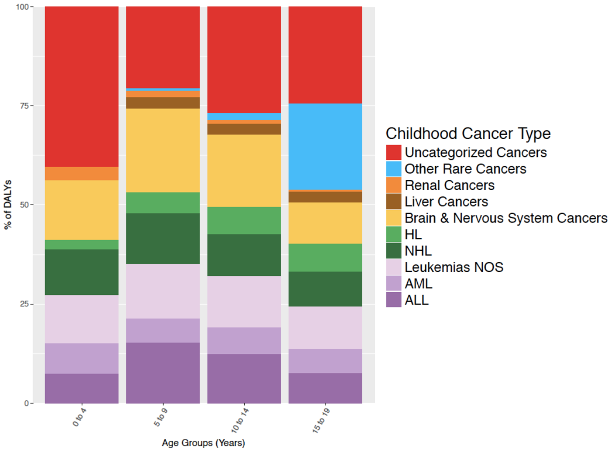

Low SDI

(a)

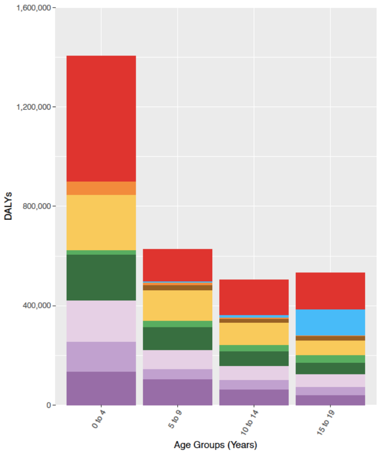

(b)

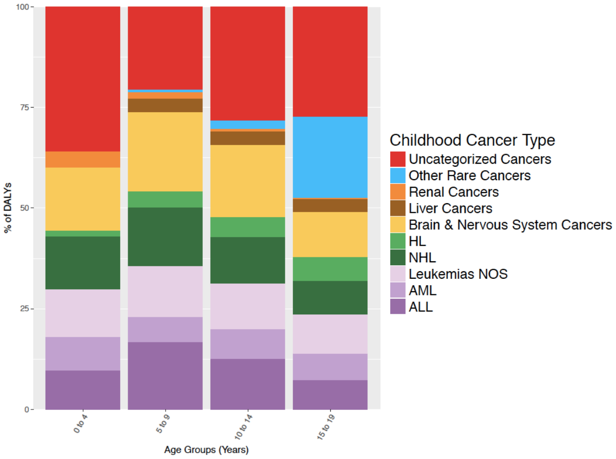

Appendix Figure 7: Global DALY burden by childhood cancer types, both sexes, 2017, in (a) absolute and (b) proportional 0- to 19-year-old DALY burden, by five-year age group and Socio-demographic Index (SDI) quintile. ALL=acute lymphoblastic leukaemia; AML=acute myeloid leukaemia; Leukaemias NOS=leukaemias not otherwise specified, chronic lymphocytic leukaemias (CLL) or chronic myeloid leukaemias (CML). NHL=non-Hodgkin lymphomas; HL=Hodgkin lymphomas; Other rare cancers=cancers with < 1000 total deaths globally in 2017; Uncategorised cancers=cancers without a detailed GBD cause.

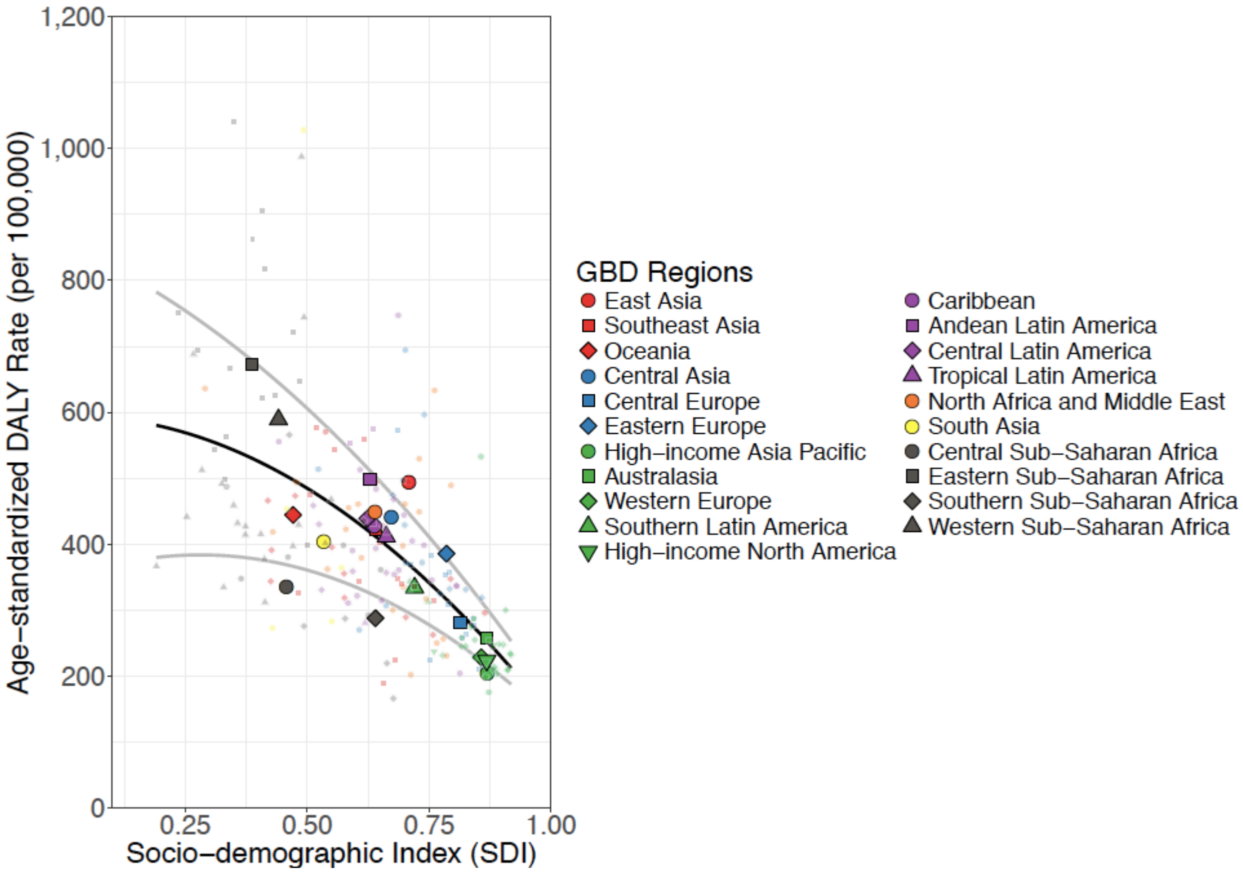

Appendix Figure 8: The relationship between Socio-demographic Index (SDI) and childhood cancer DALY rates in 2017. This figure represents estimates for both sexes combined. Each colour represents one of the seven GBD super-regions (red: Southeast Asia, East Asia, and Oceania; blue: Central Europe, eastern Europe, and central Asia; green: High-income; purple: Latin America and Caribbean; orange: North Africa and Middle East; yellow: South Asia; grey: Sub-Saharan Africa). For definitions of GBD world regions and super-regions see appendix pages 60 and 54, respectively. GBD region point estimates are median overall childhood cancer DALY rates due to inter-region variability. Lighter-coloured point estimates without labels in the legend represent countries. Country estimates are mean overall childhood cancer DALY rates. The black line represents locally weighted smoothing based on country-level data, and the grey lines represent locally weighted smoothing of country-level 95% uncertainty intervals.

References

1. Global Burden of Disease Cancer Collaboration. Global, Regional, and National Cancer Incidence, Mortality, Years of Life Lost, Years Lived With Disability, and Disability-Adjusted Life-years for 32 Cancer Groups, 1990 to 2015: A Systematic Analysis for the Global Burden of Disease Study. *JAMA Oncol* 2017; **3**(4): 524-48.
2. Global Burden of Disease Cancer Collaboration. Global, Regional, and National Cancer Incidence, Mortality, Years of Life Lost, Years Lived With Disability, and Disability-Adjusted Life-Years for 29 Cancer Groups, 1990 to 2016: A Systematic Analysis for the Global Burden of Disease Study. *JAMA Oncol* 2018; **4**(11): 1553-68.
3. Foreman KJ, Lozano R, Lopez AD, Murray CJ. Modeling causes of death: an integrated approach using CODEm. *Population health metrics* 2012; **10**: 1-23.
4. GBD 2017 Causes of Death Collaborators. Global, regional, and national age-sex-specific mortality for 282 causes of death in 195 countries and territories, 1980-2017: a systematic analysis for the Global Burden of Disease Study 2017. *Lancet* 2018; **392**(10159): 1736-88.
5. GBD 2017 Disease and Injury Incidence and Prevalence Collaborators. Global, regional, and national incidence, prevalence, and years lived with disability for 354 diseases and injuries for 195 countries and territories, 1990-2017: a systematic analysis for the Global Burden of Disease Study 2017. *Lancet* 2018; **392**(10159): 1789-858.
6. Doll R. Cancer in five continents. *Proc R Soc Med* 1972; **65**(1): 49-55.
7. Doll R, Muir C, Waterhouse J. Cancer Incidence in Five Continents II. Geneva: Union Internationale Contre le Cancer, 1970.
8. Waterhouse J, Muir C, Correa P, Powell J. Cancer Incidence in Five Continents III. Lyon: IARC, 1976.
9. Waterhouse J, Muir C, Shanmugaratnam K, Powell J. Cancer Incidence in Five Continents IV. Lyon: IARC, 1982.
10. Muir C, Mack T, Powell J, Whelan S. Cancer Incidence in Five Continents V. Lyon: IARC, 1987.
11. Parkin D, Muir C, Whelan S, Gao Y, Ferlay J, Powell J. Cancer Incidence in Five Continents VI. Lyon: IARC, 1992.
12. Parkin D, Whelan S, Ferlay J, Raymond L, Young J. Cancer Incidence in Five Continents VII. Lyon: IARC, 1997.
13. Parkin D, Whelan S, Ferlay J, Teppo L, Thomas D. Cancer Incidence in Five Continents VIII. Lyon: IARC, 2002.
14. Curado M, Edwards B, Shin H, et al. Cancer Incidence in Five Continents IX: IARC, 2007.
15. Forman D, Bray F, Brewster D, et al. Cancer Incidence in Five Continents X. Lyon: IARC, 2014.
16. Bray F CM, Mery L, Piñeros M, Znaor A, Zanetti R and Ferlay J, editors. Cancer Incidence in Five Continents, Vol. XI (electronic version). Lyon: International Agency for Research on Cancer, 2017.
17. Steliarova-Foucher E, O'Callaghan M, Ferlay J, et al. The European Cancer Observatory: A new data resource. *Eur J Cancer* 2015; **51**(9): 1131-43.
18. Engholm G, Ferlay J, Christensen N, et al. NORDCAN--a Nordic tool for cancer information, planning, quality control and research. *Acta Oncol* 2010; **49**(5): 725-36.

19. GBD 2016 Healthcare Access and Quality Collaborators. Measuring performance on the Healthcare Access and Quality Index for 195 countries and territories and selected subnational locations: a systematic analysis from the Global Burden of Disease Study 2016. *Lancet* 2018; **391**(10136): 2236-71.
20. GBD 2017 Risk Factor Collaborators. Global, regional, and national comparative risk assessment of 84 behavioural, environmental and occupational, and metabolic risks or clusters of risks for 195 countries and territories, 1990-2017: a systematic analysis for the Global Burden of Disease Study 2017. *Lancet* 2018; **392**(10159): 1923-94.
21. Asadzadeh Vostakolaei F, Karim-Kos HE, Janssen-Heijnen ML, Visser O, Verbeek AL, Kiemeney LA. The validity of the mortality to incidence ratio as a proxy for site-specific cancer survival. *Eur J Public Health* 2011; **21**(5): 573-7.
22. SEER*Stat Software. 2014. http://seer.cancer.gov/seerstat/.
23. Surveillance, Epidemiology, and End Results (SEER) Program (www.seer.cancer.gov) SEER*Stat Database: Incidence - SEER 18 Regs Research Data + Hurricane Katrina Impacted Louisiana Cases, Nov 2012 Sub (1973-2010 varying) - Linked To County Attributes - Total U.S., 1969-2011 Counties, National Cancer Institute, DCCPS, Surveillance Research Program, Surveillance Systems Branch, released April 2013, based on the November 2012 submission.
24. Sankaranarayanan R. Cancer survival in Africa, Asia, the Caribbean and Central America. Introduction. *IARC Sci Publ* 2011; (162): 1-5.
25. US Mortality Files: 61-Year Trends in U.S. Cancer Death Rates: National Center for Health Statistics, Centers for Disease Control and, Prevention.
26. SEER Cancer Statistics Review 1975-2011.
27. Dicker D, Nguyen G, Abate D, et al. Global, regional, and national age-sex-specific mortality and life expectancy, 1950-2017: a systematic analysis for the Global Burden of Disease Study 2017. *The Lancet* 2018; **392**(10159): 1684-735.
28. Neal RD, Din NU, Hamilton W, et al. Comparison of cancer diagnostic intervals before and after implementation of NICE guidelines: analysis of data from the UK General Practice Research Database. *Br J Cancer* 2014; **110**(3): 584-92.
29. Allgar VL, Neal RD. Delays in the diagnosis of six cancers: analysis of data from the National Survey of NHS Patients: Cancer. *Br J Cancer* 2005; **92**(11): 1959-70.
30. Neal RD, Cannings-John R, Hood K, et al. Excision of malignant melanomas in North Wales: effect of location and surgeon on time to diagnosis and quality of excision. *Fam Pract* 2008; **25**(4): 221-7.
31. Kewalramani T, Nimer SD, Zelenetz AD, et al. Progressive disease following autologous transplantation in patients with chemosensitive relapsed or primary refractory Hodgkin's disease or aggressive non-Hodgkin's lymphoma. *Bone Marrow Transplant* 2003; **32**(7): 673-9.
32. Esteban D, Tovar N, Jimenez R, et al. Patients with relapsed/refractory chronic lymphocytic leukaemia may benefit from inclusion in clinical trials irrespective of the therapy received: a case-control retrospective analsysis. *Blood cancer journal* 2015; **5**: e356.
33. Canova C, Giorato E, Roveron G, Turrini P, Zanotti R. Validation of a stoma-specific quality of life questionnaire in a sample of patients with colostomy or ileostomy. *Colorectal Dis* 2013; **15**(11): e692-8.

34. Caricato M, Ausania F, Ripetti V, Bartolozzi F, Campoli G, Coppola R. Retrospective analysis of long-term defunctioning stoma complications after colorectal surgery. *Colorectal Dis* 2007; **9**(6): 559-61.
35. Erwin-Toth P, Thompson SJ, Davis JS. Factors impacting the quality of life of people with an ostomy in North America: results from the Dialogue Study. *J Wound Ostomy Continence Nurs* 2012; **39**(4): 417-22; quiz 23-4.
36. Catalona WJ, Carvalhal GF, Mager DE, Smith DS. Potency, continence and complication rates in 1,870 consecutive radical retropubic prostatectomies. *J Urol* 1999; **162**(2): 433-8.
37. Donnellan SM, Duncan HJ, MacGregor RJ, Russell JM. Prospective assessment of incontinence after radical retropubic prostatectomy: objective and subjective analysis. *Urology* 1997; **49**(2): 225-30.
38. Eastham JA, Kattan MW, Rogers E, et al. Risk factors for urinary incontinence after radical prostatectomy. *J Urol* 1996; **156**(5): 1707-13.
39. Kundu SD, Roehl KA, Eggener SE, Antenor JA, Han M, Catalona WJ. Potency, continence and complications in 3,477 consecutive radical retropubic prostatectomies. *J Urol* 2004; **172**(6 Pt 1): 2227-31.
40. Potosky AL, Davis WW, Hoffman RM, et al. Five-year outcomes after prostatectomy or radiotherapy for prostate cancer: the prostate cancer outcomes study. *J Natl Cancer Inst* 2004; **96**(18): 1358-67.
41. Sacco E, Prayer-Galetti T, Pinto F, et al. Urinary incontinence after radical prostatectomy: incidence by definition, risk factors and temporal trend in a large series with a long-term follow-up. *BJU Int* 2006; **97**(6): 1234-41.
42. Stanford JL, Feng Z, Hamilton AS, et al. Urinary and sexual function after radical prostatectomy for clinically localized prostate cancer: the Prostate Cancer Outcomes Study. *JAMA* 2000; **283**(3): 354-60.
43. Walsh PC, Marschke P, Ricker D, Burnett AL. Patient-reported urinary continence and sexual function after anatomic radical prostatectomy. *Urology* 2000; **55**(1): 58-61.
44. GBD 2017 DALYs and HALE Collaborators. Global, regional, and national disability-adjusted life-years (DALYs) for 359 diseases and injuries and healthy life expectancy (HALE) for 195 countries and territories, 1990-2017: a systematic analysis for the Global Burden of Disease Study 2017. *Lancet* 2018; **392**(10159): 1859-922.
45. GBD 2017 Mortality Collaborators. Global, regional, and national age-sex-specific mortality and life expectancy, 1950-2017: a systematic analysis for the Global Burden of Disease Study 2017. *Lancet* 2018; **392**(10159): 1684-735.
46. Vasudevan S, Ramos F, Nettleton E, Durrant-Whyte H, Blair A. Gaussian Process modeling of large scale terrain. 2009 IEEE International Conference on Robotics and Automation; 2009 12-17 May 2009; 2009. p. 1047-53.
47. Rasmussen CE, Williams CKI. Gaussian Processes for Machine Learning (Adaptive Computation and Machine Learning): The MIT Press; 2005.
48. GBD 2015 Risk Factors Collaborators. Global, regional, and national comparative risk assessment of 79 behavioural, environmental and occupational, and metabolic risks or clusters of risks, 1990-2015: a systematic analysis for the Global Burden of Disease Study 2015. *Lancet* 2016; **388**(10053): 1659-724.
49. Ng M, Freeman MK, Fleming TD, et al. Smoking prevalence and cigarette consumption in 187 countries, 1980-2012. *JAMA* 2014; **311**(2): 183-92.

50. Ng M, Fleming T, Robinson M, et al. Global, regional, and national prevalence of overweight and obesity in children and adults during 1980-2013: a systematic analysis for the Global Burden of Disease Study 2013. *Lancet* 2014; **384**(9945): 766-81.
51. Bell RM, Koren Y. Lessons from the Netflix prize challenge. *SIGKDD Explor Newsl* 2007; **9**(2): 75-9.
52. Bell RM, Koren Y, Volinsky C. All Together Now: A Perspective on the Netflix Prize. *CHANCE* 2010; **23**(1): 24-9.
